# Buffer and Salt Effects in Aqueous Host–Guest Systems: Screening, Competitive Binding, or Both?

**DOI:** 10.1021/jacs.1c08457

**Published:** 2021-10-27

**Authors:** Jacobs H. Jordan, Henry S. Ashbaugh, Joel T. Mague, Bruce C. Gibb

**Affiliations:** † Agricultural Research Service Southern Regional Research Center, U.S. Department of Agriculture, New Orleans, Louisiana 70124, United States; ‡ Department of Chemical and Biomolecular Engineering, Tulane University, New Orleans, Louisiana 70118, United States; § Department of Chemistry, Tulane University, New Orleans, Louisiana 70118, United States

## Abstract

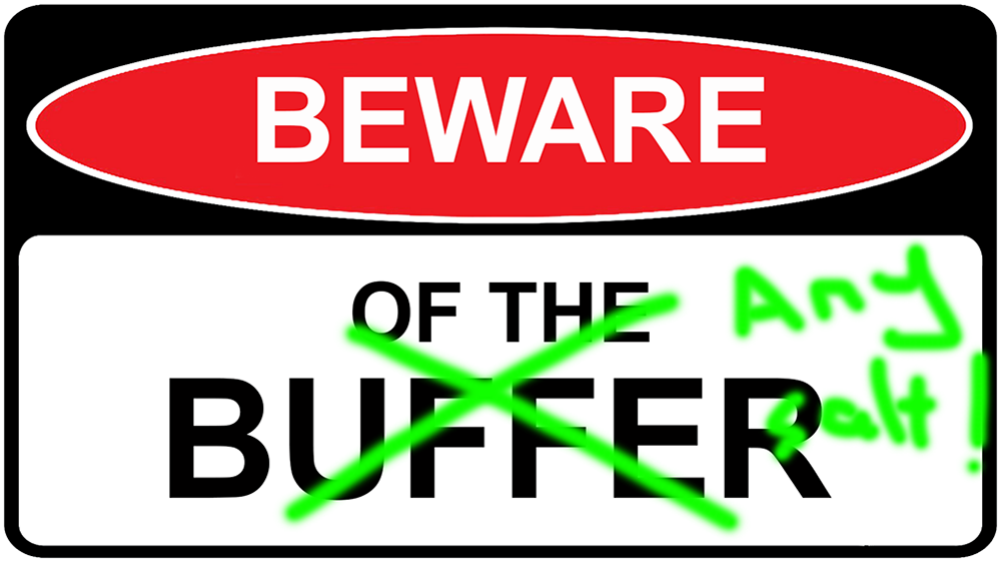

There are many open questions regarding the supramolecular properties of ions in water, a fact that has ramifications within any field of study involving buffered solutions. Indeed, as Pielak has noted (Buffers, Especially the Good Kind, *Biochemistry*, **2021**, in press. DOI:10.1021/acs.biochem.1c00200) buffers were conceived of with little regard to their supramolecular properties. But there is a difficulty here; the mathematical models supramolecular chemists use for affinity determinations do not account for screening. As a result, there is uncertainty as to the magnitude of any screening effect and how this compares to competitive salt/buffer binding. Here we use a tetra-cation cavitand to compare halide affinities obtained using a traditional unscreened model and a screened (Debye–Hückel) model. The rule of thumb that emerges is that if ionic strength is changed by >1 order of magnitude—either during a titration or if a comparison is sought between two different buffered solutions—screening should be considered. We also build a competitive mathematical model showing that binding attenuation in buffer is largely due to competitive binding to the host by said buffer. For the system at hand, we find that the effect of competition is approximately twice that of the effect of screening (∼*RT* at 25 °C). Thus, for strong binders it is less important to account for screening than it is to account for competitive complexation, but for weaker binders both effects should be considered. We anticipate these results will help supramolecular chemists unravel the properties of buffers and so help guide studies of biomacromolecules.

## Introduction

Although affinity determinations in organic media can successfully treat the solvent as purely a spectator, in aqueous supramolecular chemistry^1^ water can seldom, if ever, be ignored. There are multiple reasons as to why this is so, all tied to the small size and high cohesiveness of water, making water–solute interactions strongly context dependent.^2−6^ This context dependency means that there are still many open questions regarding the intricate solvation of both nonpolar surfaces^5−7^ and charged species.^8,9^ Regarding the latter, although the classical treatment of electrostatic interactions in solution based on the Poisson–Boltzmann equation is routine,^10^ and the net hydration thermodynamics of common ions are known,^11^ the map of the plasticity of the solvation shells of ions, and hence the manner in which they can (move aside their solvation shell and) interact noncovalently with other chemical entities, has yet to be drawn, despite the ubiquity of buffers in the biosciences^12,13^ and the appreciation that they interact with other ions^14−17^ and biomacromolecules.^18−21^ By probing and mapping the supramolecular properties of buffers and more generally ions, supramolecular chemists can assist the biological sciences in their studies of biomacromolecules.

But there is a problem here regarding the phenomenon of screening. The model typically used by supramolecular chemists for obtaining affinity constants does not account for it. Is this realistic? Or is screening at the root of the common observation that a measured affinity is dependent on the nature and concentration of a buffer? If so, models based on Debye–Hückel theory that account for it would be preferred when determining affinities.

Screening theoretically affects affinity determinations in both slow and fast exchanging host–guest systems, but in slightly different ways. Consider first a slow-exchanging system. In these, techniques such as NMR spectroscopy yield the affinity constant by measuring the equilibrated concentrations of host, guest, and complex at one host–guest ratio. A key question here is do screening changes cause the usually observed affinity differences between an unbuffered and a buffered system, or alternatively, the differences in measured affinity in two different buffered systems? There’s a further complication in commonly encountered fast-exchanging systems. In these, affinity determinations require host–guest titration experiments in which the ratio of host to guest is varied as a dependent variable is measured. Since in most cases such determinations involve the binding of anions by polycationic hosts,^22,23^ or the binding of cations to polyanionic hosts,^22,24^ the ionic strength (*I*) of the solution changes during titration. Do these changes in *I* lead to significant changes in screening?

All this noted, there is another possibility as to why affinity determinations frequently differ from one buffered solution to the next: competitive buffer binding to the host. This possibility raises serious concerns. Many design principles^18^ went into Good’s buffers commonplace in contemporary laboratories,^12,13^ but avoiding supramolecular properties was not high on the agenda (beyond the likes of amine–metal coordination). Indeed it was not until 1980 that Good noted,^25^ “it is almost impossible to find buffering substances which have no physiological effects of their own. All have effects which are unrelated to pH stabilization.” Moreover, beyond Good’s buffers one of the most heavily utilized buffers today is TRIS, a compound known to interact with proteins,^21^ and, to a supramolecular chemist’s eye, a suspect, greasy cation (at pH = 7) and a triple-hydrogen-bonded chelator ripe for anions and other hydrogen bond acceptors. So which buffers behave purely as spectators and which have strong supramolecular properties that can interfere with the intent of an experiment?

To summarize the points made thus far, supramolecular chemists do not consider screening in host–guest determinations. And most users of buffers do not think of them as competing guests. Yet both phenomena—screening and buffer complexation—may play a role in affinity determinations or indeed any other experimental dependent variable linked to intermolecular interactions. And what is the balance between the effects of screening and supramolecular properties of ions and buffers? It would benefit multiple fields immensely to accurately measure affinities and use these to construct guidelines laying out which ions and buffers have minimum supramolecular properties and under which conditions each can be safely used. As Smith succinctly concluded,^26^ “it is clear that supramolecular chemists need to increasingly think very carefully about the environment in which molecular recognition is taking place.”^27,28^

**Scheme 1:**
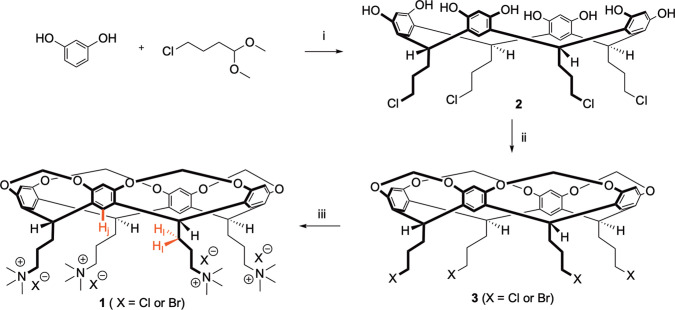
Synthesis of Water-Soluble Receptor **1** Reagents and conditions: (i) HCl/MeOH (10:3), 0 °C, 30 min, then 55 °C 5 d; (ii) K_2_CO_3_, DMA, CH_2_BrCl, 55 °C, 7 d; (iii) N(CH_3_)_3_, DMF/H_2_O (10:1), 70 °C, 3 d.

Toward formulating the importance of screening and/or competitive binding effects in aqueous supramolecular chemistry, we examine here the fast-exchanging complexation of anions to tetra-cationic cavitand **1** (Scheme 1).

(1) Specifically, we use ^1^H NMR spectroscopy to probe the binding of halides (F^–^, Cl^–^, Br^–^, and I^–^) to cavitand **1** and compare the affinity constants calculated using the standard mathematical model that ignores screening with a Debye–Hückel model that considers it. We define the former and latter as  and , where the superscript *U*,0 or *S*,0 denotes an unscreened or screened model measured relative to the reference concentration, and *X*^*–*^ corresponds to the nature of the guest. We demonstrate that screening does make a significant difference in the calculated affinities, but only from the perspective of affinity constants. In terms of free energy, the differences are small (∼(1/2)*RT* at 25 °C). Additionally, use of the Debye–Hückel model also demonstrates that only in the case of weakly binding guests, where changes in ionic strength during a host–guest titration are necessarily large, do changes in screening significantly affect affinity and speciation.

(2) Following this, we determine the affinity of the buffer species HPO_4_^2–^ and H_2_PO_4_^–^ to **1** and determine the halide affinity for **1** in three buffer systems involving these two species. Mindful of the conclusion from (1), we use the unscreened model to yield observed affinities (*K*_obs_^U,0^). We observe global attenuation of affinity values arising from the use of buffer. Concomitantly, we show that these attenuated *K*_obs_^U,0^ values can be predicted *a priori* from a model based only on the obtained  values and the assumption that the attenuation of affinity is entirely due to competitive anion binding to the host. That these obtained *K*_pred_^U,0^ values closely match the *K*_obs_^U,0^ values demonstrates that simple competition for the host causes the attenuation of affinity observed in the buffer. Importantly, in the system at hand this competition effect is double (∼*RT* at 25 °C) that of any screening effect.

Taken together, these results reveal that the routine application of fitting models that ignore screening is reasonable from the perspective of the free energy of guest binding, but that in terms of equilibrium constant values, unscreened models will lead to calculated affinities somewhat lower than if screening is factored in. However, in the case of weak binding guests that involve a large change in ionic strength during titration, screening effects cannot be ignored. Additionally, our experiments reveal that the generally larger changes in affinity observed between unbuffered versus buffered solutions are mostly due to simple direct guest competition for the host.

We anticipate that these findings will help address the uncertainly often associated with binding constant determinations in water and buffered solutions and contribute to the long-term goal of understanding the supramolecular properties of buffers and ions in general.

## Experimental Section

Host **1** was synthesized as shown in Scheme 1. Briefly, synthesis of resorcinarene **2** in 95% yield was achieved by the acid-catalyzed condensation of resorcinol and 4-chlorobutanal dimethyl acetal.^29^ This was then bridged with bromochloromethane in 20% yield to yield cavitand **3**. In this bridging reaction a degree of halogen exchange was noted to occur at the pendent groups, but this replacement of chloride for bromide only enhanced the rate of the subsequent step. Thus, a Menshutkin reaction gave the desired tetrakis(trimethylammonium) halide **1** (TMAX) in 60% yield as a mixed salt (X = Cl^–^ and Br^–^). Ion exchange gave the tetrachloride salt TMAX-Cl, **1**. Full synthetic details are given in the SI (Section 2.A).

As we discuss below, the aromatic bowl of TMAX-Cl **1** acts purely as a scaffold; anion binding to **1** occurs in the “crown” of four ammonium groups formed by the pendent groups of the cavitand. Unless expressed otherwise, we utilized TMAX-Cl **1** as the host.

## Results and Discussion

### Host Design

We selected TMAX-Cl **1** as the principle host in this study both because of its ready synthesis (see above) and because in studies with an analogous but more complex host we had observed well-characterizable anion affinity to the crown of four ammonium groups.^30^ Although the affinity of large, more charge-diffuse anions was relatively strong, halide affinity was much weaker, ranging from 120 to 3200 M^–1^. Thus, we concluded that for reliable halide affinity determinations in competitive water, the crown of four ammonium groups represented close to the minimum supramolecular motif that could be successfully utilized.

### The Role of Screening: Unscreened and Screened (Debye–Hückel) Models

The titration of a charged guest into a solution of host **1** or simply an increase in buffer concentration leads to an increase in the ionic strength of the solution. Conceivably, this leads to two separate effects: a change in the dielectric of the medium and a change in the double layer of ions around the charged host and guest. However, physical models do not typically separate these two concepts. Rather, it is more convenient to merge both into a single screening effect. This is the position we take here.

In electrolyte mixtures, charged species will adopt distributions that screen long-range Coulombic interactions. Screening, in turn, moderates the interactions between charged host and guest. For low salt concentrations (∼0.1 M or less), the effect of charge screening on the free energy of charged species in solution can be modeled using Debye–Hückel limiting theory.^31,32^ Following this theory, the partial molar Gibbs free energy of charged component *i*, *G̅*_*i*_, is determined as
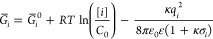
1where *G̅*_*i*_^0^ is the free energy of *i* in the absence of screening (κ = 0) measured at the reference concentration *C*_0_, [*i*] is the concentration of *i*, *RT* is the product of the gas constant and the absolute temperature, κ^–1^ is the Debye length describing the thickness of the counterion double layer that screens electrostatic interactions, *q*_*i*_ is the charge of *i*, σ_*i*_ is the Born radius (the ion-excluding radius) of *i*, ε_0_ is the permittivity of free space, and ε is the dielectric constant of the solvent (water).^33^ The inverse Debye screening length κ is defined by
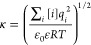
2where the sum extends over all charged species *i* (in this case, host **1**, the anionic guest, and the nonassociating anions and cations). While we appreciate that Debye–Hückel theory best describes similarly sized monovalent ions, we adopt this theory here to describe host–guest association to qualitatively assess the impact of charge screening on the binding process.

For a monovalent anionic guest (X^–^) complexing with a tetravalent cationic host **1**, equilibrium is governed by the reaction
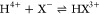
3For the host–guest complexations described here, the free energy of the system is minimized when
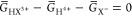
4Of course, eq 4 can be readily written in general form for all complexation processes. In the absence of screening (κ = 0, i.e., the double layer thickness is infinite), substituting expressions for the partial molar Gibbs free energies of host (H), anion (X), and host–anion (HX) complex (eq 1) into eq 4 and rearranging yields the standard reaction equilibrium expression (see derivation in the SI):
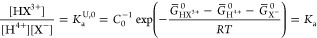
5where *K*_a_^U,0^ is the unscreened equilibrium constant for the host–guest association (eq 3) at the reference state. This unscreened model is the standard 1:1 equilibrium equation that is the basis for the derivation for the nonlinear fitting of spectroscopic or calorimetric data, i.e.,  is *K*_a_, the binding constant typically obtained by supramolecular chemists.^34,35^

When Coulombic screening is considered (κ > 0, i.e., the double layer thickness is finite as described by eq 2), eq 5 can be modified to yield what we refer to as the “screened model” (see derivation in the SI).
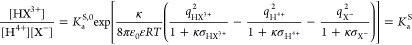
6where *K*_a_^S,0^ is the equilibrium constant of the reference unscreened state (zero salt concentration) and *K*_a_^S^ is the measured affinity at a particular concentration. Note that although *K*_a_^U,0^ (the typical *K*_a_ value supramolecular chemists measure) is independent in the electrolyte concentration in the unscreened model (eq 5), in the screened model (eq 6) *K*_a_^S^ is the product of *K*_a_^S,0^ and a concentration-dependent screening factor. In other words, since κ depends on the salt concentration (eq 2), rather than being a constant, *K*_a_^S^ is a function of concentration.

**Figure 1:**
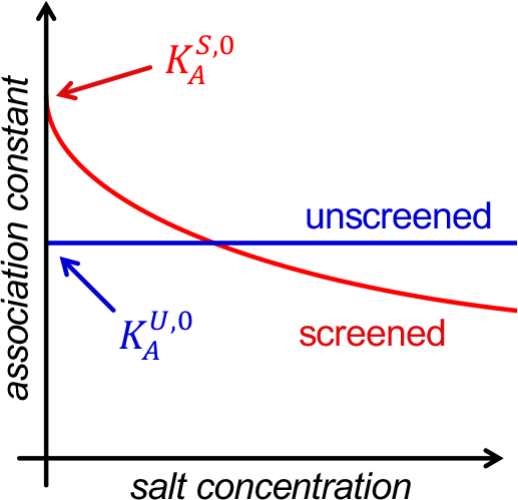
Schematic relationship between *K*_a_^U,0^ and *K*_a_^S,0^ as a function of ionic strength of the solution.

The relationship between the affinity constant values in the unscreened and screened models is schematically shown in Figure 1. Whereas in the unscreened model typically used by supramolecular chemists the affinity is independent of ionic strength (*I*), as the concentration of salt or buffer is increased, the affinity described by the screened model is expected to continuously decrease.^36^ The logical common frame of reference here to compare the two models is at the theoretical situation where *I* is zero, i.e., *K*_a_^U,0^ and *K*_a_^S,0^ for the unscreened and screened model, respectively. These are the values we report below (Table 2).

**Table 1:** Born Radii (σ_*i*_) of the Charged Species Considered in the Charge Screening Model^a^

species *i*	σ_*i*_ (Å)
Host^4+^ (**1**)	6.50
Na^+^	1.94
F^–^	1.48
Cl^–^	2.02
Br^–^	2.12
I^–^	2.36
Host^4+^ (**1**)·F^–^	6.53
Host^4+^ (**1**)·Cl^–^	6.56
Host^4+^ (**1**).Br^–^	6.57
Host^4+^ (**1**)·I^–^	6.60

a A Born radius for host **1** (Host^4+^) of 6.5 Å was assumed. The Born radii of the host–guest complexes were calculated following eq 8.

**Table 2:** Anion Binding Constants and Free Energy Values for Unscreened ( and ) and Screened ( and ) Models Determined from ^1^H NMR Spectroscopy^a^^,^^b^

	unscreened model affinity		screened (Debye–Hückel) model affinity	
anion	*K*_X–_^U,0^ (M^–1^)	*K*_X–_^U,0^ (kJ·mol^–1^)	*K*_X–_^S,0^ (M^–1^)^c^	(kJ·mol^–1^)
F^–^	104 ± 14^d^	–11.49 ± 0.32	61 ± 4	–10.18 ± 0.16
Cl^–^	290 ± 20^e^	–14.06 ± 0.17	452 ± 4	–15.15 ± 0.02
Br^–^	1860 ± 237^d^	–18.64 ± 0.33	2890 ± 65	–19.74 ± 0.06
I^–^	12 800 ± 1450^d^	–23.43 ± 0.29	19 900 ± 1700	–24.52 ± 0.20

a [Host **1**] = at 0.4 mM concentration in unbuffered D_2_O

b The pD values of the solutions were uncorrected.

c Results correspond to a host Born radius of 6.5 Å. Values correspond to Born radii of 5.5 and 7.5 Å were as follows: F^–^, 58 and 64, Cl^–^, 446 and 457, Br^–^, 2880 and 2890, and I^–^, 19 900 and 19 800 M^–1^.

d values for F^–^, Br^–^, and I^–^ obtained by competitive complexation model (eq 14a) using  = 290 M^–1^.

e value obtained by fitting to the standard 1:1 model, accounting for 4 equiv of Cl^–^ and floating the initial point.

To calculate *K*_a_^S,0^, we need more information than is gathered for typical (unscreened model) affinity determinations. Specifically, we must also define the ion-excluding radii, or Born radii (σ) of the ions in solution. Considering that the charge of the host–guest complex  = +3*e*, the charge of the free host **1** is  = +4*e*, and the Born radii of the complexed and free host are expected to be approximately the same (i*.*e., ; see Table 1), it is evident from eq 6 that the calculated screened association constant, *K*_X^–^_^S,0^ is expected to be higher than the unscreened association constant,  (see Figure 1).

To fit the screened, Debye–Hückel model to experimentally obtained data (*vide infra*), the Born radii of the guest anions F^–^, Cl^–^, Br^–^, and I^–^ along with the Na^+^ cation were taken from the literature (fitted to the hydration free energies of the individual ions at infinite dilution).^37^ While host **1** itself is not spherical, the model described above assumes that all the charge species are spheres. Therefore, to treat host **1** as a Born sphere and define its radius (), we evaluated from its crystallographic structure^38^ its radius of gyration (*R*_g_), the effective spherical shell radius that has the same moment of inertia as the host’s actual mass distribution (Supporting Information), and equated it to the Born radius using the following relationship:
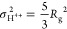
7This expression, derived from the relationship between the radius of a solid sphere and its radius of gyration, gave the Born radius of host **1** of σ = 6.5 Å. Given the assumptions required to map host **1** to a sphere, we tested the robustness of our fitting by assuming Born radii for the host of both 5.5 and 7.5 Å to assess the impact of fitting to the  values. As we describe below, this had a minimal effect. Finally, the Born radius of the host–guest complex was determined by assuming additivity between the Born volumes of the host in the 4+ state and the guest:
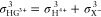
8The Born radii of all the charged species considered here are reported in Table 1.

Having defined the difference between the unscreened model (eq 5) and the screened model (eq 6), as well as the parameters needed for modeling the latter, we now turn our attention to determining the differences between unscreened and screened affinities for halide binding to TMAX-Cl **1**.

### ^1^H NMR Data Collection and Fitting to the Unscreened Model

We first determined the affinity of chloride ion for TMAX-Cl **1**. Here, as above, we assumed the free host to be in the +4 state:^39^
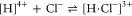
9We began by determining how the counterion influenced halide affinity by carrying out titrations with a series of salts (Li^+^, Na^+^, K^+^, Cs^+^, and Me_4_N^+^, SI, Section 4.A.b). Buffer-free conditions were selected for all initial experiments, a choice consistent with the fact that host **1** contains no ionizable groups, and during multiple titrations the ΔpD was less than ∼0.4 units (lowest and highest pH over all titrations: ∼5.6 and ∼6.8). The ^1^H NMR spectroscopy signals from H_j_ and H_l_ in TMAX-Cl **1** (highlighted in red in Scheme 1) were noted to undergo the largest shifts during titration and were therefore used to report complexation. In these experiments, because there are four equivalents of intrinsic Cl^–^ in TMAX-Cl **1**, the real initial point in the titration corresponding to the theoretical ^1^H NMR signal from the chloride-free host (δ, ppm) is unknown. Therefore, to determine *K*_Cl^–^_^U,0^ to host **1**, the zero-point of the titration was set to correspond to four equivalents of Cl^–^, and the true (theoretical) δ value for the initial point corresponding to zero equivalents of Cl^–^ was allowed to float when fitting (see below). In each titration the host concentration was 0.4 mM in D_2_O. The initial and final ionic strength (*I*) of the solution during this titration was 1.6 and ∼24 mM, respectively.^40^

Data fitting neglecting screening (eq 5) followed standard procedures.^41^ Thus, by using the corresponding mass-balance equations, a quadratic equation for a 1:1 host–guest complexation can be obtained that relates the concentration of free host to the total concentration of host and guest (which can be calculated) and the unknown affinity constant.^35,42^ When this quadratic is itself combined with an equation defining the NMR binding isotherm, eq 10 results:
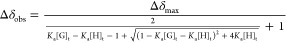
10where Δδ_obs_ is the change in signal shift, Δδ_max_ is the maximum signal shift at the end of the titration, [H]_t_ and [G]_t_ are the total amount of host and guest, and *K*_a_ (= *K*_a_^U,0^) is the affinity constant. In this equation the only unknowns are Δδ_max_ and *K*_a_, and iteratively fitting the experimentally derived binding isotherm to this equation using either the solver in Excel^35,42^ or BINDFIT yields these values.^34^ Note that for the 1:1 binding of anions to host **1** the following assumptions were made:
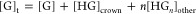
11
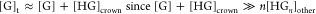
12where [G]_t_ is the total guest concentration, [HG]_crown_ is the concentration of the complex with the anion binding to the crown of four cationic pendent groups of TMAX-Cl **1**, and [HG_*n*_]_other_ is the concentration of complexes arising from nonspecific binding to the host.

All fits to this standard (unscreened) 1:1 model were excellent, with the measured chloride affinities () ranging from 228 to 290 M^–1^ (−13.46 to −14.06 kJ mol^–1^) depending on the counterion (SI, Section 4.A.b/Figures S20–S29). The strongest chloride affinity was observed when the counterion was Na^+^, and the weakest with Li^+^. However, with a range in affinities of only 0.6 kJ mol^–1^ we concluded that the effect of the salt counterion was negligible.

With  to host **1** in hand, we determined the affinity of F^–^, Br^–^, and I^–^ to host **1** by titration with their sodium salts (SI, Section 4.A.c/Figures S31–S36). In each of these titrations the initial ionic strength (*I*) was again 1.6 mM, while the final values were *I* = 56.0, 6.7, and 2.8 mM for the F^–^, Br^–^, and I^–^ titrations, respectively. To determine the affinity constants of these halides (, , and  respectively), we used a standard competitive equilibrium model^43^ in which the host is assumed to be in the 4+ state, but the added halide is in competition with the host binding an intrinsic chloride counterion:
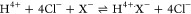
13More specifically, we used a cubic function (eq 14a), which expresses the free host concentration [H] in terms of the total concentrations of the host ([H]_t_), intrinsic chloride ([Cl^–^]_t_), and titrating guest ([X^–^]_t_) as defined by mass balance equations and the affinities of the intrinsic chloride and the titrating guest ( and ).
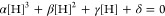
14awhere
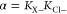
14b
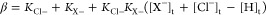
14c
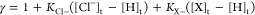
14d
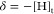
14eSolving eq 14a for the smallest, real, positive root gave [H], which was used in the nonlinear curve fitting of the binding isotherm to determine the binding constants (Table 2).

As anticipated from earlier studies with a larger host possessing an essentially identical crown binding site,^30^ F^–^ bound the weakest, and I^–^ bound with the highest affinity. We attribute the affinity differences in large part to the hydration free energies of each anion. Thus, in the case of iodide, its low hydration free energy means that it can readily shed some of its hydration shell to form a greater number of direct I^–^···Me_3_N^+^R interactions and in doing so partake in not only Coulombic interactions but also C–H···I^–^ hydrogen bonding and van der Waals interactions with the pendent groups of the host. Fluoride on the other hand is too strongly solvated to form direct interactions with the host and, with a strong solvation shell, can only form weak Coulombic interactions. This general concept is supported by the differences in Δδ values in the I^–^ and F^–^ titrations (approximately +0.17 and −0.04 ppm, respectively).

To verify the halide binding data, the tetrabromide salt TMAX-Br **1** was also prepared, and the affinity of Br^–^ () was determined by titrating with NaBr. Again, because there are four equivalents of intrinsic Br^–^ present at the start of the titration, the real initial point corresponding to the theoretical ^1^H NMR signal from the bromide-free host (δ, ppm) was not known. Therefore, to determine , the initial observed point of the titration was set to correspond to four equivalents of bromide, and the true (theoretical) δ value for the initial point corresponding to zero equivalents of Br^–^ was allowed to float when solving for Δδ. This titration gave  = 1890 ± 254 M^–1^ (SI, Section 4.A.c/Figures S37 and S38), in excellent agreement with the data obtained from titration of the tetrachloride salt of **1** with NaBr ( = 1860 ± 237 M^–1^, Table 2). This value for Br^–^ affinity was also used in a competitive complexation model illustrated by eq 14a to determine the affinity of I^–^ toward the tetrabromide salt of **1** (SI, Section 4.A.c/Figures S39 and S40). This gave  = 12 400 ± 1410 M^–1^, again within statistical agreement with the value obtained with the chloride salt ( = 12 800 ± 1450 M^–1^, Table 2). Unfortunately, with the bromide salt of **1** the changes in Δδ_max_ for the signals from H_j_ and H_l_ were too small to accurately determine  and .

### Fitting to the Screened (Debye–Hückel) Model

**Figure 2:**
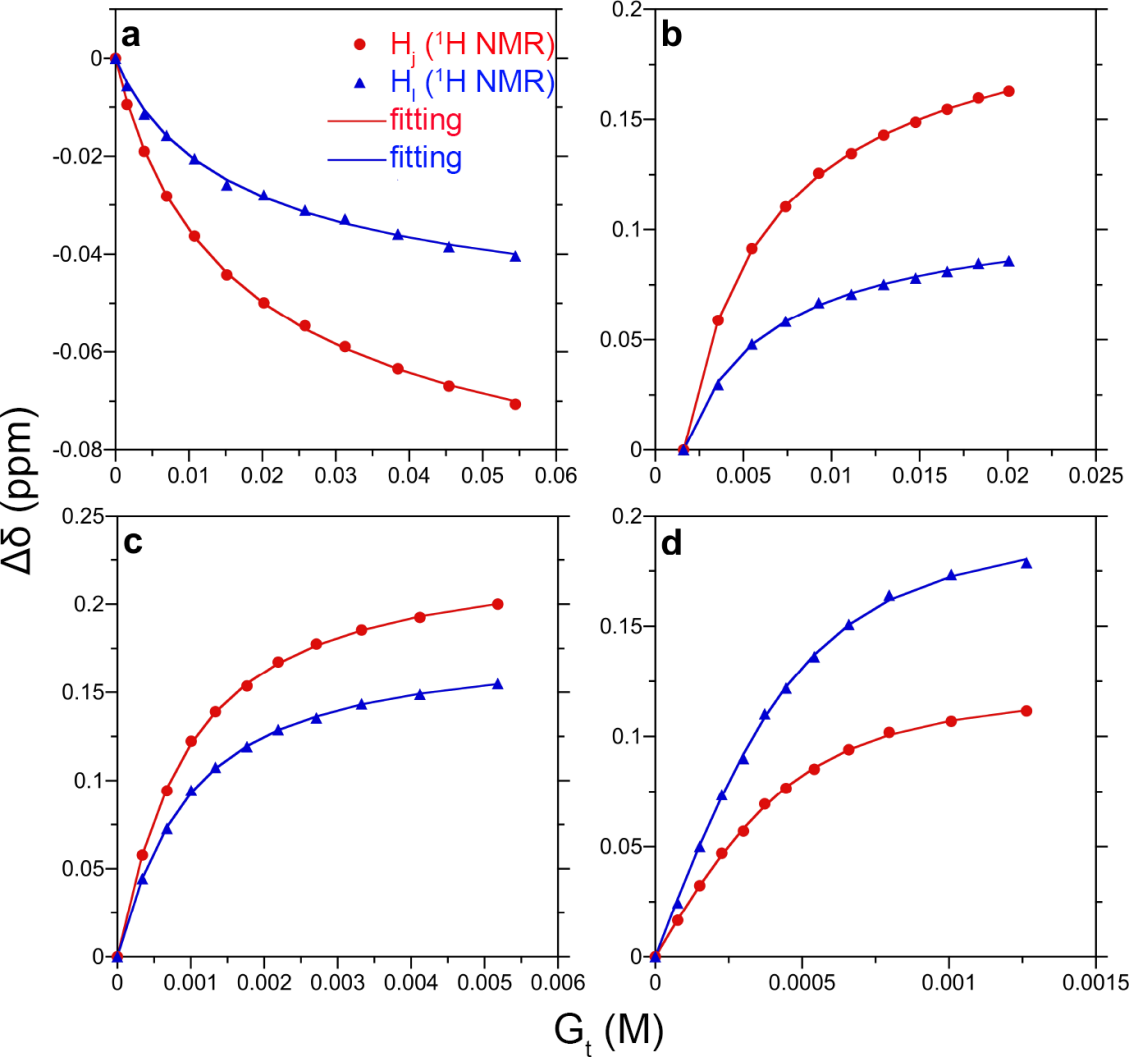
Fits of the Debye–Hückel model (lines) to the ^1^H NMR shift data for H_*j*_ and H_*l*_ signals (points) as a function of the total added guest anion concentration. Results are reported for (a) F^–^, (b) Cl^–^, (c) Br^–^, and (d) I^–^ guests. The figure symbols are defined in the legend in (a).

As an alternative to normal protocols, the same ^1^H NMR shift data for the signals from H_j_ and H_l_ in TMAX-Cl **1** can be treated with a Debye–Hückel model (eq 6) to calculate screened binding constants () for the different halide guests. As summarized above (see Figure 1 and attendant text),  is the obtained affinity from the screened model at a theoretical zero concentration of salt. Specifically, the  values were again determined by performing a global fit to the ^1^H NMR chemical shifts of the signals from H_j_ and H_l_ as a function of host–guest ratio from a representative titration and minimizing the total mean square error (SI, Section 3.B). Given that the inverse Debye screening length κ depends on the equilibrium concentrations of all charged species, the solution of the multiple equilibrium relationships must be determined iteratively. First, we solved the reaction equilibria model for a given set of association constants assuming κ = 0 (i.e., no screening) to generate an initial guess for the equilibrium distribution of host, guest, and host–guest complex(es). Using this estimate in speciation, we evaluated κ and modify the concentration-dependent guest equilibrium constants using eq 6 to obtain an initial  value. The equilibrium concentration distributions were subsequently reevaluated, and the process repeated until the electrolyte concentrations and fitted  values were unchanging. This typically required four to five iterations. As this type of approach is unusual, we show the fitting of the ^1^H NMR data to the screened model in Figure 2.

The set of obtained  values for the screened model are shown in Table 2. The obtained values assuming a smaller (5.5 Å) and larger (7.5 Å) Born radius for the host (Table 2, footnote c) revealed a relative insensitivity to the host size over the range of host Born radii, with the variation in the  values being less than the uncertainty in the fitting. This gives confidence that the model is robust to reasonable perturbations in the effective host radius and that the calculated screened  values are reasonable.

**Figure 3:**
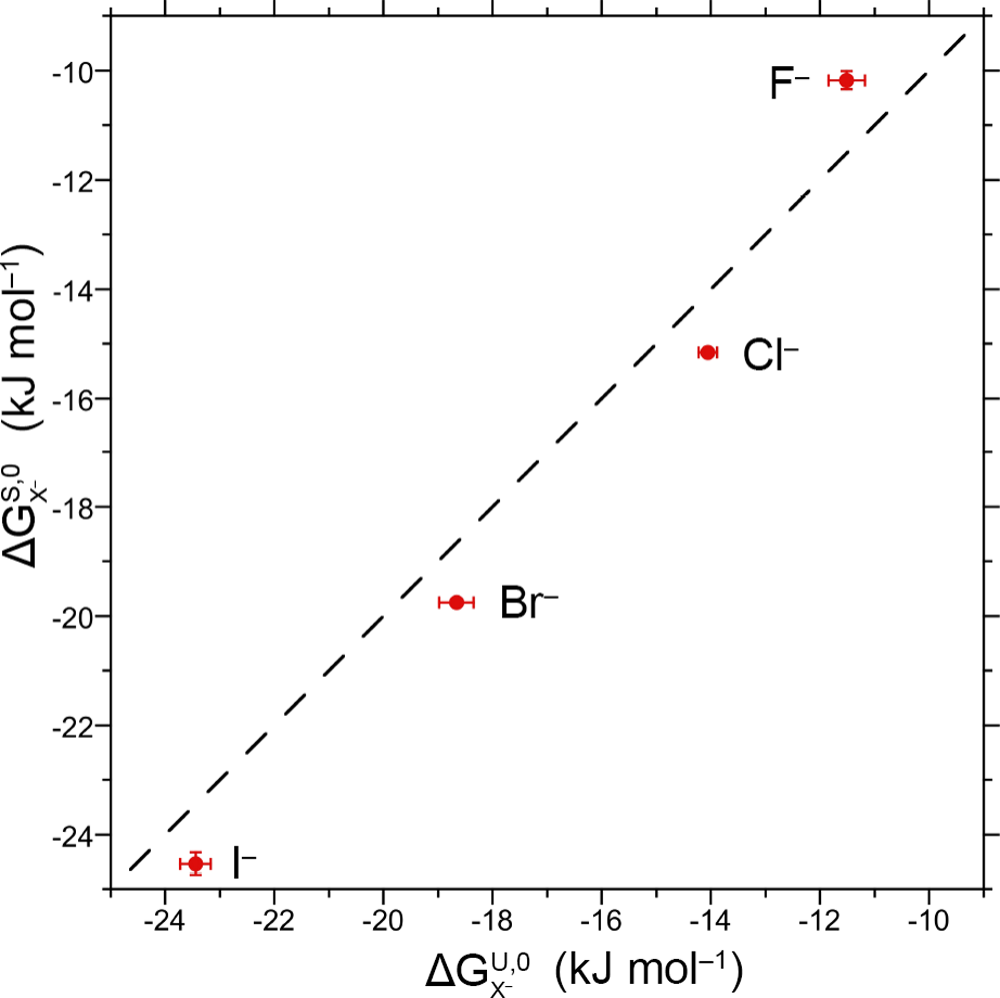
Comparison between the host–anion guest association free energies determined from the unscreened and screened models. The points indicate fit data, while the dashed line indicates perfect agreement. The *x* and *y* error bars indicate one standard deviation.

A comparison of the  and  values in Table 2 reveals that when screening is accounted for with the Debye–Hückel model, the measured affinities are lower for F^–^ but consistently higher for the other halides. We view the calculated F^–^ affinity as anomalous because of its very weak affinity. For the other halides, we observe the larger affinity values expected when using the screened model (Figure 1). Figure 3 compares the corresponding association free energies between **1** and the halide guests for the unscreened () and screened models (); the difference in the free energies is only on the order of 1 kJ mol^–1^ for all the anions, i.e., less than 1/2 of the thermal energy (*RT*) at 25 °C. Thus, from the perspective of Gibb’s free energy, the differences in affinities based on screened and unscreened models are small.

What impact do changes in electrostatic screening during titration have on ? In the case of a representative Br^–^ titration, the concentration of NaBr increased from 0 to 5.2 mM (*I* = 1.5–6.6 mM). Correspondingly, the calculated screened association constant (eq 6) across this range decreased from 1790 M^–1^ to 1470 M^–1^.^44^ The stronger binding I^–^ required a smaller concentration range during a representative titration—from 0–1.3 mM (*I* = 1.8–3.0 mM)—and in this case the calculated screened  decreased only from 12 200 M^–1^ to 11 000 M^–1^. These screened values are in good agreement with those determined from fitting to the normal unscreened model (Table 2). The reason the values do not vary significantly for these two guests is the relatively small salt concentration change during these titrations needed to achieve significant host–guest complexation. However, a considerably wider range of salt concentrations is required to empirically determine the affinity of weaker binding anions Cl^–^ and F^–^. For the former, the salt concentration during a representative titration increases from 0 to 18.6 mM (*I* = 1.6–20.2 mM), and in the screened model this results in a decrease in the  values from 277 M^–1^ to 159 M^–1^. Similarly, using maximal NaF concentrations of ∼54 mM at the end of the representative titration experiment (*I* = 1.6–55.8 mM), the screened model gave an F^–^ affinity () drop from 38 M^–1^ to 13 M^–1^. Given the potential wide variation in the  values for the smaller anions, it is worthwhile to consider the effects of added salt on the distribution of host–guest complexes.

**Figure 4:**
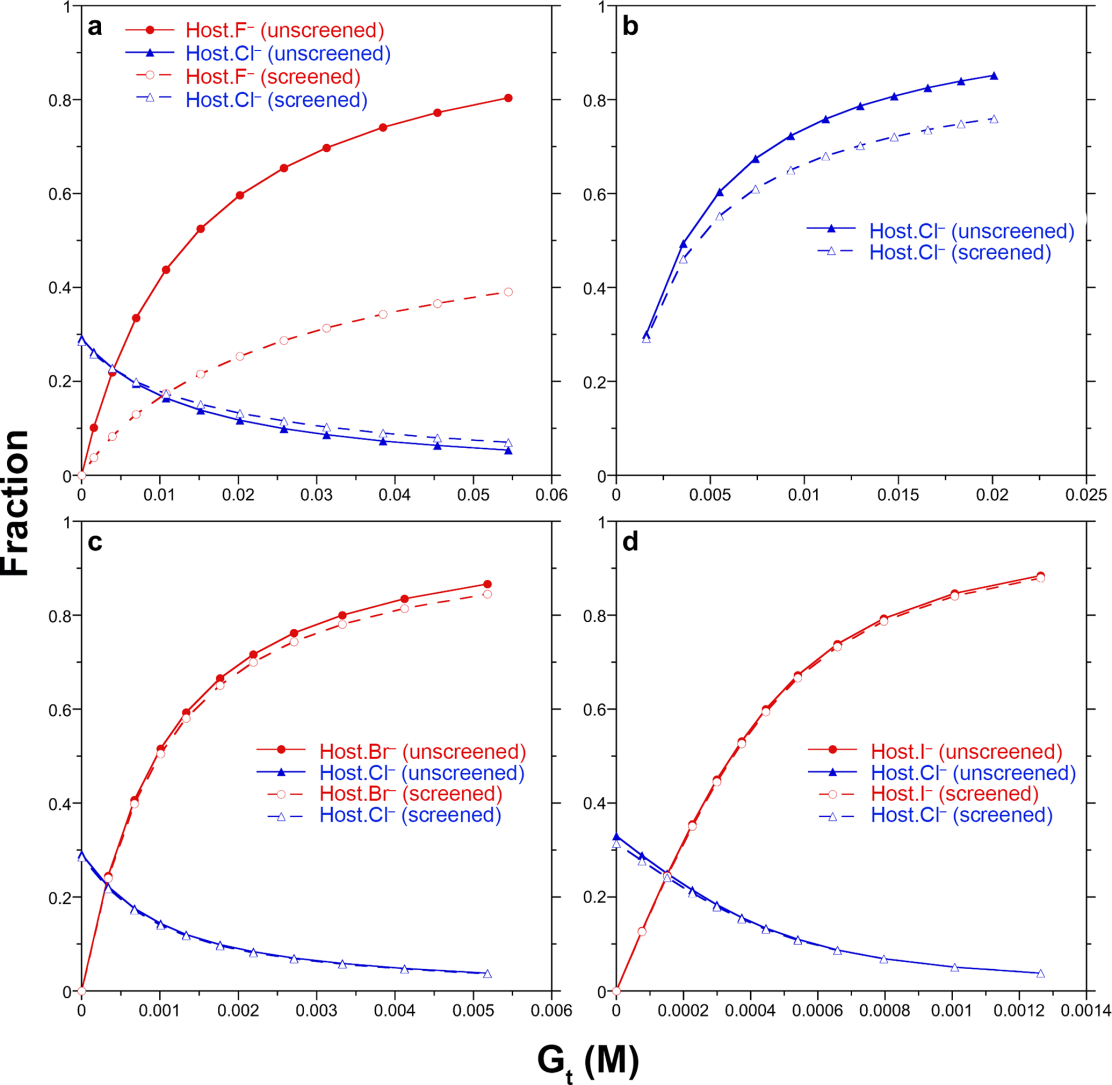
Fraction of the individual host–guest complexes predicted by the unscreened and screened models as a function of the added salt concentration. The fraction of a host–guest complex is defined as the ratio of the concentration of a host–guest complex to the total host concentration (i.e., fraction = [HX]/[H_t_]). Results are reported for the addition of the sodium salts of (a) F^–^, (b) Cl^–^, (c) Br^–^, and (d) F^–^ to host **1** viewed as the tetravalent cation **1**^4+^. The figure symbols are defined in the legends accompanying each figure.

Figure 4 shows a plot of the fraction of host–guest complex for added halide guests predicted by the unscreened and screened models. For these plots we consider the free host **1** to be a tetravalent cation (**1**^4+^). For Br^–^ and I^–^ (Figure 4c and d), the unscreened and screened models effectively predict the same distributions of host and host–guest complex. Thus, over the added salt range the free host population dropped from ∼30% to ∼5%, while the Br^–^ and I^–^ complex populations increase from 0% to 80+%. Larger differences are observed for the NaCl titration (Figure 4b). In general, the screened model consistently underpredicts the fraction of the host–guest complex compared to the unscreened model, an underprediction reflecting the lower magnitude of  at the higher ionic strengths during the latter part of the titration (cf. Figure 1).

The largest difference between the predicted host–guest complex distributions for the unscreened and screened models is observed for the NaF titration system (Figure 4a). While the decreasing fraction of host–chloride complex predicted by the unscreened and screened models closely follow one another, there are large differences between the fractions of fluoride-bound host in the screened and unscreened models. Specifically, we find the screened model predicts a fraction of the fluoride complex that is approximately half that of the unscreened model, despite both models fitting well to the experimental NMR signals shifts (Figure 2a). Why such an underprediction? We believe that the errors in the affinity determination are quite large here because of the small ^1^H NMR signal shifts during the fluoride titration with TMAX-Cl **1** (SI, Figure S32). Specifically, the Δδ_max_ value for the fluoride complex is lower in magnitude than that for the chloride complex by over an order of magnitude. As discussed above, we attribute these very small shifts to the fact that the fluoride ion is strongly solvated and not able to make any direct interactions with the host. Thus, the upfield ^1^H NMR signal shifts observed are likely largely attributable to the weak displacement of chloride from **1**^4+^·4Cl^–^ rather than the formation of the fluoride complex (**1**^4+^·3Cl^–^F^–^). This highlights the limitations of ^1^H NMR spectroscopy as a technique for affinity determinations when the Δδ_max_ values are small.

In summary, for the complexation of anions to **1** in unbuffered solution, a comparison of the standard fitting model with one modified by Debye–Hückel screening reveals sizable affinity constant differences ( versus , Table 2), but that in terms of free energy (because of the logarithmic relationship between free energy and affinity constant), the difference between the two models is small (∼(1/2)*RT* at 25 °C). Our findings do suggest that if ionic strength increases during a titration by more than 1 order of magnitude, then affinity determinations should rely on the Debye–Hückel model. Similarly, if two buffered solutions differ in concentration by an order of magnitude, then screening differences are likely significant and a screening model should be used.

There are other factors that may lie behind differences in measured guest affinities for different buffered systems, including the possibility of competitive binding of buffers. Is this important? And if so, is the effect more significant than any screening effect? We turn to this topic next.

### The Role of Competition

Despite the use of models that do not account for screening, it is commonly observed that association constants are weaker in buffered versus unbuffered solutions. What is the cause of this phenomenon? We surmised that host–buffer binding (and hence competition with the analyte guest) is likely key, and with the halide affinities in unbuffered solutions in hand, we turned to the effects of biologically relevant phosphate buffers on these binding constants. In this set of experiments, we opted to use the standard model for data fitting that neglect screening to ascertain the magnitude of any such buffer competition effect relative to the differences arising from screened versus unscreened models.

For reasons described below, we did not investigate halide binding at high pH values where trivalent phosphate (PO_4_^3–^) dominates the speciation graph for phosphate buffer (SI, Section 4.A.d/Figure S41). Rather, the focus was on buffered solution in the slightly basic to acidic range. As a first step, we used titration experiments to determine the affinity of dihydrogen phosphate (H_2_PO_4_^–^) and hydrogen phosphate (HPO_4_^2–^) to TMAX-Cl **1**. These experiments were possible because in both cases the change in speciation over the pH change during titration were not significant. Consider first the titration with the sodium salt of HPO_4_^2–^. Solutions of HPO_4_^2–^ inevitably contain varying amounts of H_2_PO_4_^–^ and HO^–^ from the reaction of HPO_4_^2–^ with water, but during the host–guest titration to determine HPO_4_^2–^ affinity, the pD varied only from ∼8.4 (after the first aliquot of salt) to ∼9.6 at the end of the titration. Thus, over the titration the concentration of HO^–^ ranged from ∼0.003 to 0.04 mM (<0.01–0.1 mol %), and the mole percent of HPO_4_^2–^ varied from 94% to >99%. This relatively small change in speciation allowed the ^1^H NMR data to fit a competitive model (eq 14a) where only HPO_4_^2–^ was assumed to be in competition with the intrinsic Cl^–^ for host **1**. In this titration *I* ranged from 1.6 mM at the initial point to 51.3 mM at the end. The corresponding titration with H_2_PO_4_^–^ involved a smaller change in buffer speciation. Here, the pH varied from ∼5.5 to ∼4.6, and thus the mole percentage of H_2_PO_4_^–^ was always >98% and the change in hydronium ion concentration negligible (0.003–0.03 mM). As a result, the data fitted a normal competitive complexation model where only H_2_PO_4_^–^ was in competition with the intrinsic Cl^–^ of TMAX-Cl **1**. In this titration the change in *I* was similar to that of the titration with HPO_4_^2–^, ranging from 1.6 to 41.4 mM.

In contrast, the affinity of trivalent phosphate (PO_4_^3–^) could not be investigated because the major species at strongly basic conditions are hydrogen phosphate (HPO_4_^2–^) and HO^–^, and having four major species in solution (host **1**, Cl^–^, HPO_4_^2–^, and HO^–^) precluded application of the competitive model (eq 14a). This point aside, the calculated affinity for H_2_PO_4_^–^ () was found to be 72 ± 7 M^–1^ (Δ*G*^U,0^= −10.60 ± 0.22 kJ mol^–1^), while divalent HPO_4_^2–^ bound slightly more strongly ( = 302 ± 31 M^–1^, Δ*G*^U,0^ = −14.16 ± 0.23 kJ mol^–1^). Both buffer species are relatively weak binders, with H_2_PO_4_^–^ binding slightly weaker than F^–^, and HPO_4_^2–^ binding slightly more strongly than Cl^–^. Interestingly, the higher affinity of HPO_4_^2–^ over H_2_PO_4_^–^ exists despite its much higher free energy of hydration (−1089 versus −473 kJ mol^–1^, respectively). This point gave us confidence that any trace amounts of neutral trihydrogen phosphate (H_3_PO_4_) present in these experiments did not associate with host **1**.

With these affinity constants in hand, we sought to determine if the buffers attenuated the affinity of the halide guests. We therefore carried out halide ion affinity determinations using TMAX-Cl **1** in three different buffered solutions (SI, Section 4.A.e/Figures S46 and S47). The conditions selected were as follows: (1) a 10 mM buffered solution of pH = 7.3 (45% H_2_PO_4_^–^, 55% HPO_4_^2–^, ionic strength = 21.0 mM (22.6 mM including the host)); (2) a 23.8 mM pH = 3.0 buffer of the same ionic strength (12% H_3_PO_4_, 88% H_2_PO_4_^–^, *I* = 21.0 mM (22.6 mM including the host)); and (3) a pH = 3 solution at lower ionic strength (12% H_3_PO_4_, 88% H_2_PO_4_^–^, *I* = 8.8 mM (10.4 mM including the host)). The final *I* values for each titration are shown in Table S4 but were maximal at pH = 7.3 and calculated to be 94.0, 57.2, 29.9, and 25.5 mM in the case of the F^–^, Cl^–^, Br^–^, and I^–^ titrations, respectively.

The observed binding constants (*K*_obs_^U,0^) for each halide are reported in Table 3. In the case of Cl^–^, the affinity constant was attained by fitting the data to a 1:1 model. In contrast, for the other halides the titration data were fitted to a competitive model (eq 14a) in which the halide guest was in competition with the four intrinsic Cl^–^ ions of host **1**. For these latter calculations the binding constant of Cl^–^ used was the *K*_obs_^U,0^ value for chloride under each of the buffered conditions, i.e., 135, 143, and 166 M^–1^ for respectively 10.0 mM phosphate at pH 7.3, 23.8 mM phosphate at pH 3.0, and 10.0 mM phosphate at pH 3.0 (Table 3).

**Table 3:** Observed (*K*_obs_^U,0^) and Predicted (*K*_pred_^U,0^) Binding Constants for the Binding of Halide Guests to **1**^a^

	*K*_obs_^U,0^ (M^–1^)^b^			*K*_pred_^U,0^ (M^–1^)^c^		
guest	10 mM, pH = 7.3^d^	23.8 mM, pH = 3.0^e^	10 mM, pH = 3.0^f^	10 mM, pH = 7.3^d^	23.8 mM, pH = 3.0^e^	10 mM, pH = 3.0^f^
F^–^	—g	—g	—g	35 ± 7	40 ± 8	62 ± 12
Cl^–^	135 ± 3	143 ± 8	166 ± 6	120 ± 20	130 ± 20	180 ± 20
Br^–^	738 ± 27	862 ± 48	1020 ± 51	630 ± 130	740 ± 150	1110 ± 200
I^–^	5430 ± 324	6000 ± 489	7410 ± 314	4325 ± 810	5110 ± 810	7850 ± 1240

a [Host 1] = 0.4 mM.

b Average values based on at least three determinations.

c Errors were propagated from the relative errors of each of the anions (SI Section 4.A.f).

d 10 mM sodium phosphate buffer, pH 7.3 (I = 21.0 mM).

e 23.8 mM sodium phosphate buffer, pH 3.0 (I = 21.0 mM).

f 10 mM sodium phosphate buffer, pH = 3.0 (*I* = 8.8 mM).

g The measured binding was too weak to determine accurately.

As expected, the presence of buffer lowered the affinity constants (cf. Table 2), and the higher the pH value or the higher the ionic strength, the greater this attenuation. This is consistent with the idea that at pH = 7.3 there is a slight excess of more strongly binding HPO_4_^2–^ over H_2_PO_4_^–^ and that both are in competition with halide ion for the pocket of **1**, whereas at pH = 3.0 the only significant competitor for the pocket of **1** is weakly associating H_2_PO_4_^–^. The fact that no affinity for F^–^ could be measured under buffered conditions is unsurprising considering its weak association relative to HPO_4_^2–^ and its comparable affinity to H_2_PO_4_^–^. Are these attenuations caused by competitive ion binding? To address this question, we built a mathematical model to predict the affinity of the halide ions based only on competition processes with other anions in solution.

Determining association constants in a straightforward competition system, for example one involving a halide and a single-component buffer and the host, involves a cubic equation (eq 14a). However, it is more complex if three or more guests are involved and their individual binding constants are unknown. For example, the *de novo* determination of  to host **1** in a two-component buffer or the *de novo* determination of  to the (chloride salt) of host **1** in the presence of a one-component buffer requires the solution of a quartic equation, and for each additional binding species in the system a correspondingly higher polynomial is required. In these systems, it is not usually possible to determine all the association constant values in question *de novo*. In contrast, when association constants in the absence of additional species are known for all the requisite guests (in this case the halides and mono- and divalent phosphates), it is possible to use these to predict an unknown guest affinity in a complex (buffered) mixture using a simulated titration based on a mathematical model involving multiple competitive binding processes. Here, we define such predicted association constants in buffer as *K*_pred_^U,0^.

To illustrate the prediction of affinity constants in a buffered system (*K*_pred_^U,0^), consider the titration of halide X^–^ (F^–^, Br^–^, or I^–^) into a solution containing TMAX-Cl **1** in phosphate buffer (pH = 7.3). Let Y^*–*^ and Z^2*–*^ correspond to the acid (H_2_PO_4_^–^) and conjugate base (HPO_4_^2–^) portions of the buffer. The respective concentrations of the free species and those of the host–acid and host–conjugate-base complexes are [Y^*–*^], [Z^2*–*^], [HY^3*+*^], and [HZ^2*+*^], and the mass balance for the free host concentration is [H^4+^] = [H^4+^]_t_ – [HCl^3+^] – [HX^3+^] – [HY^3+^] – [HZ^2+^]. Since [HG] = *K*_guest_[H][G], and there are equivalent expressions for [HCl^3+^], [HX^3+^], [HY^3+^], and [HZ^2+^], an expression can be derived (SI, Section 4.A.f, Appendix B) for the free host concentration:
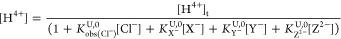
15In eq 15, [H^4+^]_t_ is known, as are each of the previously determined anion affinities: *K*_obs_^U,0^ for chloride under the buffered conditions and , , and  (again, neutral H_3_PO_4_ was assumed not to bind to **1**). What is unknown are the free (unbound) guest concentrations. It can be shown that for general guests [G]_t_ = [HG] + [G] (SI, Section 4.A.f, Appendix B) and that there are equivalent expressions for [Cl^–^]_t_, [X^–^]_t_, [Y^–^]_t_, and [Z^2–^]_t_. Since, in general terms [G]_t_ = *K*_G_[H][G] + [G], an expression can be derived for the free guest concentration, [G], for any guest in terms of the total guest concentration [G]_t_, its association constant *K*_G_, and the free host concentration ([H]):
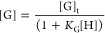
16

Equations 15 and 16 can be solved iteratively. Thus, eq 16 can be used to solve for the concentration of each free species ([Cl^–^], [X^–^], [Y^–^], and [Z^2–^]) after substitution of the appropriate term for the guest, G, with Cl^–^, X^–^, Y^*–*^, or Z^2–^ respectively, and these solutions can be used to solve eq 15, the solution of which, [H], in turn is used to solve eq 16 for each guest. In each case the total concentration of each guest is known. Importantly, [Y^–^]_t_ and [Z^2–^]_t_ remain fixed (excess of buffer), and [Cl^–^]_t_ (=4 × [H]_t_) decreases in a known manner during the simulated titration as buffered guest solution is incrementally added. In each calculation the iterative process was carried out until the maximal change was <0.0001.

Once the concentrations of each free and bound species had been ascertained, the data were used to construct a speciation diagram. Subsequently, the Δδ_max_ values for each species obtained from their individual titrations were used to construct wholly artificial NMR spectroscopy-based binding isotherms, and from these simulated ^1^H NMR data, the sought binding constant was calculated in the usual manner. Table 3 shows these *K*_pred_^U,0^ data for the same three sets of conditions used for the *K*_obs_^U,0^ data. Overall, the many terms necessary for determining *K*_pred_^U,0^ resulted in relatively large errors because the individual affinity errors are propagated in eq 15. For example, the calculation involving the chloride salt of **1** in 10 mM phosphate buffer, pH 7.3, with Br^–^ as the titrant has associated errors of Cl^–^ (7%), HPO_4_^2–^ (9%), H_2_PO_4_^–^ (10%), and Br^–^ (13%). Correspondingly, the propagated error for *K*_pred_^U,0^ in this system is ∼20% (SI, Section 4.A.f). This noted, a comparison of the *K*_obs_^U,0^ and *K*_pred_^U,0^ data (Table 3) reveals that they are within error, suggesting that the observed affinity attenuations in buffered solutions arise from direct competitive binding to the host by the buffering species.

How does the influence of screening compare to the effects induced by guest competition? Obviously, an answer to this question is context dependent and will depend on both factors, but in the system at hand, the change in Δ*G°* induced by buffer binding is approximately twice that observed by screening. For example, the Δ*G°* of binding Cl^–^, Br^–^, and I^–^ to TMAX-Cl **1** in 10 mM phosphate buffer (pH = 7.3) are respectively 12.2, 16.3, and 21.3 kJ mol^–1^. If these values are compared to those obtained in the absence of buffer (Table 2), it is apparent that the decrease due to buffer competition is 1.8 to 2.3 kJ mol^–1^, i.e., close to *RT* (2.48 kJ mol^–1^ at 25 °C). In contrast, accounting for screening results in a change in Δ*G°* approximately half this amount.

## Summary and Concluding Remarks

Using the standard unscreened model for affinity determinations, we have measured the affinity of halide ions and the buffer species H_2_PO_4_^–^ and HPO_4_^2–^ to TMAX-Cl **1** (). Additionally, for the halide guests we have calculated their affinities using a Debye–Hückel model that accounts for the effects of screening. We find that affinity determinations of weak guests are significantly different in screened versus unscreened models and that, as a rule of thumb, if the ionic strength between two solutions or between the beginning and end of a titration differs by more than 1 order of magnitude, a screening model should be used to determine affinity.

Additionally, we have determined the affinity of halides for TMAX-Cl **1** in three buffered solutions. As is commonly observed with host–guest complexations in aqueous solution, we have shown that halide ion affinities () to TMAX-Cl **1** are attenuated in the presence of the phosphate buffer (*K*_obs_^U,0^). A complexation model for affinity predictions based only on competitive guest complexation (*K*_pred_^U,0^) reveals that this attenuation can be accounted for by buffer binding.

Overall, we find that the effects of buffer competition on anion affinity to be approximately double the effect of screening. Thus, for strong binding guests such as I^–^ it is less important to account for screening than it is to account for competitive buffer complexation. However, for weak binding host–guest systems both screening and competitive binding should be considered. These guidelines are obviously just that, guidelines. Depending on what application a supramolecular chemist is considering and depending on the system under study, it may or may not be important to consider screening. In Figure 4b, the difference between the estimated amount of complexed chloride using a screened or unscreened model may or may not be important in, for example, the development of new extraction protocols. That noted, we do hope that the results described here give supramolecular chemists a frame of reference or calibration point by which to evaluate their own particular system.^45^

More generally, the fact that buffers can bind to TMAX-Cl **1** highlights the dangers of using buffers indiscriminately. Although many design principles^18^ went into Good’s buffers, the minimization of supramolecular interactions (beyond metal coordination) was not one of them.^12,13^ Within the list of common (heritage) buffers, the different phosphate species are relatively strongly solvated (Δ*G*_hydr_ of PO_4_^3–^, HPO_4_^2–^, and H_2_PO_4_^–^ respectively −2773, −1089, and −473 kJ mol^–1^).^11^ As a result, we surmise that PO_4_^3–^, HPO_4_^2–^, and H_2_PO_4_^–^ are not likely to interfere with the binding of a nonpolar guest to a nonpolar pocket; the hydrophobic effect is quite orthogonal to the Coulombic interactions dominating any supramolecular properties of the three phosphate species. However, the effects of HPO_4_^2–^ and H_2_PO_4_^–^ binding to the charged site of TMAX-Cl **1** are apparent; even a strongly hydrophilic buffer such as phosphate can have a significant effect on guest affinity when the host site is charged. Despite this, we would argue that small, strongly solvated inorganic species such as the three phosphates, sulfate (Δ*G*_hydr_ −1090 kJ mol^–1^), carbonate (−479 kJ mol^–1^), hydrogen carbonate (−368 kJ mol^–1^), and acetate (−373 kJ mol^–1^) can function as excellent buffers; they are all more strongly solvated than chloride (−347 kJ mol^–1^). That noted, other factors must also be considered. For example, depending on the experiment, phosphate may be an entirely inappropriate buffer for the study of ATPases. Equally, carbonate/hydrogen carbonate buffer would be inappropriate for the study of the carbonic anhydrase family. While users need to be cognizant of such occasional incompatibilities of these buffers, our studies here should alert users to common organic buffers that undoubtedly—and likely generally—interfere. In our estimation, heritage buffers such as piperazine-based HEPES, morpholine-based MOPS, or TRIS should never be assumed to be spectator species.^18^ They simply possess nonpolar surfaces that are too extensive and/or functional groups that are known supramolecular motifs. There is still much to learn here. However, appreciating the supramolecular portfolio of each buffer should allow the creation of a comprehensive compendium of ideal buffers and their strengths and limitations, which undoubtedly will be of utility to a great many users.

## Supporting Information Available

Details of materials, instrumentation and sample preparation procedures, synthesis and characterization data, derivations of unscreened and screened (Debye–Hückel) models, and analytical data (PDF)
Spreadsheet utilized in the modeling (XLSX)
Spreadsheet utilized in the modeling (XLSX)
