# Supplementary material for: Buffer and Salt Effects in Aqueous Host–Guest Systems: Screening, Competitive Binding, or Both?
Source: J Am Chem Soc. 2021 Oct 27;143(44):18605–16. doi: 10.1021/jacs.1c08457 (PMC8587612; doi:10.1021/jacs.1c08457)
Supplement: Supplementary file 1 — ja1c08457_si_001.pdf [file ja1c08457_si_001.pdf]

Supporting Information for

Buffer and salt effects in aqueous host-guest systems: screening, competitive binding, or both?

Jacobs H. Jordan,[1] Henry S. Ashbaugh,*[2] Joel T. Mague[3], and Bruce C. Gibb*[3]

Jacobs H. Jordan https://orcid.org/0000-0002-0238-3864
Henry S. Ashbaugh https://orcid.org/0000-0001-9869-1900
Bruce C. Gibb https://orcid.org/0000-0002-4478-4084

[1] US Department of Agriculture, Agricultural Research Service Southern Regional Research Center, 1100 Robert E. Lee Blvd, New Orleans, LA, USA 70124
[2]Department of Chemical and Biomolecular Engineering, Tulane University, New Orleans, LA 70118
[3] Department of Chemistry, Tulane University, New Orleans, LA 70118, USA

*Corresponding Authors: bgibb@tulane.edu and hanka@tulane.edu

Table of Contents

1. Materials, instrumentation and sample preparation procedures S3
   A. Materials and instrumentation S3
   B. Electron Spray Ionization (ESI) MS sample procedure S3
   C. Nuclear Magnetic Resonance (NMR) Spectroscopy solution preparation procedure S3
   D. Matrix Assisted Laser Desorption Time-of-Flight (MALDI-TOF) sample procedure S3
   E. Errors S4
2. Synthesis and Characterization Data S5
   A. Synthesis of host **1** S6
      a. Synthesis of *tetra*-halide cavitand **3** S6
      b. *Synthesis of tetrakis*-(trimethylammonium) cavitand **1** S6
   B. Spectral Characterization Data S7
3. Derivation of unscreened and screened (Debye-Hückel) Models S14
   A. Derivation of screened reaction mode S14
   B. Fitting of screened reaction model to experimental data S16
4. Analytical Data S16
   A. NMR Titration data S16
      a. Determine the affinity of chloride to cavitand **1** S16
      b. Determining the effect of the cations on chloride binding S22
      c. Other halide affinity determinations to cavitand **1** S27
      d. Buffer complexation determinations S34
      e. Determination of binding constants in buffered systems S37
      f. Predicting binding constants in complex mixtures S37
5. References S44
6. Appendix A S44
7. Appendix B S45
8. Appendix C Screened binding worksheet description S47

1. Materials, instrumentation and sample preparation procedures

A. Materials and Instrumentation

Reagents were purchased from commercial suppliers Sigma-Aldrich Corp. or TCI America and were used without further purification. All sodium salts were of purity ≥ 98 %, and were used as received. All solvents were purchased from Fisher Scientific and were used as received. All synthetic procedures utilized a nitrogen atmosphere which was maintained by the addition of a nitrogen-filled balloon. Resorcinarene **2,** was synthesized by the procedure recently reported.[1] Cavitand **3** was synthesized by modifications to a scaled procedure reported previously for propanol-footed cavitands.[2,3] All ^{1}H NMR spectra were collected on a Bruker 500 MHz, Bruker 300 MHz, or Varian 400 MHz spectrometer at 25 ˚C, and all ^{13}C NMR spectra were collected on a Bruker 300 MHz (75 MHz ^{13}C) at 25 ˚C. All titrations utilized deuterium oxide (Cambridge Isotopes, 99.9%+) and Δδ are referenced to the residual solvent signal (δ = 4.70 ppm). Spectral processing was performed using Mnova software (Mestrelab Research, S.L.) with results fitted using the online software BINDFIT[4] or SOLVER in Microsoft Excel. MALDI-MS and ESI-MS spectra were collected using a Bruker Autoflex II MALDI-TOF mass spectrometer and a Bruker microTOF mass spectrometer, respectively.

B. Electron Spray Ionization (ESI) MS sample procedure

All samples were prepared as 20 μM concentration solutions in distilled H_2O (dH_2O). ESI-MS spectra acquisitions were acquired using a Bruker microTOF mass spectrometer in positive mode and generally averaged from 1.0–10.0 minutes. Ions were continuously generated by infusing the aqueous solution samples into the source with a syringe pump at flow rates of 6 μL/min. The parameters were adjusted and were typically as follows: capillary voltage (–4.1 kV); capillary exit voltage (70 V); skimmer voltage (40 V); drying gas temperature (200 °C). The experiments were carried out with a nebulizer gas pressure of 0.3 Bar and a drying gas flow of 4.0 L/min.

C. Nuclear Magnetic Resonance (NMR) Spectroscopy solution preparation procedure

All solutions were prepared in unbuffered D_2O or phosphate buffered D_2O as described in the individual sections. All titrations of host were carried out with ~0.4 mM host solutions prepared from a concentrated stock of ~2 mM; the concentration of the stock solution was determined by titration in triplicate with separate 25 mM sodium ethanesulfonate (SES) solutions, and integration of the methyl peak of ethanesulfonate and the H_m and H_l peak of the host. A concentrated salt solution between 10-500 mM was prepared for use in each titration. The pD of the solutions was uncorrected for titrations in unbuffered D_2O and adjusted, if necessary, during dilution for titrations using phosphate buffer. For ^{1}H NMR spectroscopy titration experiments, 0.5 mL of host solution in an NMR tube was careful titrated with small aliquots of the corresponding sodium salt of the anionic guest.

D. Matrix Assisted Laser Desorption Time-of-Flight (MALDI-TOF) sample procedure

Solutions of the host were prepared by the dried-droplet method. Thus, a solution of *tetra*-halide cavitand **3** (~2.0 mM in $CHCl_3$) was used to prepare 2.5 μL droplets containing a ratio of matrix-analyte-salt of 2:1:1. The matrix used was *trans*-2-[3-(4-*tert*-butylphenyl)-2-methyl-2-propenylidene]malononitrile (DCTB, 10 mg•mL^{-1} in acetone). Sodium trifluoroacetate (1 mg•mL^{-1} in acetone) was used as the cation source and the analyte detected in positive mode.

E. Errors

Results are expressed as the average, when possible, with the coefficient of variation (CV) expressed as a percentage of the mean when applicable, where *s* is the sample standard deviation and *μ* is the sample mean i.e.: $CV\% = \frac{s}{\mu} \times 100$

2. Synthesis & Characterization Data

A. Synthesis of host 1

Scheme S1. General scheme for the synthesis of water-soluble anion receptor **1**.

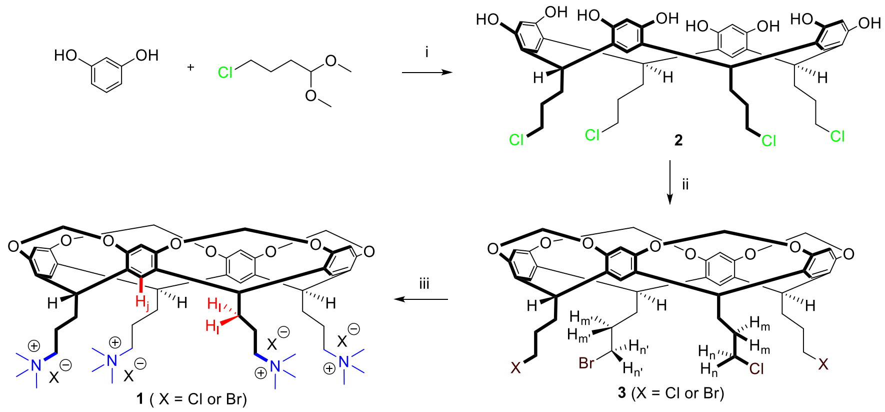

Reagents and conditions: i) HCl / MeOH (10:3), 0 °C, 30 min, then 55 °C 5 d; ii) K_2CO_3, DMA, CH_2BrCl, 55 °C, 7 d; iii) $N(CH_3)_3$, DMF/H_2O (10:1), 70 °C, 3 d.

The general synthetic scheme for the synthesis of receptor **1** is shown in Scheme S1. The synthesis and characterization of resorcinarene **2** was reported previously.[1] Subsequent bridging of the phenolic oxygens gives *tetra*-halide **3** (X = Cl & Br) in ~20 % yield. The yield for this reaction was calculated assuming formation of the dibromo-dichloro cavitand. For this product, ^{1}H NMR and COSY NMR spectroscopy (Figure S1 & Figure S2, respectively) revealed two sets of methylene resonances corresponding to the situation whereby the feet/pendant groups are partially brominated or chlorinated. The substitution by bromide is the result of the utilization of bromochloromethane for bridging. Addition of NaBr to the bridging reaction resulted in increased amounts of brominated product, but irrespective of this modification separation of the different chlorinated/brominated products was not possible. The carbon atoms attached to the chlorine or bromine functionality as well as the adjacent carbons could be readily distinguished by ^{13}C NMR spectroscopy (Figure S3), while MALDI-TOF MS (Figure S4 & Figure S5) analysis of a sample shows the presence of one (m/z = 884), two (m/z = 928), three (m/z = 972), or four (m/z = 1016) bromine atoms in the structure. Integration of the peaks in the ^{1}H NMR spectrum (Figure S6) associated with the methylenes one or two bonds adjacent to the halogen substituents revealed there are 3.4 ± 0.1 hydrogens corresponding to a chlorinated terminal atom and 4.6 ± 0.1 hydrogens corresponding to a bromine as the terminal atom. This effectively represents the case whereby there are 1.60 – 1.75 chlorine atoms per molecule and 2.25 – 2.40 bromines. These numbers were supported by elemental analysis.

The synthesis of water-soluble receptor **1** (X = Cl^- or Br^-) utilized a Menshutkin reaction with halide **3** followed by ion exchange to the chloride (Cl^-) or bromide (Br^-) salt with Dowex anion exchange resin. Each salt was further purified using Biogel-P2 size-exclusion media. The final product was obtained from the three linear steps in ~12 % overall yield.

a. Synthesis of tetra-halide cavitand **3**

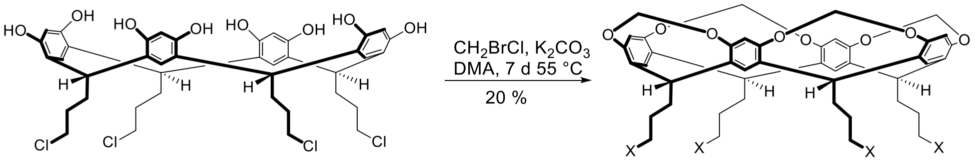

Resorcinarene **2** 10.3 g (13 mmol) was added to an oven-dried 1000 mL round-bottomed flask equipped with a magnetic stir bar. To this was added (oven-dried) potassium carbonate (16.1 g, 9.0 mmol) and DMA (300 mL). The solution was degassed for several min. until all vigorous bubbling had ceased. Bromochloromethane (40 mL, 616 mmol) was added and the reaction heated to 55 °C (oil bath temp) for 7 d. Afterwards, the reaction was removed from the heat source, cooled to rt, and the solvent removed under reduced pressure. The resulting solid were suspended in 1 M HCl (200 mL), collected by filtration, washed with water (3 x 200 mL), and then dried overnight at 110 °C to give a crude off-white solid. The solid was then purified by chromatography (dry-load 100 % DCM) to give the desired product as a white solid (2.73 g, 20 % calcd. as the dibromide). ^{1}H NMR (300 MHz, $CDCl_3$) δ 7.19 (s, 4H), 6.53 (s, 4H), 5.77 (d, *J* = 7.2 Hz, 4H), 4.80 (t, *J* = 8.1 Hz, 4H), 4.46 (d, *J* = 7.2 Hz, 4H), 3.71 (t, *J* = 6.1 Hz, 4H), 3.58 (t, *J* = 6.1 Hz, 4H), 2.65 – 2.27 (m, 8H), 2.08 – 1.93 (m, 4H), 1.93 – 1.81 (m, 4H). ^{13}C NMR (75 MHz, $CDCl_3$) δ 155.01, 138.02, 120.39, 116.78, 99.48, 77.42, 77.00, 76.57, 45.08, 35.90, 35.79, 33.97, 33.90, 33.83, 31.05, 30.95, 28.32, 27.07. *MALDI-TOF MS* m/z: $[M–e^-]^+$ Calcd. for $C_{40}H_{44}BrCl_3O_8$ 884.13; Found 884.01; Calcd for $C_{40}H_{44}Br_2Cl_2O_8$ 928.08; Found 927.95; Calcd. for $C_{40}H_{44}Br_3ClO_8$ 972.03; Found 971.90; Calcd for $C_{40}H_{44}Br_4O_8$ 1015.98; Found 1015.92. Anal. Calcd. for $C_{40}H_{44}Br_{2.4}Cl_{1.6}O_8$: C, 55.67; H, 4.67. Found: C, 55.71; H, 4.69.

b. Synthesis of *tetrakis*-(trimethylammonium) cavitand **1**

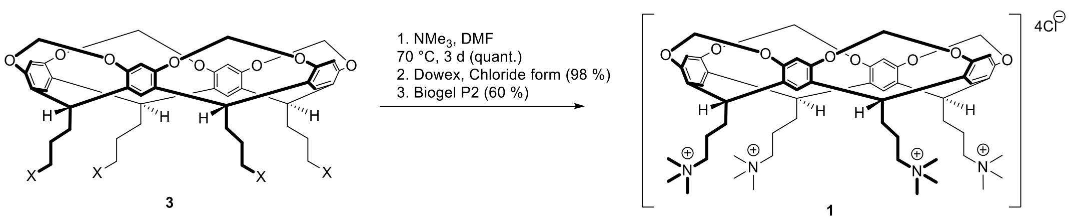

190 mg (0.2 mmol) of **3** was added to a dried 50 mL RBF. To this was added 33 mL DMF-dH_2O (10:1) and 1.0 mL (4.1 mmol, 20 equiv.) of trimethylamine (33 w % solution in EtOH). The solution was heated to 70 °C for 3 d. After this time the reaction was cooled to rt, the solvent removed under reduced pressure, and the resulting solid dried under high vacuum for 6 h. at rt. The crude solid was then suspended in dichloromethane and washed with dichloromethane (3 x 20 mL) to collect a pale solid powder which was dried overnight under high vacuum and at 77 °C. The solid was subsequently dissolved in minimal dH_2O and passed through an anion exchange column (Dowex anion exchange resin chloride form), and then a P2 size-exclusion column, to give pure **1** as a fluffy white solid after lyophilization (133 mg, 60 %). ^{1}H NMR (400 MHz, D_2O) δ 7.48 (s, 4H), 6.54 (s, 4H), 5.65 (d, *J* = 7.7 Hz, 4H), 4.63 (s, 4H), 4.56 (t, *J* = 7.6 Hz 4H), 4.18 (d, *J* = 7.7 Hz, 4H), 3.37 – 3.28 (m, 8H), 2.89 (s, 36H), 2.42 (q, *J* = 7.6 Hz, 8H), 1.74 – 1.63 (m, 8H). ^{13}C NMR (75 MHz, DMSO-d_6) δ 154.60, 138.47, 122.96, 117.35, 99.46, 66.11, 52.82, 36.85, 26.74, 21.40. *ESI-MS* m/z: $[M−4Cl]^{4+}$ Calcd. for $C_{56}H_{80}N_4O_8$ 234.149; Found 234.170. $[M−3Cl]^{3+}$ Calcd. for $C_{56}H_{80}N_4O_8Cl$ 323.855; Found 323.877. $[M−2Cl]^{2+}$ Calcd. for $C_{56}H_{80}N_4O_8Cl_2$ 503.267; Found 503.306. HRMS (ESI) m/z: $[M−3Cl]^{3+}$ Calcd. for $C_{56}H_{80}ClN_4O_8$ 323.8549; Found 323.8545.

B. Spectral Characterization Data

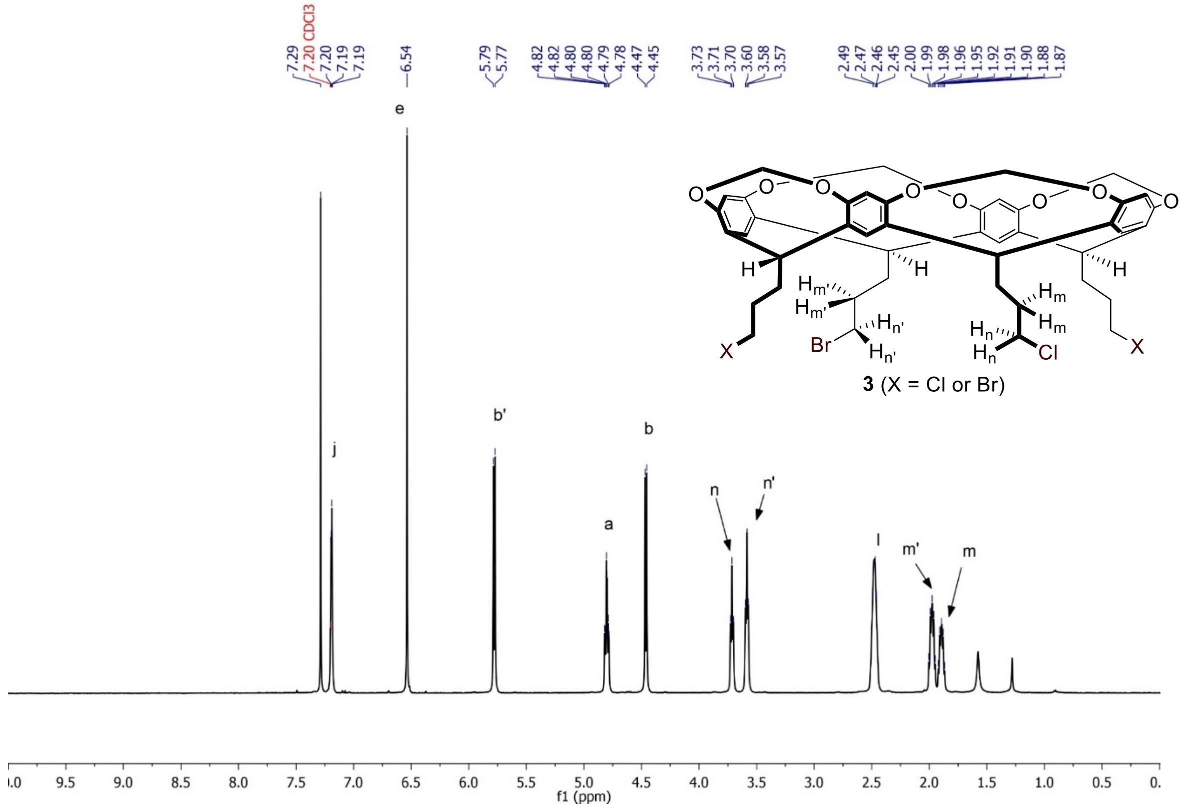

Figure S1. ^{1}H NMR spectrum (500 MHz, $CDCl_3$) of *tetra*-halide cavitand **3** (X ≈ Br, & 2Cl).q

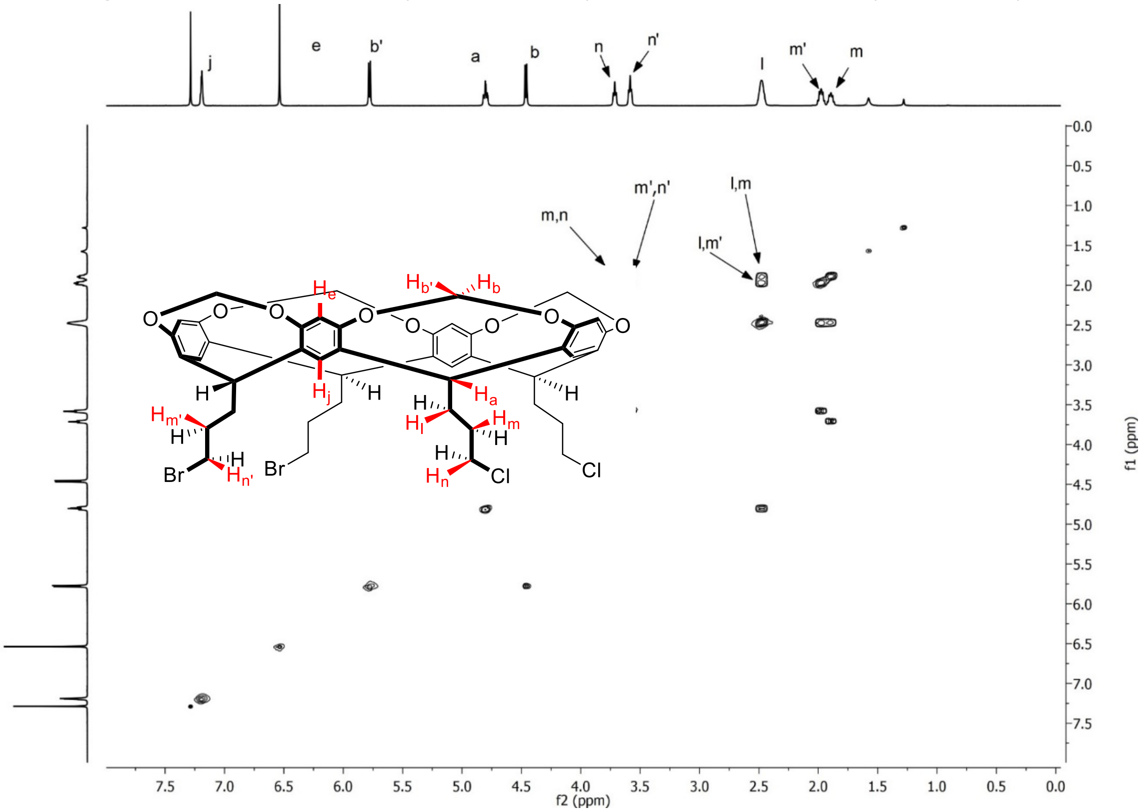

Figure S2. ^{1}H–^{1}H (COSY) NMR spectrum (500 MHz, $CDCl_3$) *tetra*-halide cavitand **3** (X ≈ 2Br & 2Cl).

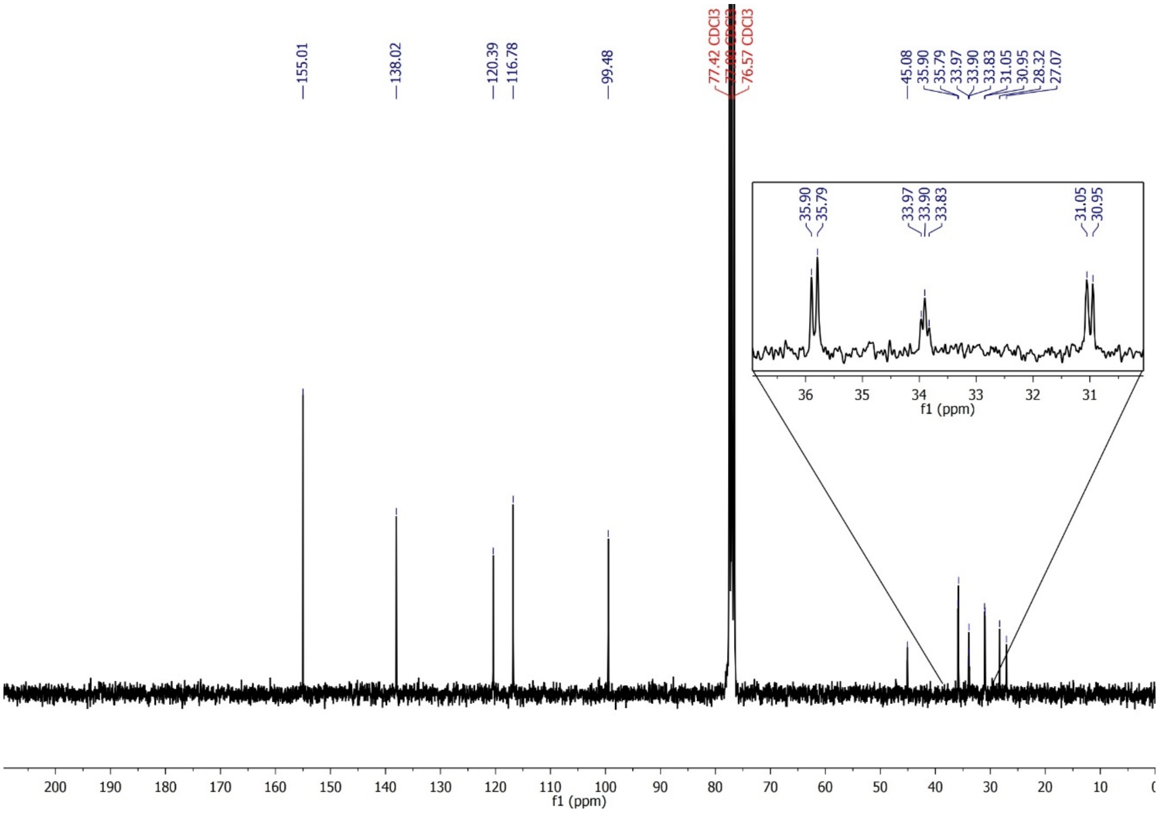

Figure S3. ^{13}C NMR spectrum ($CDCl_3$) of *tetra*-halide cavitand **3** (X ≈ 2Cl, 2Br).

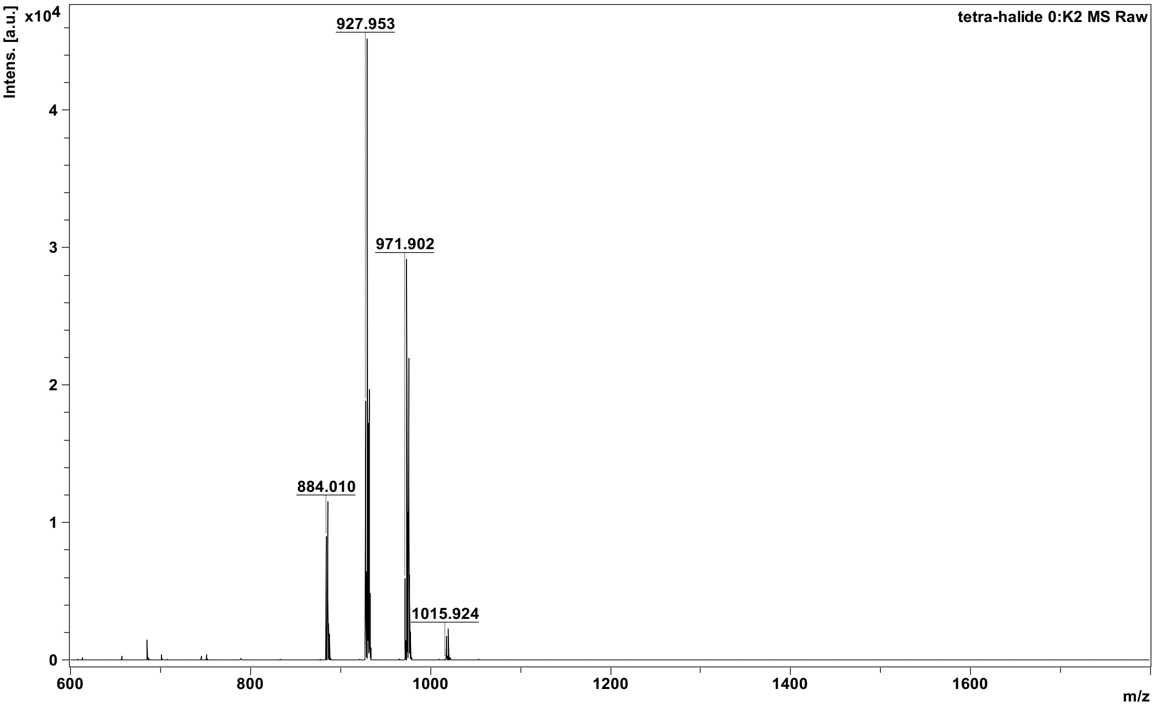

Figure S4. MALDI-TOF MS of *tetra*-halide cavitand **3**; (DCTB, 2:1:1) $Na^+CF_3CO_2^-$, $CHCl_3$.

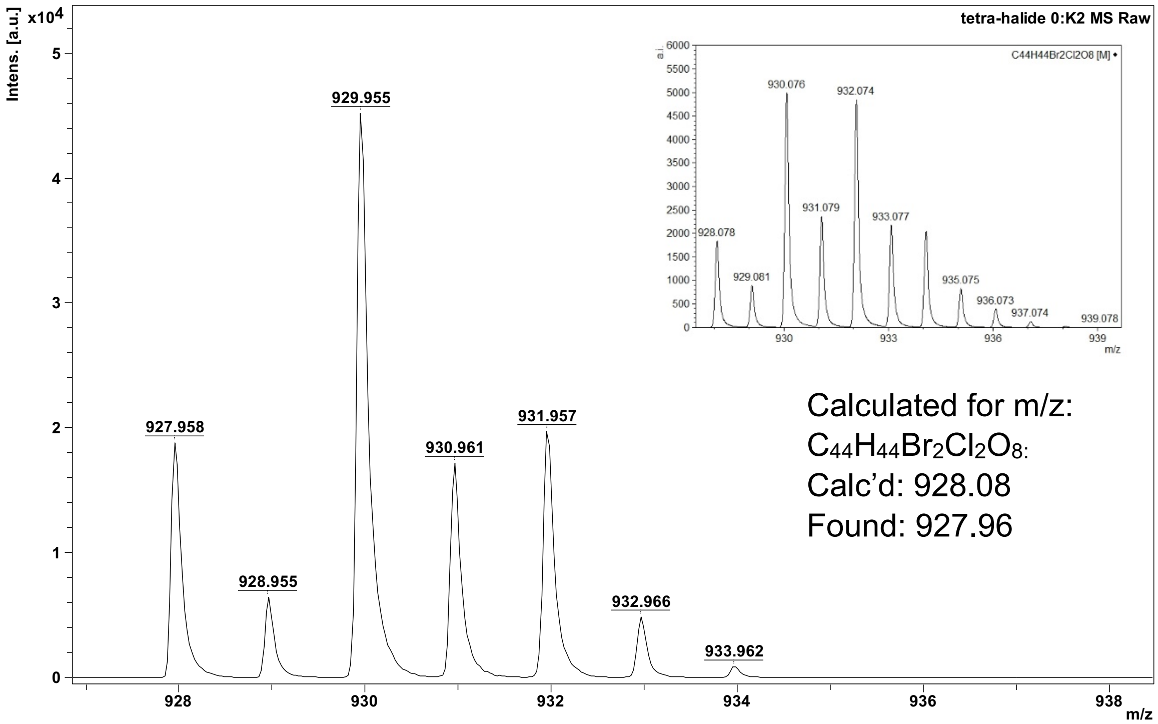

Figure S5. Expanded view of *tetra*-halide cavitand **3** (X = 2Br, 2Cl), [M–e⁻]⁺, with theoretical calculation inset.

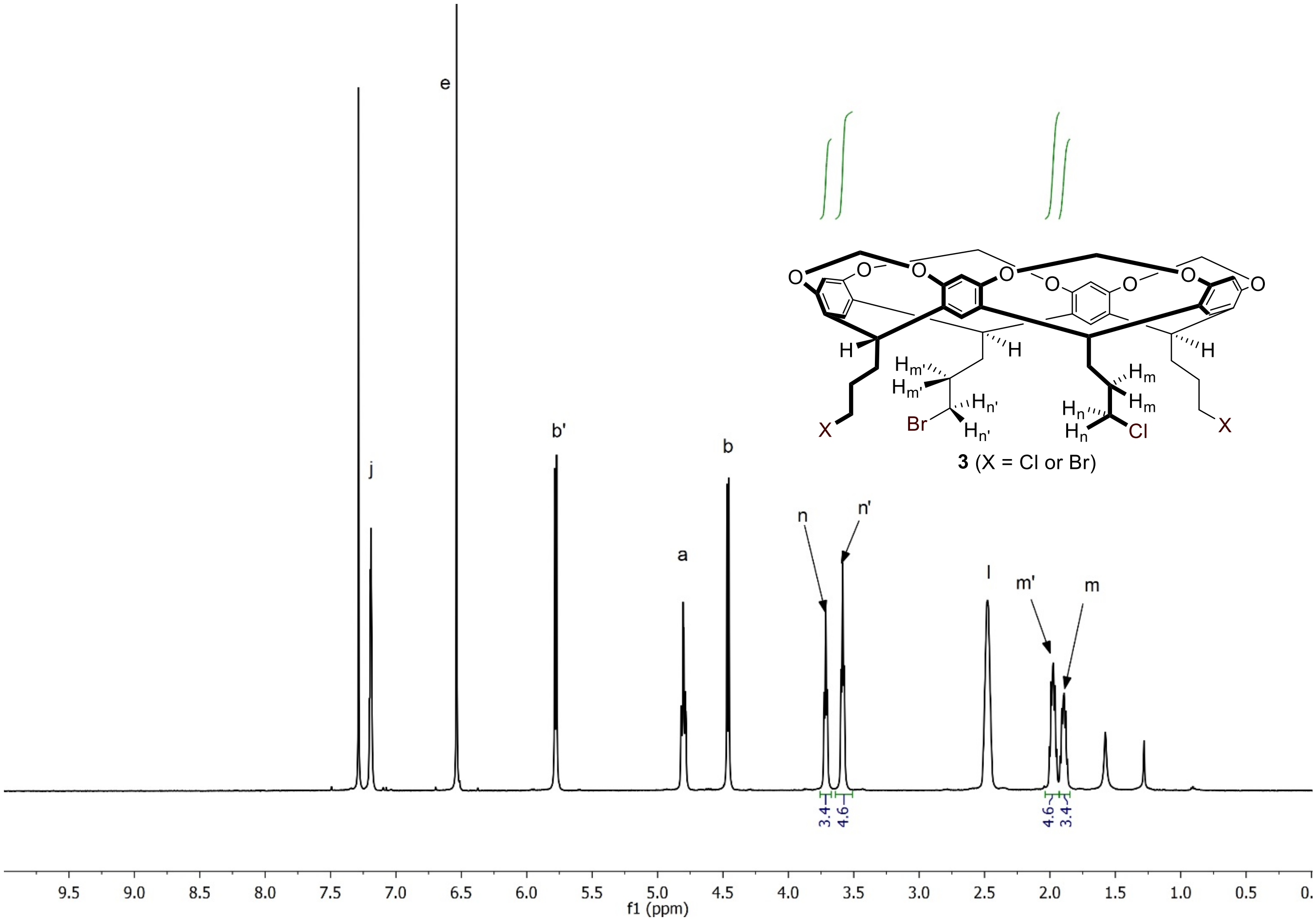

Figure S6. Integration of the methylene resonances indicating chloro or bromo substitution at the cavitand feet.

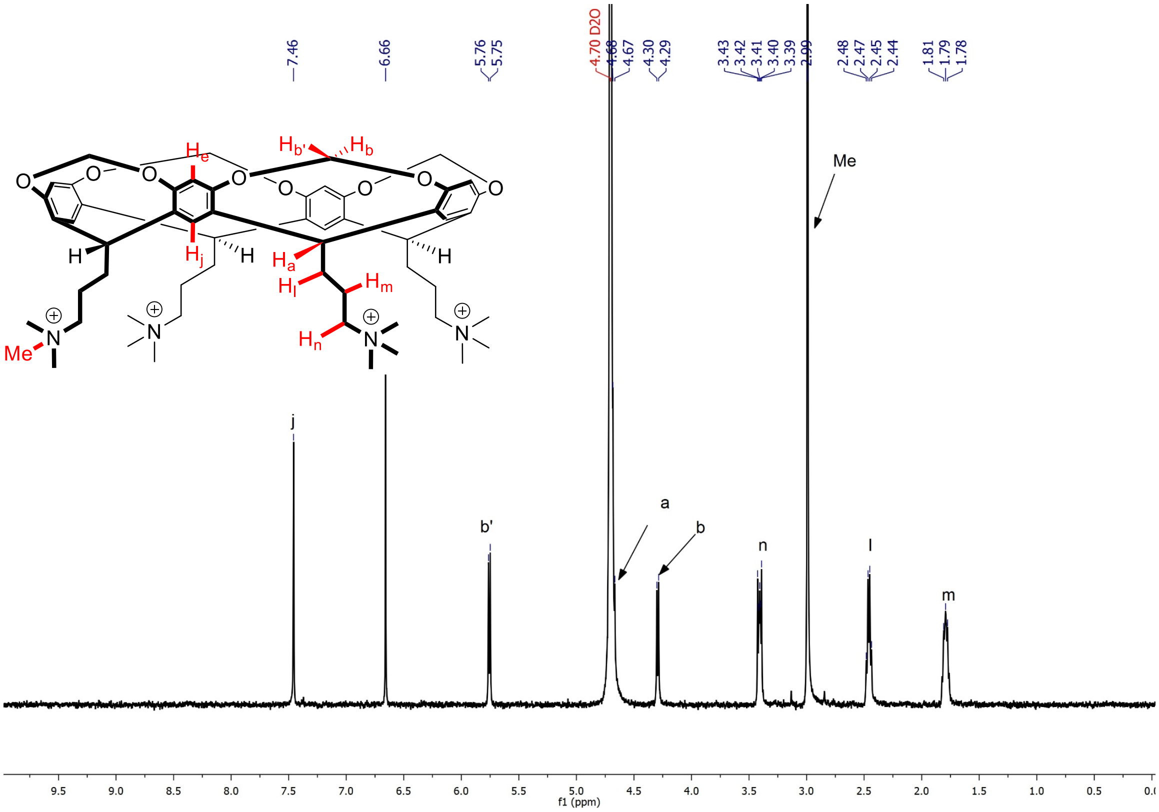

Figure S7. ^{1}H NMR spectrum (500 MHz, D_2O) of *tetrakis*-(trimethylammonium) chloride cavitand **1** (0.5 mM).

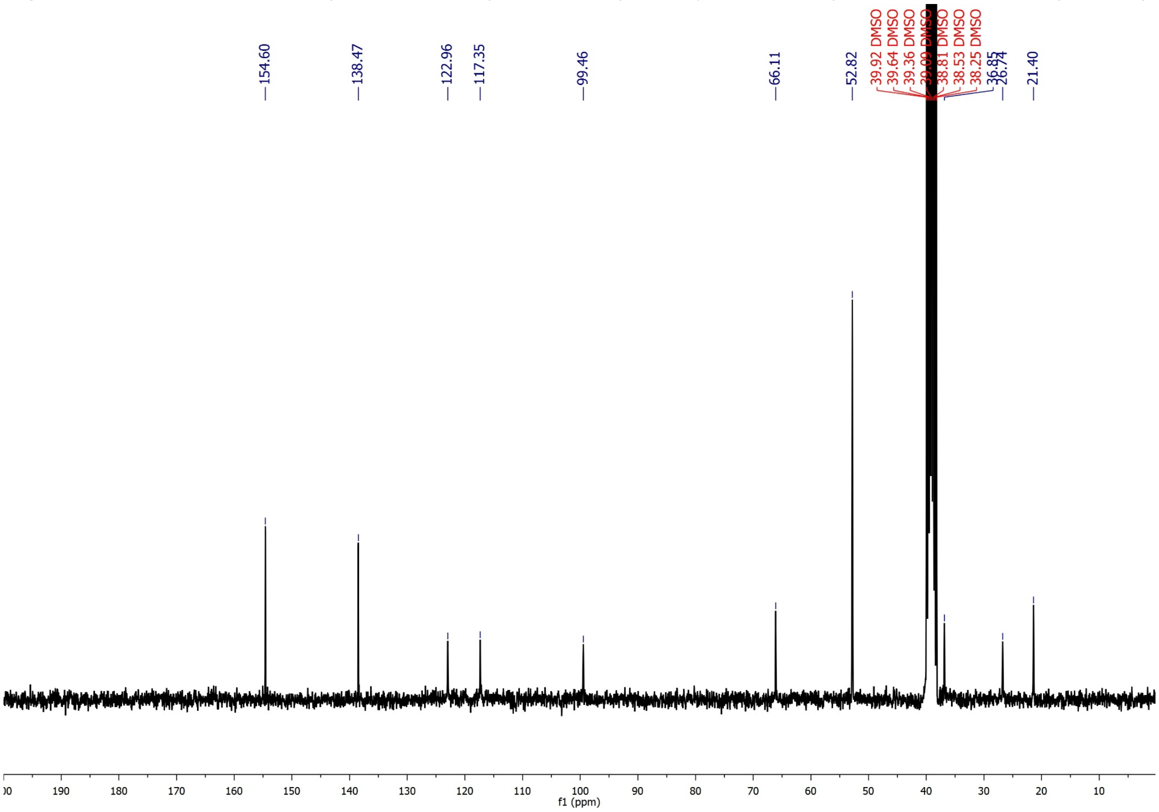

Figure S8. ^{13}C NMR spectrum (DMSO-d_6) of *tetrakis*-(trimethylammonium) chloride cavitand **1.**

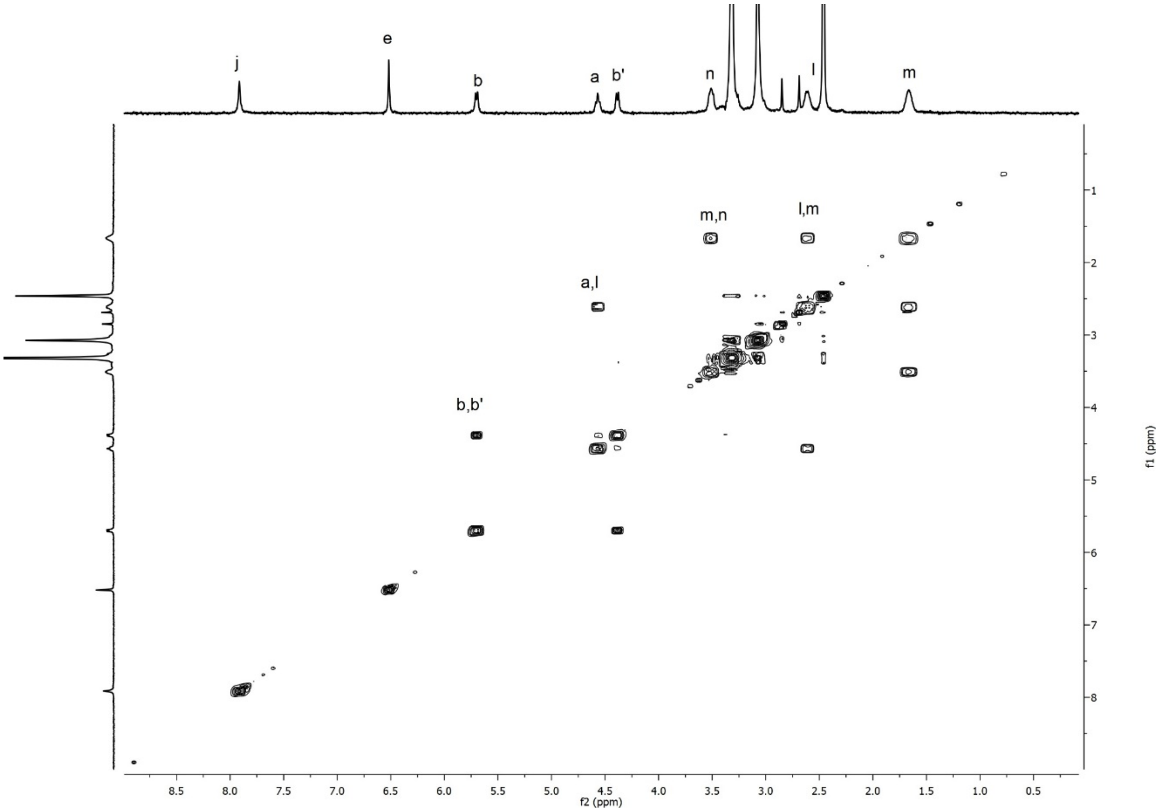

Figure S9. ^1H–^1H (COSY) NMR spectrum (DMSO-d_6) *tetrakis*-(trimethylammonium) chloride cavitand **4.**

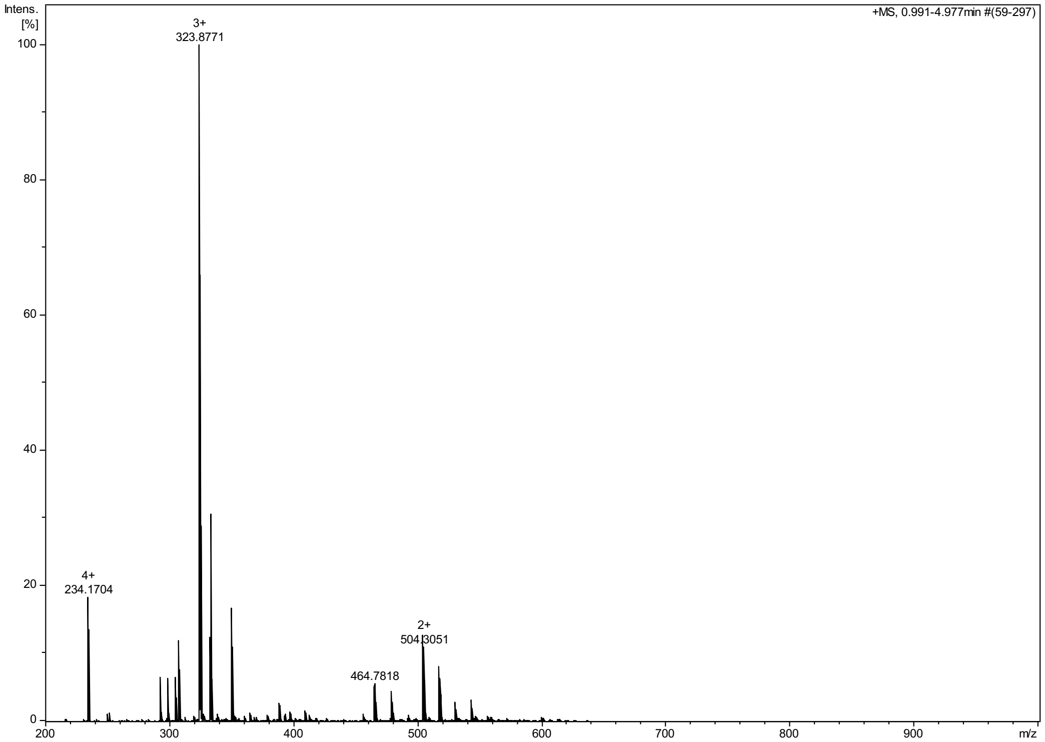

Figure S10. ESI- MS of *tetrakis*-(trimethylammonium) chloride cavitand **1** (20 μM, dH_2O).

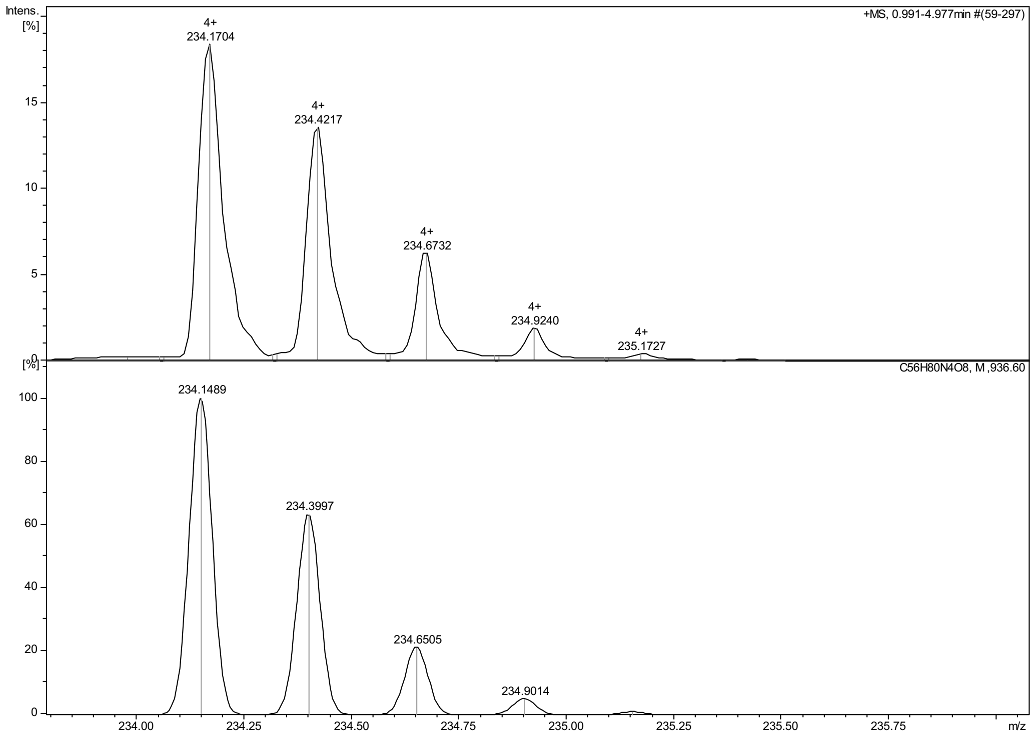

Figure S11. Expanded view of *tetrakis*-(trimethylammonium) chloride cavitand **1**, $[M-4Cl]^{4+}$, with theoretical calculation below.

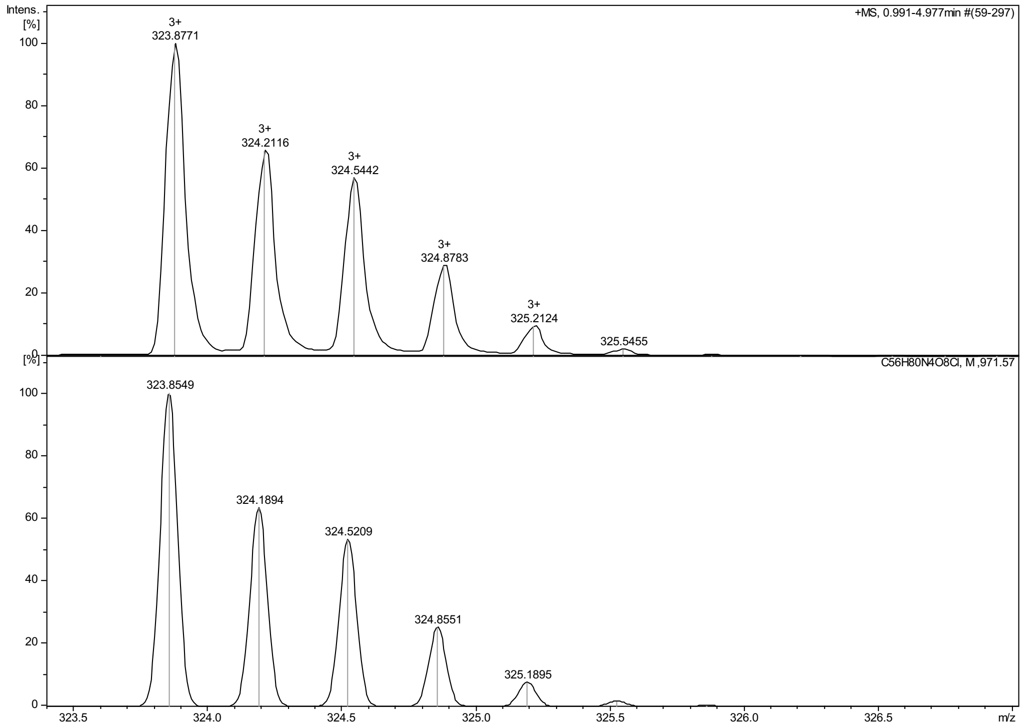

Figure S12. Expanded view of *tetrakis*-(trimethylammonium) chloride cavitand **1**, $[M-3Cl]^{3+}$, with theoretical calculation below.

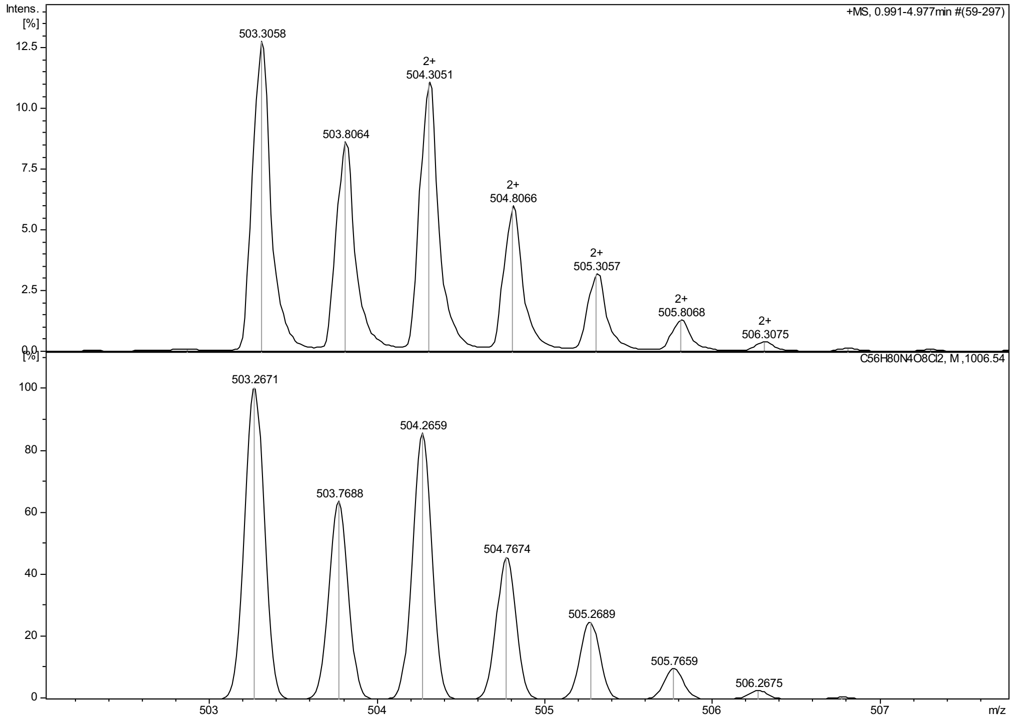

Figure S13. Expanded view of *tetrakis*-(trimethylammonium) chloride cavitand **1**, [M−42I]$^{2+}$, with theoretical calculation below.

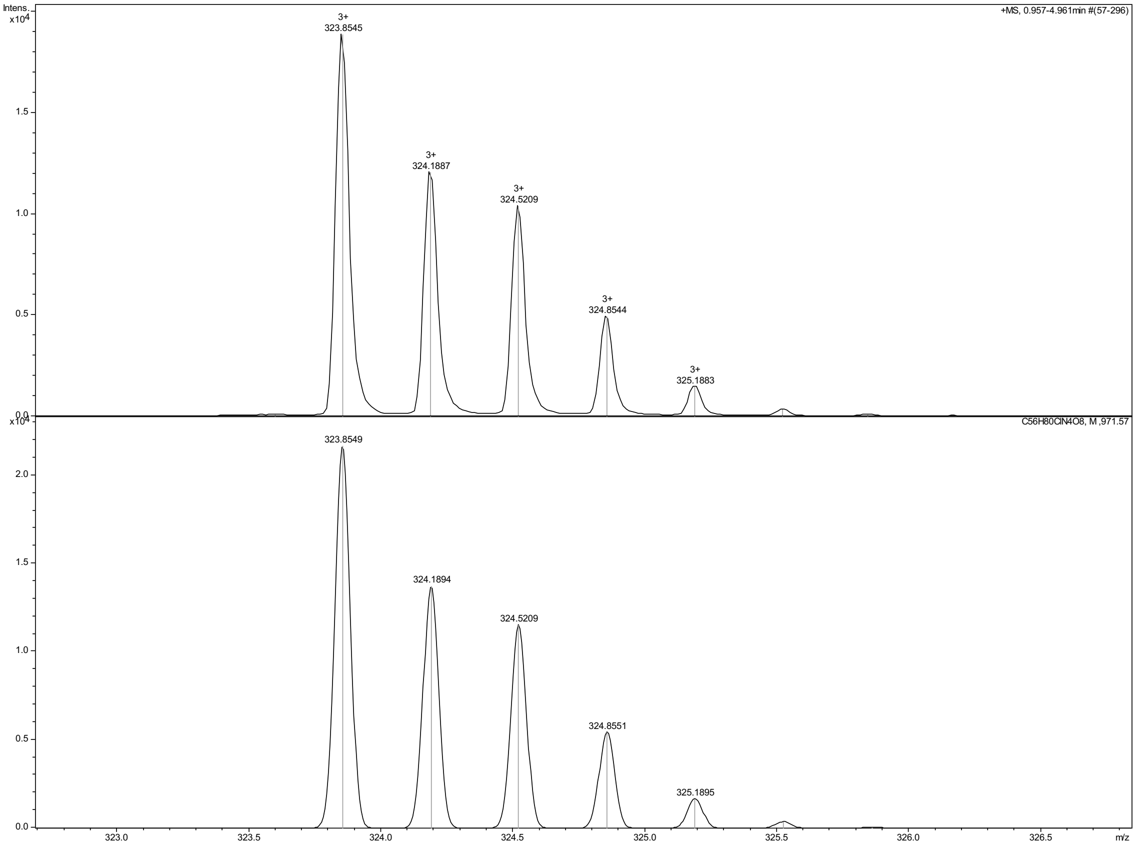

Figure S14. HRMS (ESI) of *tetrakis*-(trimethylammonium) chloride cavitand **1**, [M−3Cl]$^{3+}$, with theoretical calculation below.

3. Derivation of unscreened and screened (Debye-Hückel) Models

A. Derivation of screened reaction mode

Here we describe the derivation of the host/guest binding equilibrium expressions for both the unscreened and screened processes. As thermodynamics mandates, equilibrium is determined by minimization of the Gibbs free energy at constant temperature and pressure. To begin, we consider the partial molar Gibbs free energies of the species dissolved in water from which we can construct the total Gibbs free energy of the system. The partial molar Gibbs free energy of a solute (i) in dilute solution can be written as:

$$\bar{G}_i = \bar{G}_i^0 + RTln\left(\frac{[i]}{C_0}\right) \quad \text{(S1)}$$

where $\bar{G}_i^0$ is the free energy of the solute measured at the reference concentration C_0, and RT is the product of the gas constant and absolute temperature. Since this expression neglects added salts, this free energy corresponds to the solute free energy in an ideal unscreened solution. The addition of salts to solutions, however, is known to give rise to screened interactions between ions that leads to non-idealities that lower their free energies even in dilute solution. The dilute solution behavior of salts can be described by the Debye-Hückel limiting law, and modifies the partial molar Gibbs free energy for the screened electrolyte as:

$$\bar{G}_i = \bar{G}_i^0 + RTln\left(\frac{[i]}{C_0}\right) - \frac{\kappa q_i^2}{8\pi\varepsilon_0\varepsilon(1+\kappa\sigma_i)} \quad \text{(S2)}$$

where ε_0 is the permittivity of free space, ε is the dielectric constant of the solvent, q_i is the charge of i, σ_i is the Born radius (the ion-excluding radius) of i, and κ^{-1} is the Debye length describing the thickness of the counterion double layer that screens electrostatic interactions. The inverse Debye screening length κ is defined as:

$$\kappa = \left(\frac{\sum_i [i] q_i^2}{\varepsilon_0 \varepsilon RT}\right)^{1/2} \quad \text{(S3)}$$

We note that the Debye-Hückel equation only applies below electrolyte concentrations of ~100 mM. Additionally, this theory best describes monovalent ions of similar size. Nevertheless, we expect the theory to semi-quantitively/qualitatively account for the impact of charge screening in host/guest binding. We subsequently use Debye-Hückel theory here to assess the magnitude of the effect of screening on measured binding free energies.

Following from the properties of partial molar thermodynamic quantities, the total Gibbs free energy of a mixture can be expressed as the sum:

$$G = \sum_{i=1}^{n} N_i \bar{G}_i, \quad \text{(S4)}$$

where n is the number of components in the system, and N_i is the number of moles of component i. In the case of a cationic host (H^{4+}) / anionic guest (X^-) binding event to make the complex HX^{3+}, the free energy can be written as:

$$G = N_{H^{4+}}\bar{G}_{H^{4+}} + N_{X^-}\bar{G}_{X^-} + N_{HX^{3+}}\bar{G}_{HX^{3+}} + N_{Na^+}\bar{G}_{Na^+} + N_w\bar{G}_w$$
$$= \left(N_{H^{4+}}^* - \lambda\right)\bar{G}_{H^{4+}} + (N_{X^-}^* - \lambda)\bar{G}_{X^-} + (N_{HX^{3+}}^* + \lambda)\bar{G}_{HX^{3+}} + N_{Na^+}\bar{G}_{Na^+} + N_w\bar{G}_w, \quad \text{(S5)}$$

where the subscripts w and Na^+ denote the solvent water and non-reacting sodium counterion, the superscript $*$ indicates the initial mole numbers of a specified component, and λ indicates the extent of reaction in moles. The minus sign in front of λ for the components H^{4+} and X^- is a result of them being 'consumed' during the binding reaction, while the positive sign in front of λ for HX^{3+} is a result of the complex being a product of the reaction. Minimizing the total Gibbs free energy with respect to λ yields the condition for reaction equilibrium as:

$$\frac{\partial G}{\partial \lambda} = 0 = \bar{G}_{HX^{3+}} - \bar{G}_{H^{4+}} - \bar{G}_{X^-} \qquad \text{(S6)}$$

While the partial molar Gibbs free energies are themselves dependent on λ, the sum of their derivatives (i.e., $\sum_{i=1}^{n} N_i \partial \bar{G}_i / \partial \lambda$) is zero as a result of the Gibbs-Duhem equation and therefore do not appear in the equilibrium condition above. In the case of the unscreened equilibrium, substituting the expressions for the partial molar Gibbs free energies of each component (Eq. (S1)) into the reaction equilibrium condition (Eq. (S6)) yields:

$$0 = \bar{G}^0_{HX^{3+}} + RTln\left(\frac{[HX^{3+}]}{C_0}\right) - \bar{G}^0_{H^{4+}} - RTln\left(\frac{[H^{4+}]}{C_0}\right) - \bar{G}^0_{X^-} - RTln\left(\frac{[X^-]}{C_0}\right) \qquad \text{(S7)}$$

Rearranging this expression, we obtain the unscreened reaction equilibrium product:

$$\frac{[HX^{3+}]}{[H^{4+}][X^-]} = C_0^{-1} exp\left(-\frac{\bar{G}^0_{HX^{3+}} - \bar{G}^0_{H^{4+}} - \bar{G}^0_{X^-}}{RT}\right) = K^{U,0}_{X^-} \qquad \text{(S8)}$$

corresponding to Eq. 5 in the main text. Here $K^{U,0}_{X^-}$ corresponds to the unscreened reaction equilibrium constant measure relative to the reference concentration C_0. If instead the full screened model expressions (eq. (S2)) for the partial molar Gibbs free energies are substituted into the reaction equilibrium condition (eq. (S6)), we get:

$$0 = \bar{G}^0_{HX^{3+}} + RTln\left(\frac{[HX^{3+}]}{C_0}\right) - \frac{\kappa q^2_{HX^{3+}}}{8\pi\varepsilon_0\varepsilon(1+\kappa\sigma_{HX^{3+}})} - \bar{G}^0_{H^{4+}} - RTln\left(\frac{[H^{4+}]}{C_0}\right) + \frac{\kappa q^2_{H^{4+}}}{8\pi\varepsilon_0\varepsilon(1+\kappa\sigma_{H^{4+}})}$$
$$-\bar{G}^0_{X^-} - RTln\left(\frac{[X^-]}{C_0}\right) + \frac{\kappa q^2_{X^-}}{8\pi\varepsilon_0\varepsilon(1+\kappa\sigma_{X^-})} \qquad \text{(S9)}$$

Rearranging this expression, we obtain the screened reaction equilibrium product as:

$$\frac{[HX^{3+}]}{[H^{4+}][X^-]} = C_0^{-1} exp\left(-\frac{\bar{G}^0_{HX^{3+}} - \bar{G}^0_{H^{4+}} - \bar{G}^0_{X^-}}{RT}\right) exp\left[\frac{\kappa}{8\pi\varepsilon_0\varepsilon RT}\left(\frac{q^2_{HX^{3+}}}{1+\kappa\sigma_{HX^{3+}}} - \frac{q^2_{H^{4+}}}{1+\kappa\sigma_{H^{4+}}} - \frac{q^2_{X^-}}{1+\kappa\sigma_{X^-}}\right)\right],$$
$$K^{S,0}_{X^-} exp\left[\frac{\kappa}{8\pi\varepsilon_0\varepsilon RT}\left(\frac{q^2_{HX^{3+}}}{1+\kappa\sigma_{HX^{3+}}} - \frac{q^2_{H^{4+}}}{1+\kappa\sigma_{H^{4+}}} - \frac{q^2_{X^-}}{1+\kappa\sigma_{X^-}}\right)\right] = K^S_{X^-} \qquad \text{(S10)}$$

corresponding to Eq. (6) in the main text. Similar to the unscreened reaction, $K^{S,0}_{X^-}$ corresponds to the unscreened reaction equilibrium constant measure relative to the reference concentration C_0, and as such is determined by the same expression as for $K^{U,0}_{X^-}$ above. Screening by added electrolytes in Eq. (S10), however, gives rise to $K^S_{X^-}$ falling with increasing salt concentration, that is host/guest binding is weakened by screening. Given that the unscreened model does an excellent job at describing the

experimental results, we expect the fitted values of $K_{X^-}^{S,0}$ will tend to be greater than $K_{X^-}^{U,0}$ so that once the screened model is fitted to the data the resultant concentration dependent values of $K_{X^-}^{S}$ will be comparable to the $K_{X^-}^{U,0}$. If $K_{X^-}^{U,0}$ and $K_{X^-}^{S,0}$ can be regarded as corresponding the free energies of host/guest binding in the absence of added solutes that can screen interactions, the question follows, by how much do $K_{X^-}^{U,0}$ and $K_{X^-}^{S,0}$ differ?

B. Fitting of screened reaction model to experimental data

In difference to the unscreened model, the screened reaction equilibrium model is highly non-linear as a result of the concentration dependence of the equilibrium constants as described by Eq. (S10). Specifically, since the values of κ depend on the equilibrium concentrations of all the species in solution (including sodium), an estimate of the distribution of bound and unbound hosts and guests must be made in order to evaluate $K_{X^-}^{S}$ at every added guest concentration. Here we use an iterative approach to determine the concentrations of the charged species in solution. Our initial guess for these concentrations is made using the unscreened model. We then substitute the host, guest, and complex concentrations predicted by the unscreened model into Eq. (S10) to evaluate the concentration dependent $K_{X^-}^{S}$. These yield a new set of estimates for the distribution of reacting species in solution, which in turn can be substituted back into the screened model to evaluate new $K_{X^-}^{S}$'s. The procedure is iterated until the distribution of host, guest, and complex species converge to a stable set of concentrations, which subsequently are the equilibrium concentrations. Approximately 5 iterations are required to converge the solution within the accuracy of the Microsoft Excel spreadsheet used to solve these equations.

4. Analytical Data

A. NMR Titration data

All solutions were prepared as described in the solution preparation procedures (Section 1.C). To determine guest affinity for host **1**, ^{1}H NMR spectroscopy titrations were first conducted in unbuffered D_2O and were performed on ~0.4 mM host from a concentrated stock of host typically prepared at ~2–4 mM. Dilution of the host solution during all titrations was kept below 10%.

a. Determining the affinity of chloride to cavitand **1**

Solutions of host **1** showed concentration dependent shifts in the peaks H_j and H_l (Figure S15); however, this shift was not due to host aggregation. Rather, the 2D Diffusion Oriented Spectroscopy (DOSY) spectra of both a 0.5 mM and a 25 mM solution of **1** in D_2O (Figure S16 and Figure S17) revealed very similar diffusion coefficients: $D = 2.79 \times 10^{-6}$ (R_h = 0.78 nm) and 2.64×10^{-6} cm^2 s (R_h = 0.83 nm) respectively. This modest difference is within the expected error of ±10%. Thus, the presumed spherical host is monomeric at both concentrations.

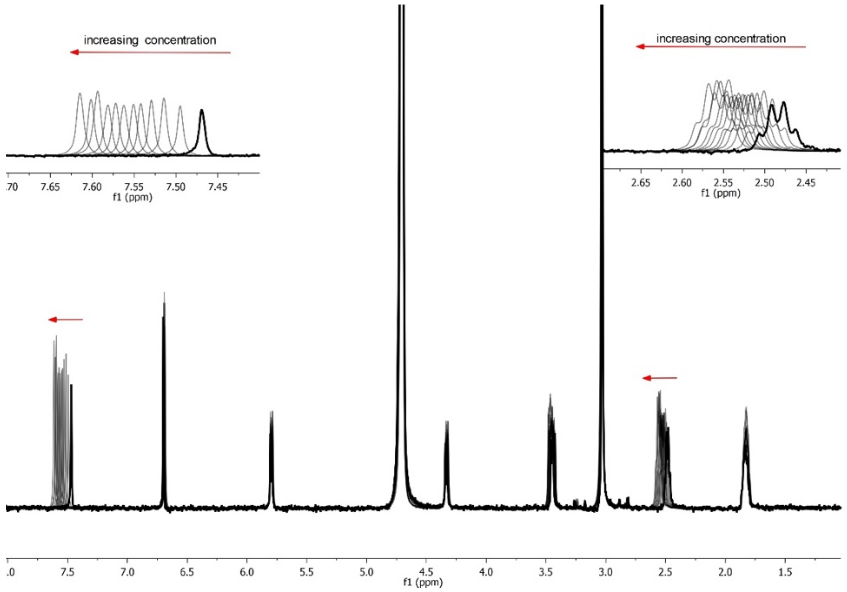

Figure S15: Superimposed image of ^{1}H NMR of **1** at 0.25 mM and higher concentrations. Expansions show the upfield shift of H_j and H_l upon addition of host **1** and concomitant addition of Cl^- ions.

It is apposite to note that all ^{1}H NMR titrations in unbuffered D_2O were relative to the *tetra*-chloride salt of the host. Figure S15 shows the result of an experiment whereby the concentration of host was incrementally increased from 0.25 mM concentration to 16 mM by the addition of aliquots of a concentrated stock solution (50 mM). Since the host remains monomeric during this "titration", the concentration dependent signal shifts observed are the result of increased Cl^- complexation at higher concentrations of both species. As confirmation, monitoring the Δδ values for H_l and H_j of host **1** (see Scheme S1) as its concentration was increased gave essentially the same isotherm as titration of host **1** with NaCl (*vide infra*), when accounting for the concentration of the anion (e.g. $[Cl^-]_{added} = 4 \times [H]$).

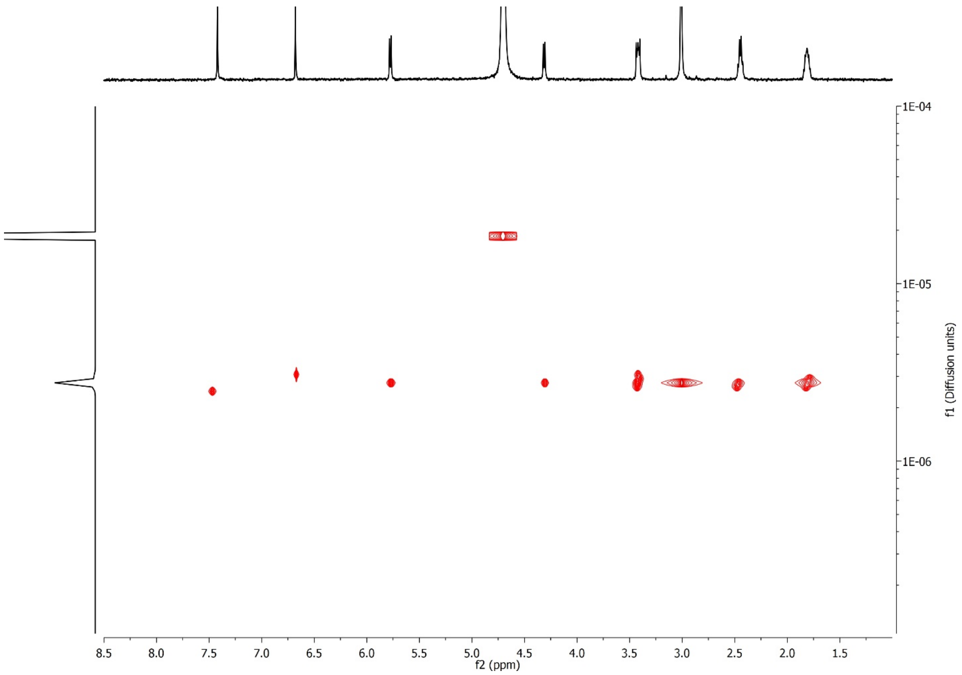

Figure S16: 2D DOSY NMR of **1** (0.5 mM) in unbuffered D_2O

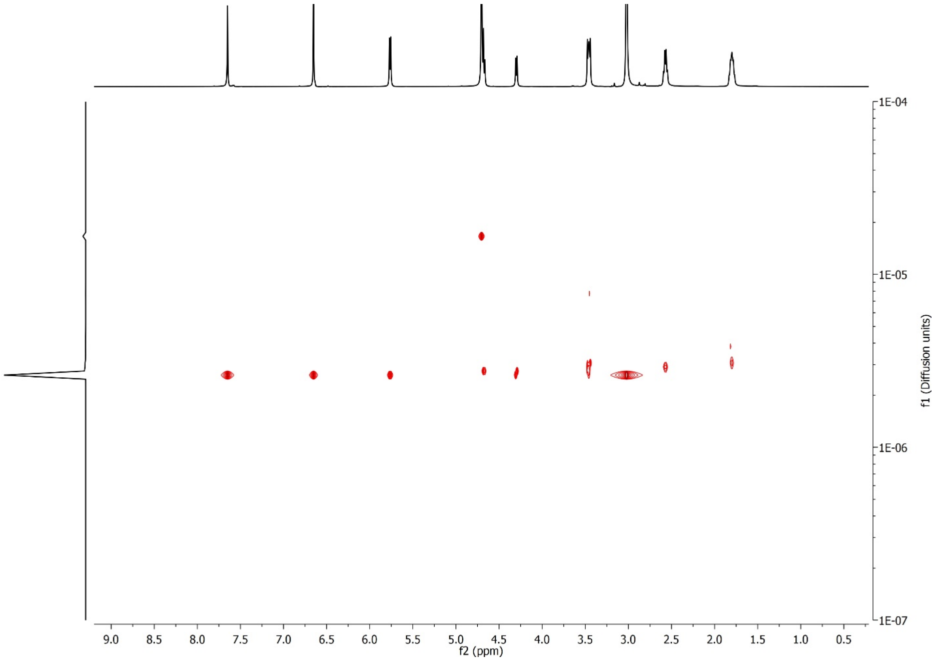

Figure S17. 2D DOSY NMR of **1** (25 mM) in unbuffered D_2O.

The NMR spectroscopy signal shifts obtained from the titration with NaCl were fitted by nonlinear regression analysis to the 1:1 binding stoichiometry model by Eq. S11 using either the solver in Excel[5,6] or BINDFIT[4]

$$\Delta\delta_{obs} = \frac{\Delta\delta_{max}}{\frac{2}{K_aG_t - K_aH_t - 1 + \sqrt{(1 - K_aG_t - K_aH_t)^2 + 4K_aH_t}} + 1} \qquad \text{(S11)}$$

in which H_t and G_t are the total amount of host and guest; fitting the experimentally derived binding isotherm for the change in signal shift $\Delta\delta_{obs}$ versus G_t yielded the remaining two unknowns, the binding constant (generic K_a) and the maximal shift in the NMR signal ($\Delta\delta_{max}$). The following assumptions were made:

$$G_t = [G] + [HG]_{crown} + n[HG_n]_{other} \qquad \text{(S12)}$$

$$G_t \approx [G] + [HG]_{crown} \text{ since } [G] + [HG]_{crown} >> n[HG_n]_{other} \qquad \text{(S13)}$$

where $[G_t]$ is the total guest concentration, $[HG]_{crown}$ is the concentration of the complex with the anion binding to the crown of four cationic groups of the cavitand, and $[HG_n]_{other}$ is the (low) concentration of complexes arising from non-specific binding to the host.

However, to accurately determine the affinity of the counterion of the host, one must consider its intrinsic counter anion. Take the case of **1** (counterion, Cl^-), for example. Since there are four equivalents of (intrinsic) Cl^- present at the start of the titration, the real initial point corresponding to the theoretical ^{1}H NMR signal from the chloride-free host (δ, ppm) is not known. Under normal circumstances (e.g., a neutral host), the initially observed NMR signal to be monitored (δ_{obs}) corresponds to the situation where the guest total G_t = 0. Since in the case here $G_t \neq 0$, during titration the resulting maximum shift in the NMR signal (δ_{max}) is lowered and the binding isotherm flattened, i.e., the observed binding constant reduced. Eq. S14 shows the effect the counterion has on δ_{obs}, where x_H is the mole fraction of the free

host, δ_H is the signal (ppm) of the free host, χ_{HG} is the mole fraction of the host-guest complex, and δ_{HG} is the corresponding signal of the host-guest complex. If $\chi_{HG} = 0$, then $\delta_{obs} = \delta_H$. Changes in δ_{obs} ($\Delta\delta_{obs}$) are thus the result of changes associated with changes in δ_{HG} and are defined by (Eq. S15, the derivations of which have been discussed in more detail by Thordarsson:[5]

$$\delta_{obs} = \chi_H\delta_H + \chi_{HG}\delta_{HG} = (1-\chi_{HG})\delta_H + \chi_{HG}\delta_{HG} \quad \text{(S14)}$$

$$\Delta\delta_{obs} = \Delta\delta_{HG}\chi_{HG} \quad \text{(S15)}$$

Therefore, to determine the binding affinity of Cl^- to the host, the initial observed point of the titration was set to correspond to four equivalents of chloride, and the true (theoretical) δ value for the initial point corresponding to zero equivalents of chloride allowed to float when solving for Δδ in Eq. S11. This led to a binding isotherm that accounts for the presence of stoichiometric Cl^- at the start of the titration, and an obtained value representing the actual affinity. The resulting isotherm from an independent (single peak) fitting of the titration data for H_j and H_l (for proton designations see Scheme S1) using Eq. S11 is shown in Figure S18, where the $\Delta\delta_{max}$ for H_j is significantly larger than for H_l (0.276 vs 0.149 ppm). For both signals, the saturation of the curve and the obtained affinity values (316 M^{-1} vs 310 M^{-1}) were identical and within error. Global (multiple peak) fitting (Figure S19) was also applied so that large errors, resulting from situations where one or both peaks did not shift significantly ($\Delta\delta_{max} < 0.05$ ppm), could be mitigated. In the case at hand, as anticipated, global fitting of H_j and H_l was successful and had essentially no effect on the obtained affinity value of Cl^- (315 M^{-1}). Based on this result, global fitting was applied in all instances, with typically improved errors and reproducibility.[5,7] Data for the titration of sodium chloride to host **1** was collected from multiple experiments to give an average anion affinity of 290 ± 20 M^{-1}. A representative example titration is shown in Figure S20 and the corresponding BINDFIT isotherm and residuals plot in Figure S21.

An important observation during multiple titrations was that the change in pD of the unbuffered solutions used was less than ~0.4 pD units and fell between the values ~5.6 and ~6.8. As the pD of the solutions was largely unaffected by titration of salts to the host, and as host **1** contains no ionizable groups, the use of a buffer was not strictly necessary.

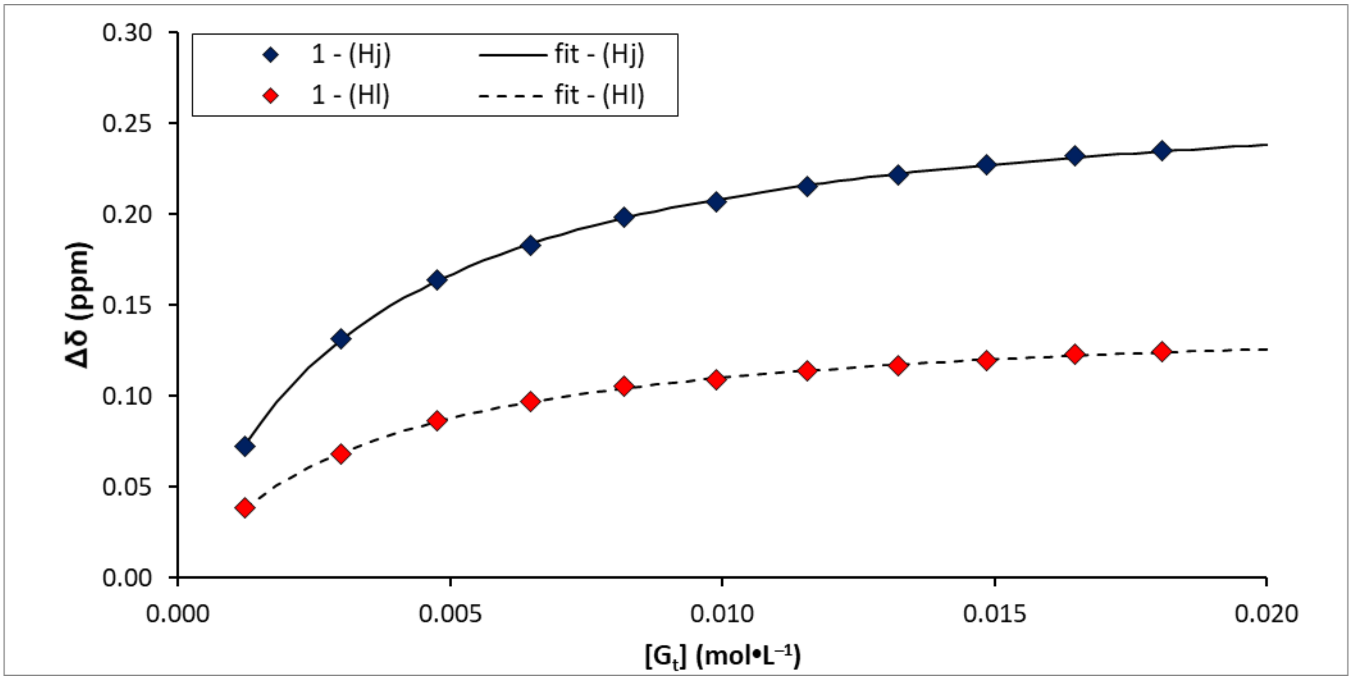

Figure S18. Independently fitting the shift of H_j and H_l during the titration of **1** with 300 mM NaCl (up to 59 added equivalents). K_{Cl^-} = 316 M^{-1} and 310 M^{-1} (H_j, and H_l, respectively).

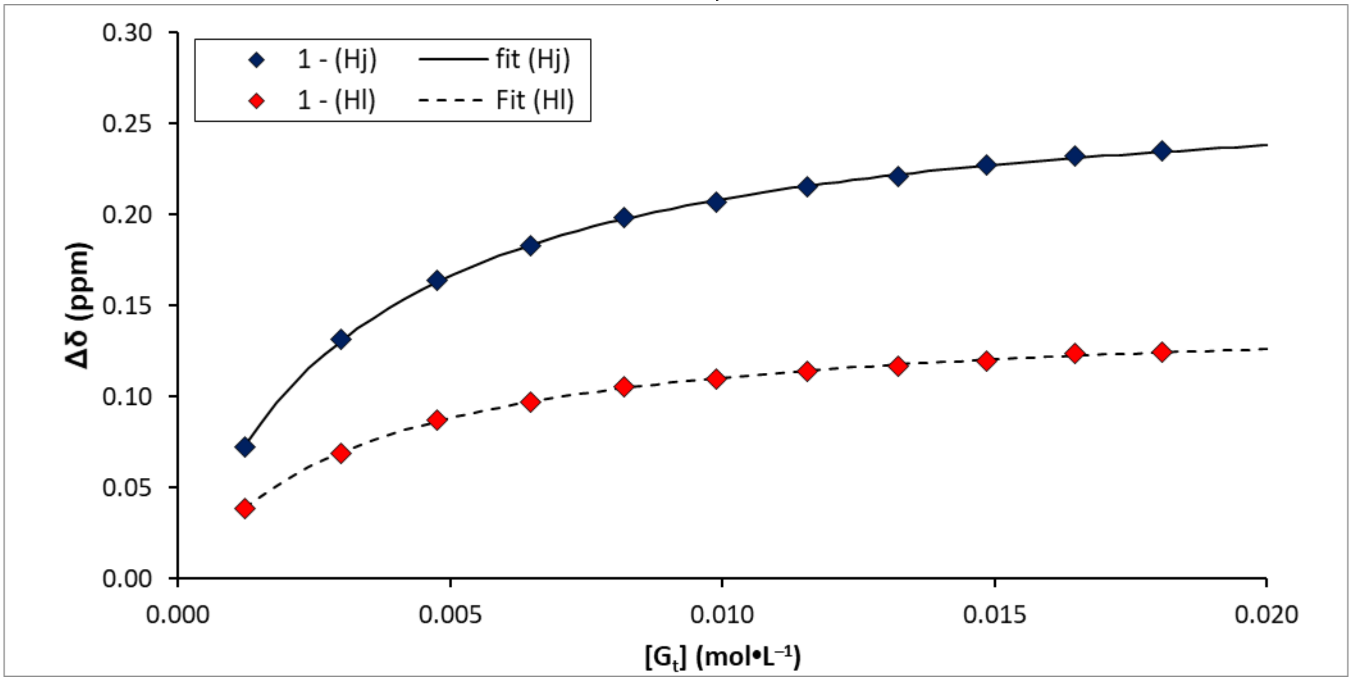

Figure S19: Global fitting of H_j and H_l during the titration of **1** with 300 mM NaCl (up to 59 equivalents). $K_{Cl^-}^{U,0}$ = 315 M^{-1}.

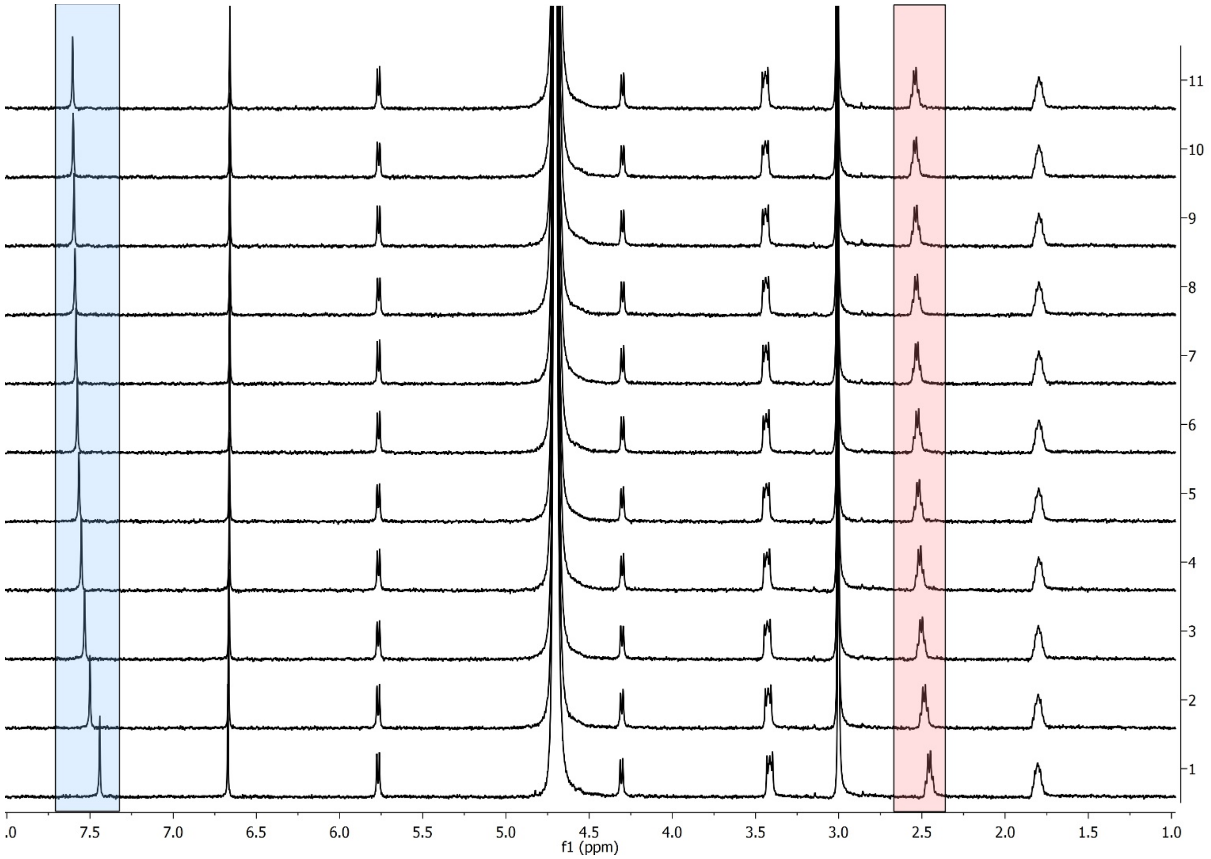

Figure S20. Representative titration of **1** with 300 mM NaCl (up to ~59 equiv.). H_j designated with blue box, H_l in red.

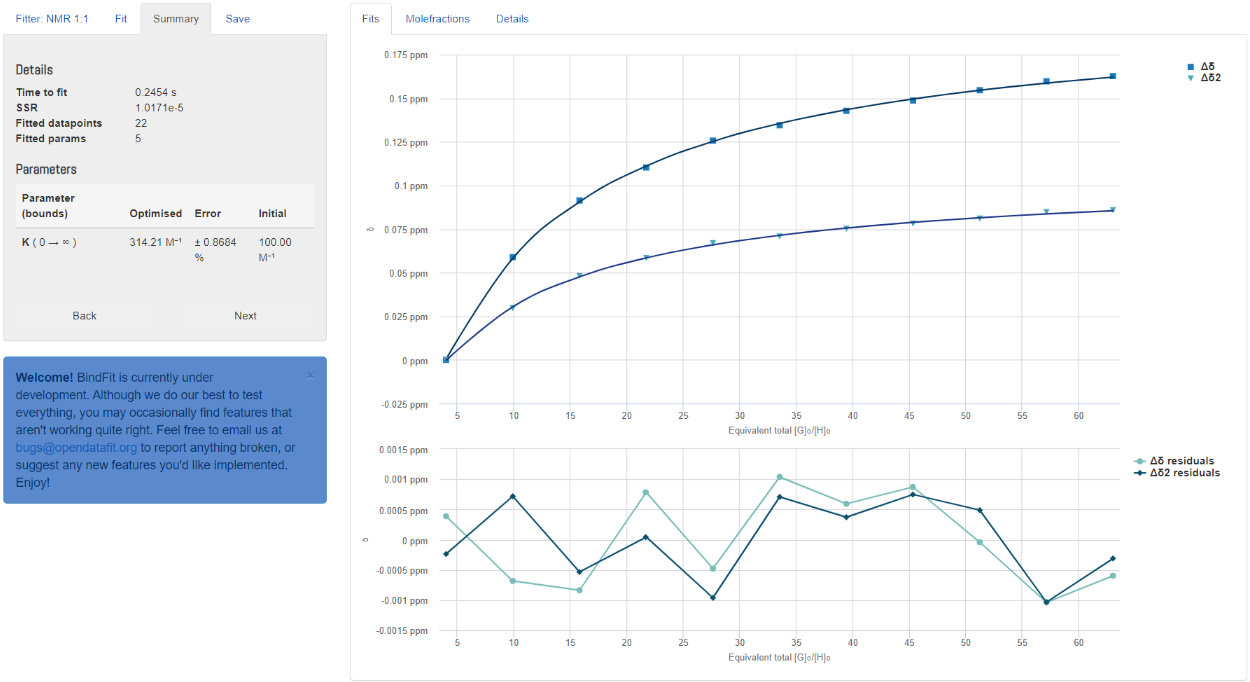

Figure S21. Fit of the data for H_j and H_l from Figure S20 to a 1:1 binding model, floating δ_H and starting with the first point at 4 equiv. Cl^- (up to 59 equiv. NaCl added). http://app.supramolecular.org/bindfit/view/b833f7df-d225-4ff6-a03d-32d738a8c952.[4,5,8] (Bindfit and supramolecular.org are part of OpenDataFit.org and are open source & open access).

b. Determining the effect of the cations on chloride binding

To determine what effect the counter-cation had on anion complexation to host **1**, titrations were also conducted with the chloride salts of lithium (Li^+), potassium (K^+), cesium (Cs^+) and tetramethyl ammonium ($^+N(CH_3)_4$) (Figure S22–Figure S29). Titrations were conducted in (at least) triplicate. and in each instance data was fitted globally. The results ($K_{Cl^-}^{U,0}$, CV%, Δ*G*) are summarized in Table S1:

Table S1: Summary of ^{1}H NMR titration data for binding constant determination of Cl^- to host **1** (0.4 mM, D_2O) $K_{Cl^-}^{U,0}$value obtained by accounting for four equiv. of Cl^- and floating initial titration point.

Cation	$K_{Cl^-}^{U,0}$ (M^{-1})	CV%[a]	$\Delta G_{Cl^-}^{U,0}$ (kJ·mol^{-1})
Li^+	228 ± 8	4	-13.46 ± 0.09
Na^+	290 ± 20	7	-14.06 ± 0.17
K^+	271 ± 11	4	-13.89 ± 0.10
Cs^+	263 ± 28	11	-13.81 ± 0.26
$^+N(CH_3)_4$	246 ± 28	11	-13.65 ± 0.28

a) CV% obtained from at least three measurements.

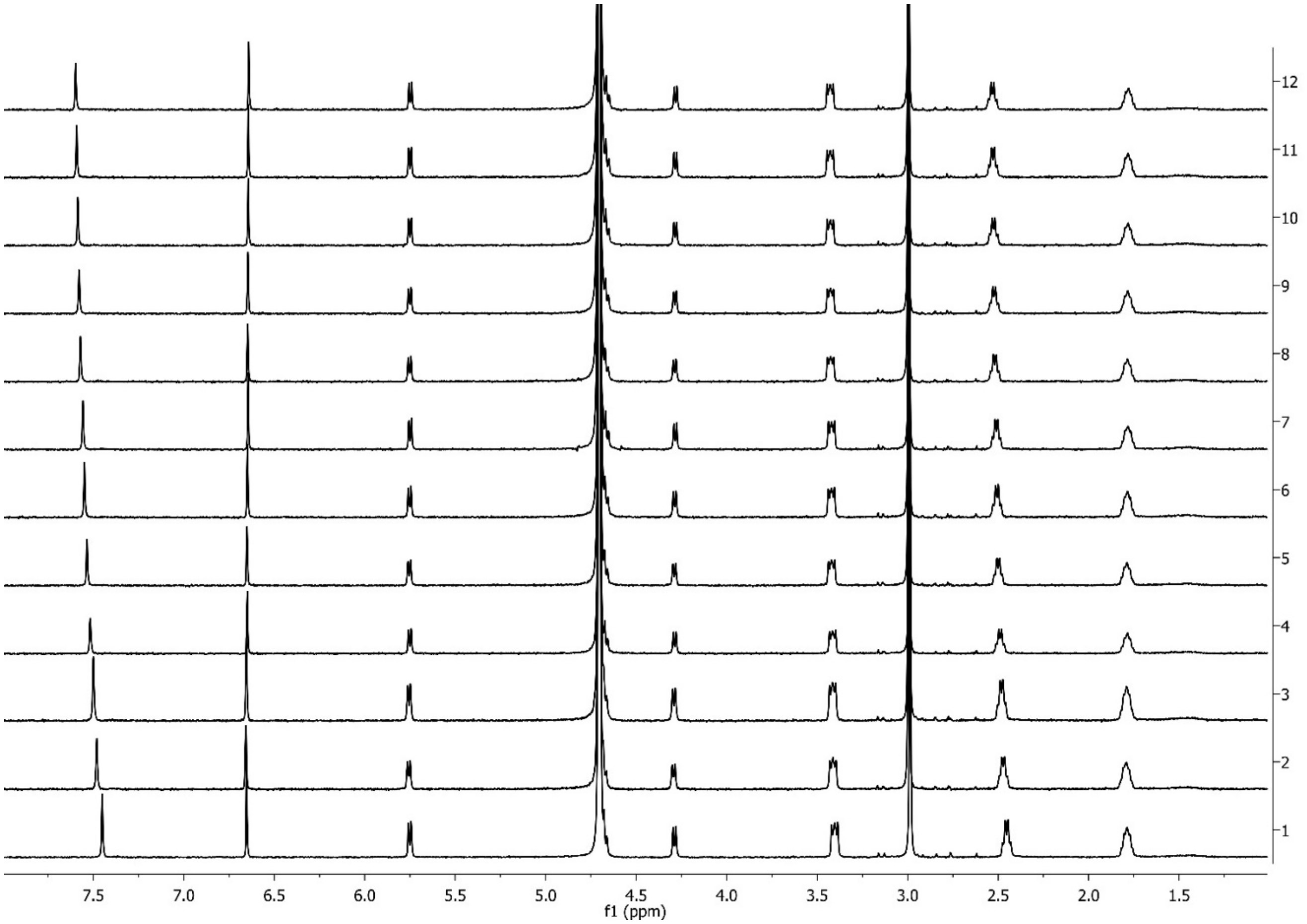

Figure S22. Representative titration of **1** with 250 mM LiCl (up to ~63 added equiv.).

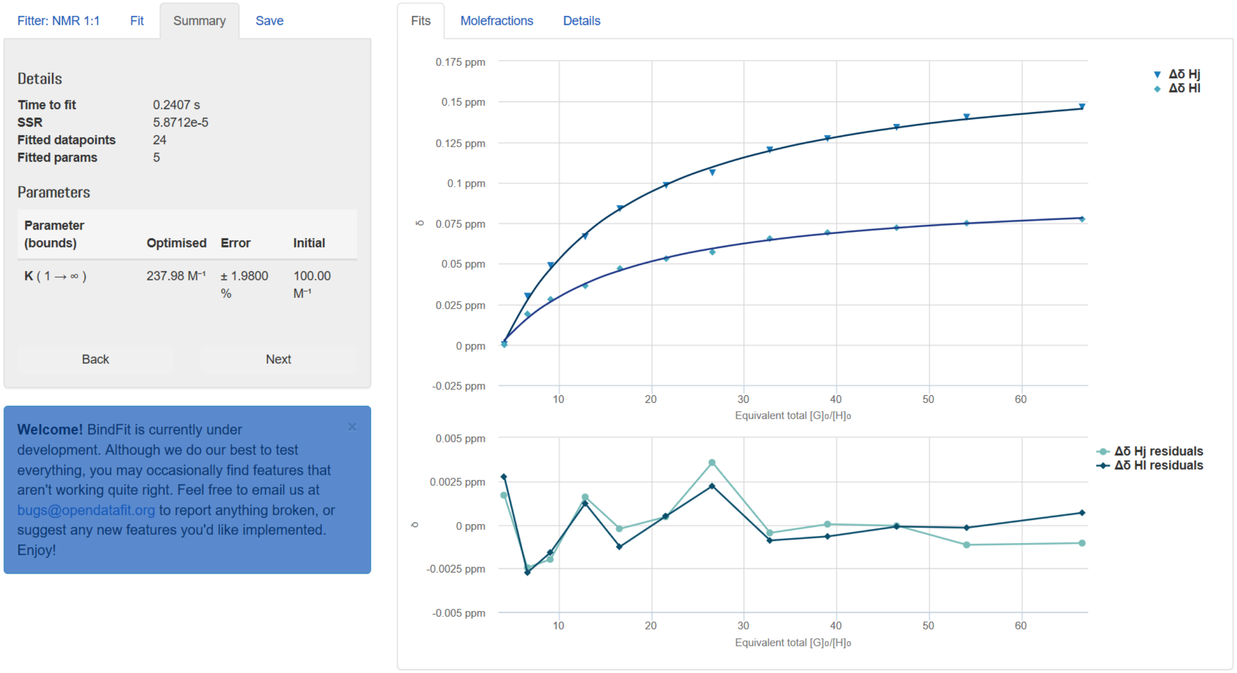

Figure S23. Fit of the data for H_j and H_l from Figure S22 to a 1:1 binding model, floating δ_H and starting with the first point at 4 equiv. Cl^- (up to 63 added equiv.). http://app.supramolecular.org/bindfit/view/a781e60f-9e2c-44a7-9a67-d0e53f47753a [4,5,8] (Bindfit and supramolecular.org are part of OpenDataFit.org and are open source & open access).

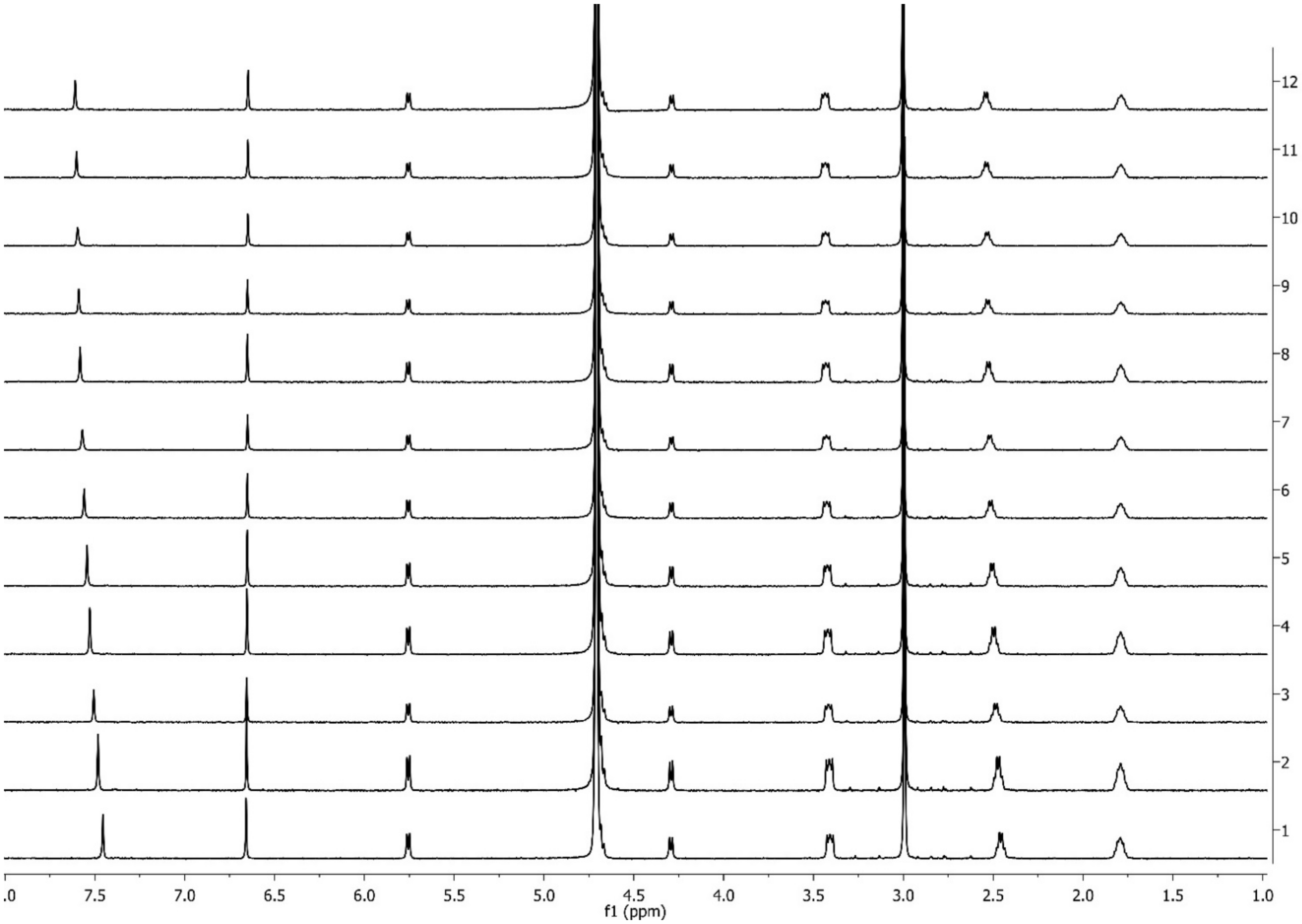

Figure S24. Representative titration of **1** with 250 mM KCl (up to ~62 added equiv.).

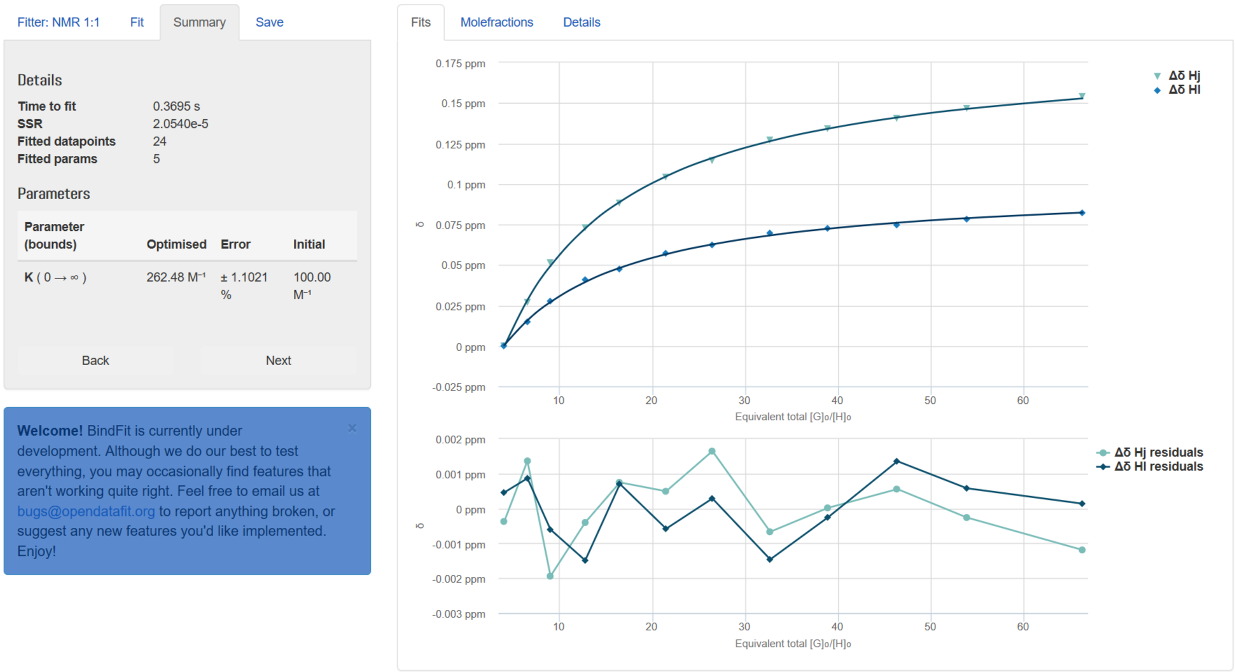

Figure S25. Fit of the data for H_j and H_l from Figure S24 to a 1:1 binding model, floating δ_H and starting with the first point at 4 equiv. Cl^- (up to 62 added equiv.). http://app.supramolecular.org/bindfit/view/57686487-42d2-4793-ad1d-fe394550f710 [4,5,8] (Bindfit and supramolecular.org are part of OpenDataFit.org and are open source & open access).

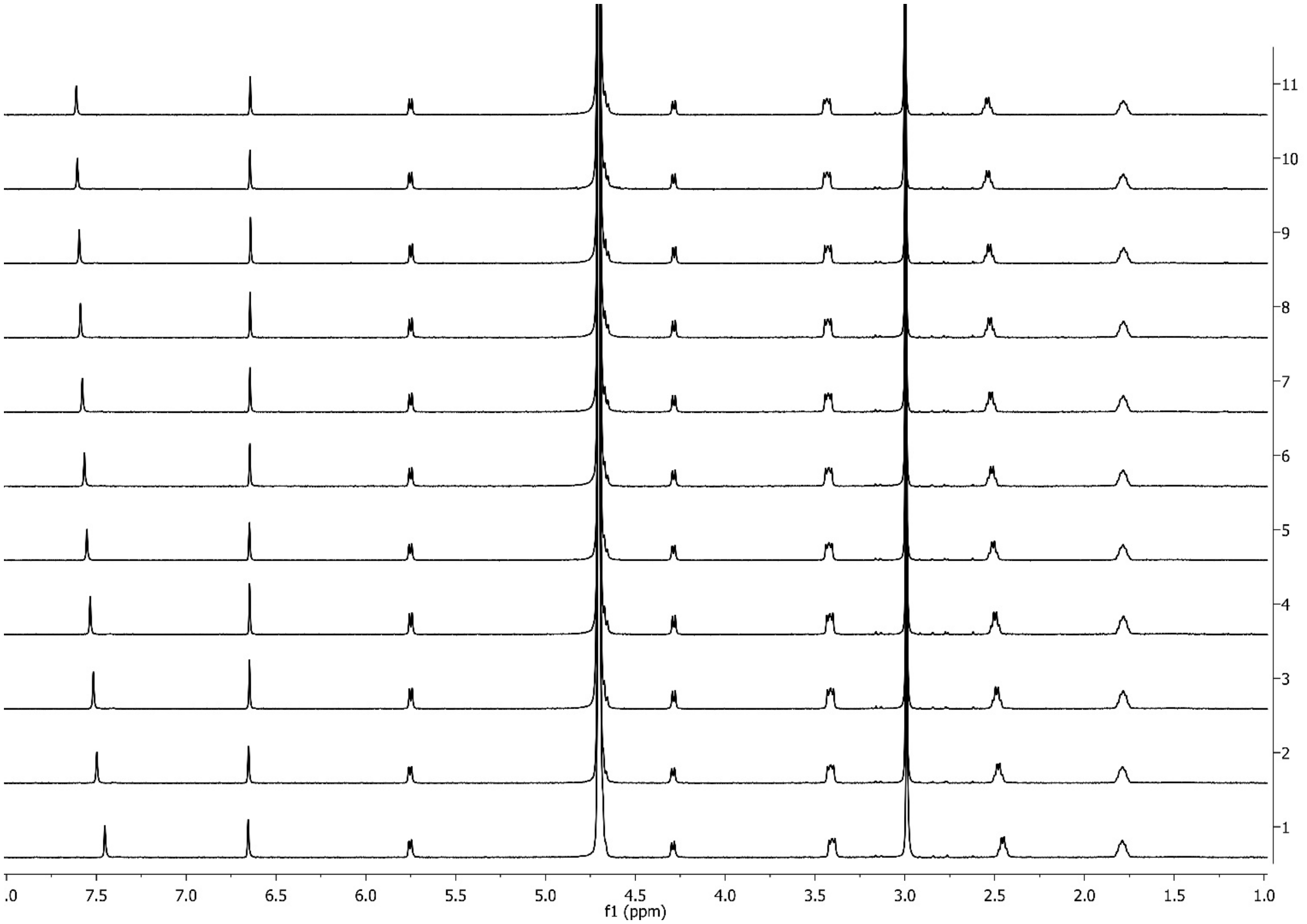

Figure S26. Representative titration of **1** with 250 mM CsCl (up to ~63 added equiv.).

.

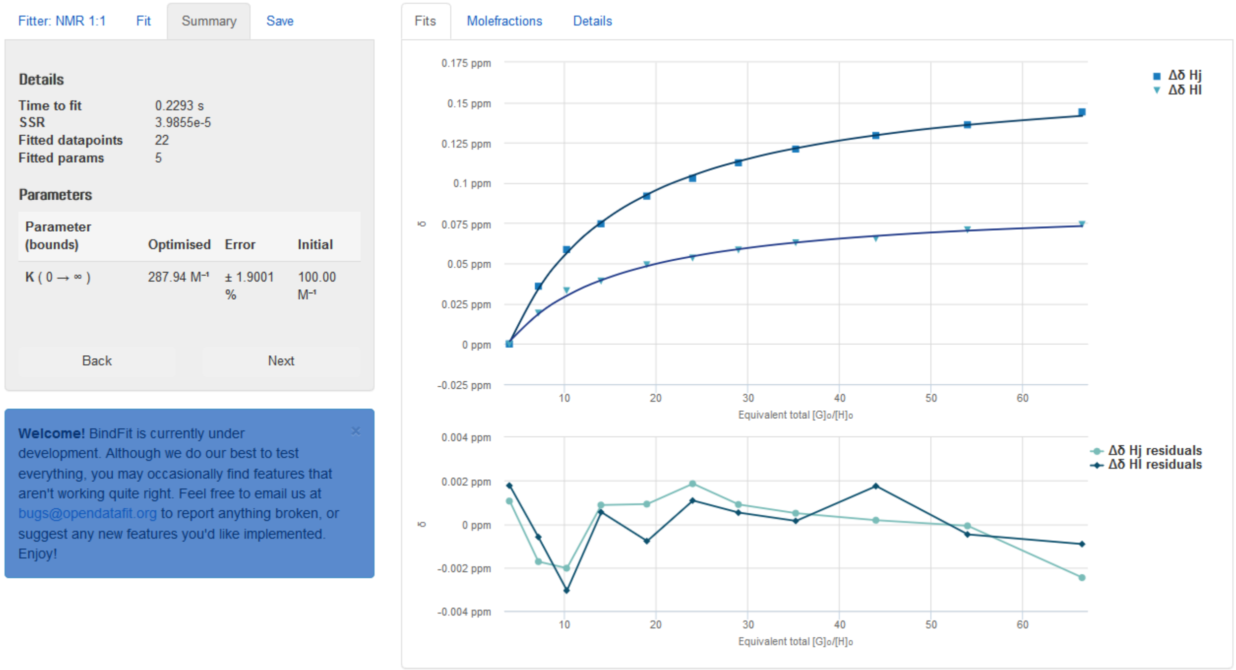

Figure S27. Fit of the data for H_j and H_l from Figure S26 to a 1:1 binding model, floating δ_H and starting with the first point at 4 equiv. Cl^- (up to 63 added equiv.). http://app.supramolecular.org/bindfit/view/02fd2355-d91f-4ad9-89b0-b698e19d0983 [4,5,8] (Bindfit and supramolecular.org are part of OpenDataFit.org and are open source & open access).

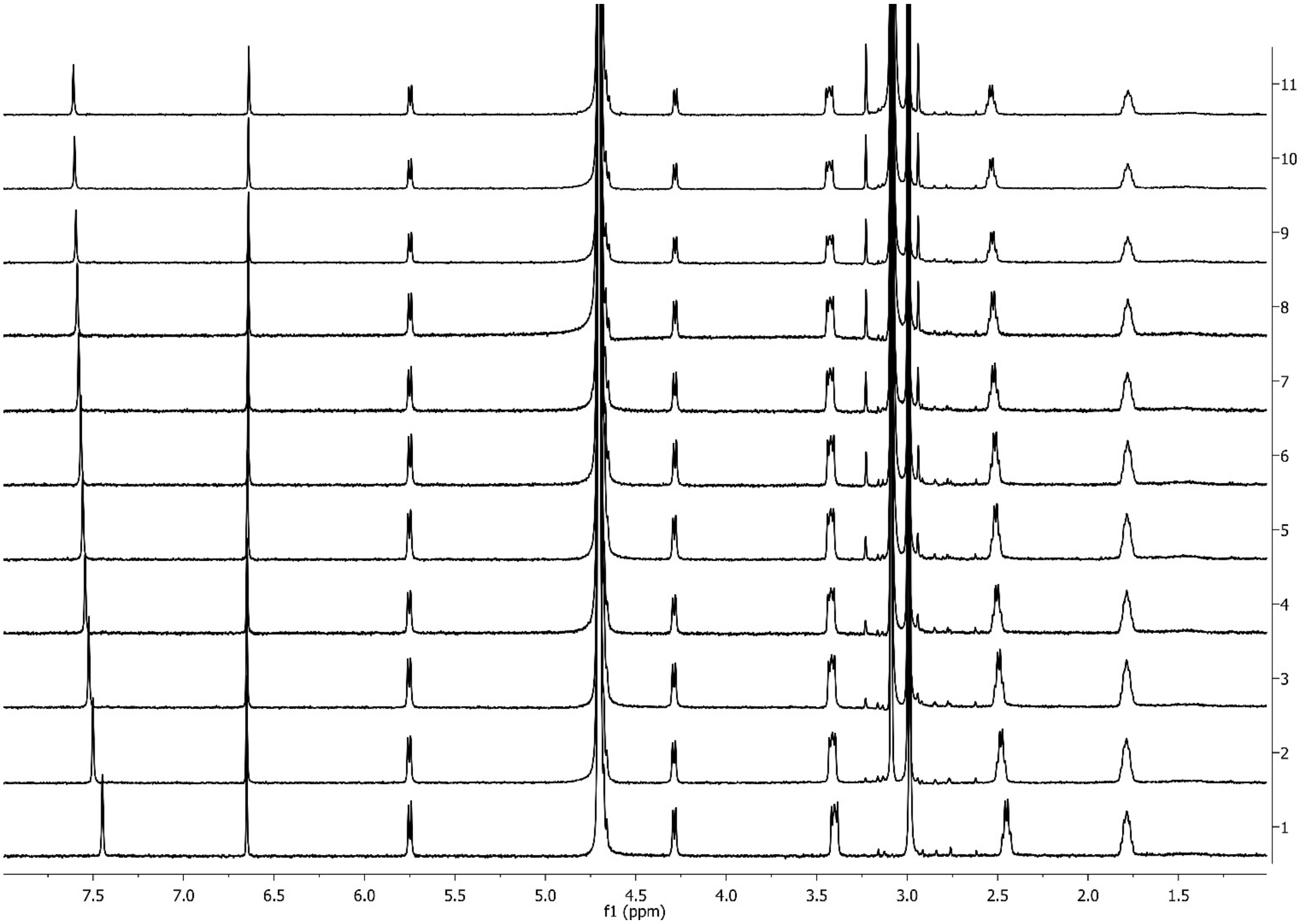

Figure S28. Representative titration of **1** with 250 mM $N(CH_3)_4Cl$ (up to ~63 added equiv.).

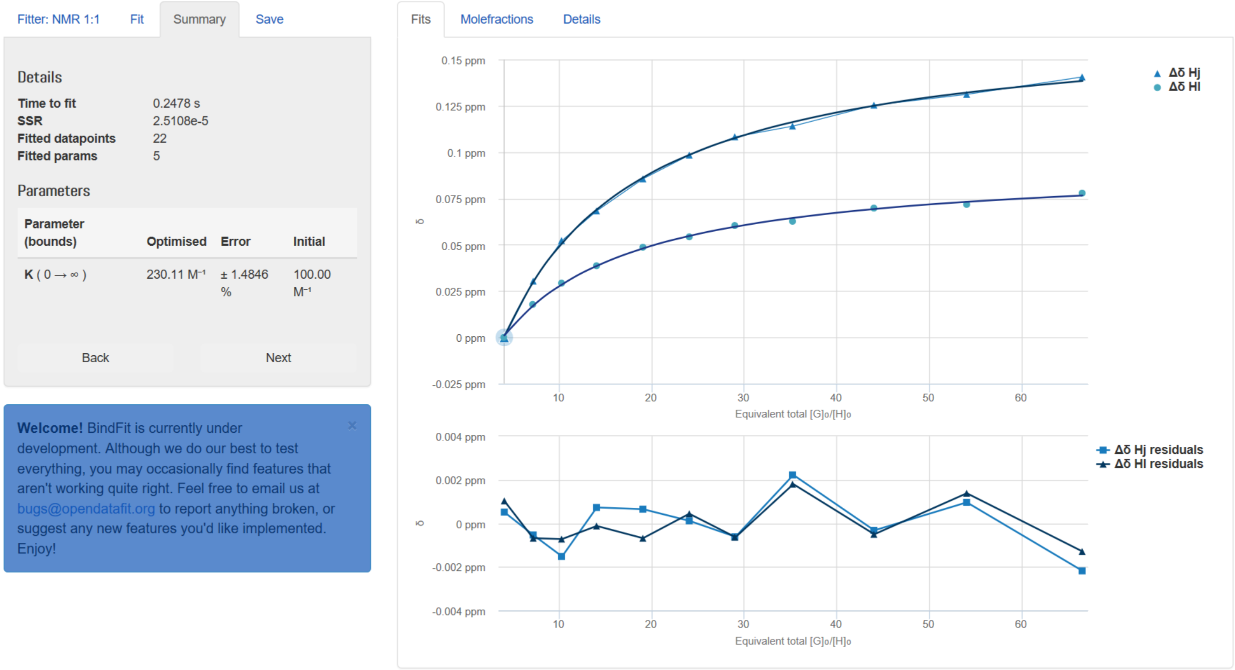

Figure S29. Fit of the data for H_j and H_l from Figure S28 to a 1:1 binding model, floating δ_H and starting with the first point at 4 equiv. Cl^- (up to 63 added equiv.). http://app.supramolecular.org/bindfit/view/fc97247d-c34f-4b0c-a216-e275d6e29058 [4,5,8] (Bindfit and supramolecular.org are part of OpenDataFit.org and are open source & open access).

The obtained values for $K_{Cl^-}^{U,0}$ and the corresponding free energy were plotted against thermodynamic parameters of the corresponding chloride salts and are shown graphically in Figure S30. The difference between the enthalpy or free energy of hydration of the respective cation and the chloride anion (Cl^-) were selected rather than individual physical or thermodynamic properties of the cations. In this scenario, interactions of the (solvated) cations with (solvated) Cl^- are related to observable changes in the experimental responses (K_a and ΔG), or rather the strongest affinity is observed when the counter-cation is Na^+, and the weakest with Li^+.

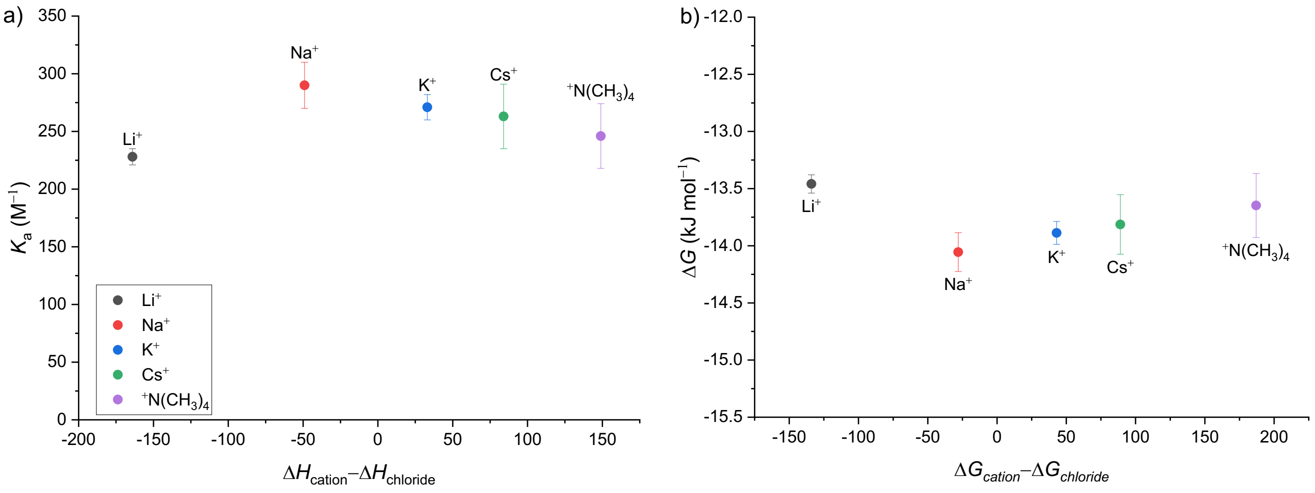

Figure S30. Experimentally determined $K_{Cl^-}^{U,0}$ and $\Delta G_{Cl^-}^{U,0}$ plotted against thermodynamic parameters of the salt. a) plot of the difference in the enthalpy of hydration of cation and chloride anion ($\Delta H_{cation} - \Delta H_{chloride}$) against the K_a value obtained for chloride binding to host **1** as the chloride salt of the cation; b) plot of the difference in the free energy of hydration of the cation and chloride anion ($\Delta G_{cation} - \Delta G_{chloride}$) against the ΔG value obtained for chloride binding to host **1** with the respective counter-cation. Error bars refer to absolute error from at least three measurements. Thermodynamic parameters of the cations and chloride obtained from reference [9].

c. Other halide affinity determinations to cavitand **1**

When determining the affinity of other halides to the chloride salt of host **1**, it needs to be noted that the titrating anion is in competition with the four intrinsic equivalents of chloride ions ($K_{Cl^-}^{U,0}$ = 290 M^{-1} determined using NaCl). In these experiments titration with bromide (Br^-), and iodide (I^-) salts generally lead to large signal shifts (δ_{max}) in H_j or H_l (Scheme S1) of the host. Smaller shifts were observed for fluoride (F^-). A representative titration and binding isotherm for host **1** titrated with each of the halides (unbuffered D_2O) is shown in Figure S31 – Figure S36.

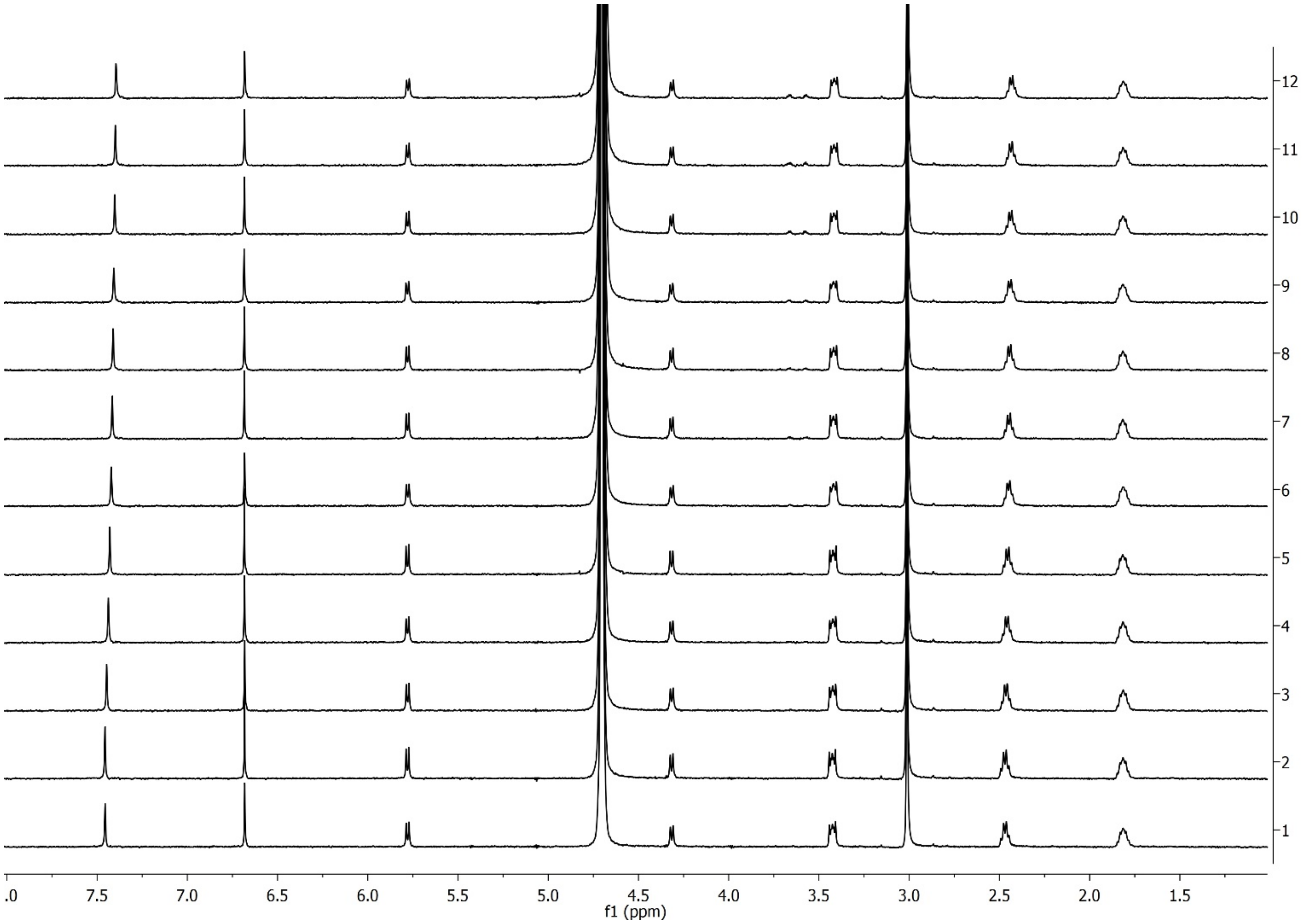

Figure S31. Representative ^{1}H NMR titration of **1** with 500 mM F^{-} up to 165 equiv. NaF.

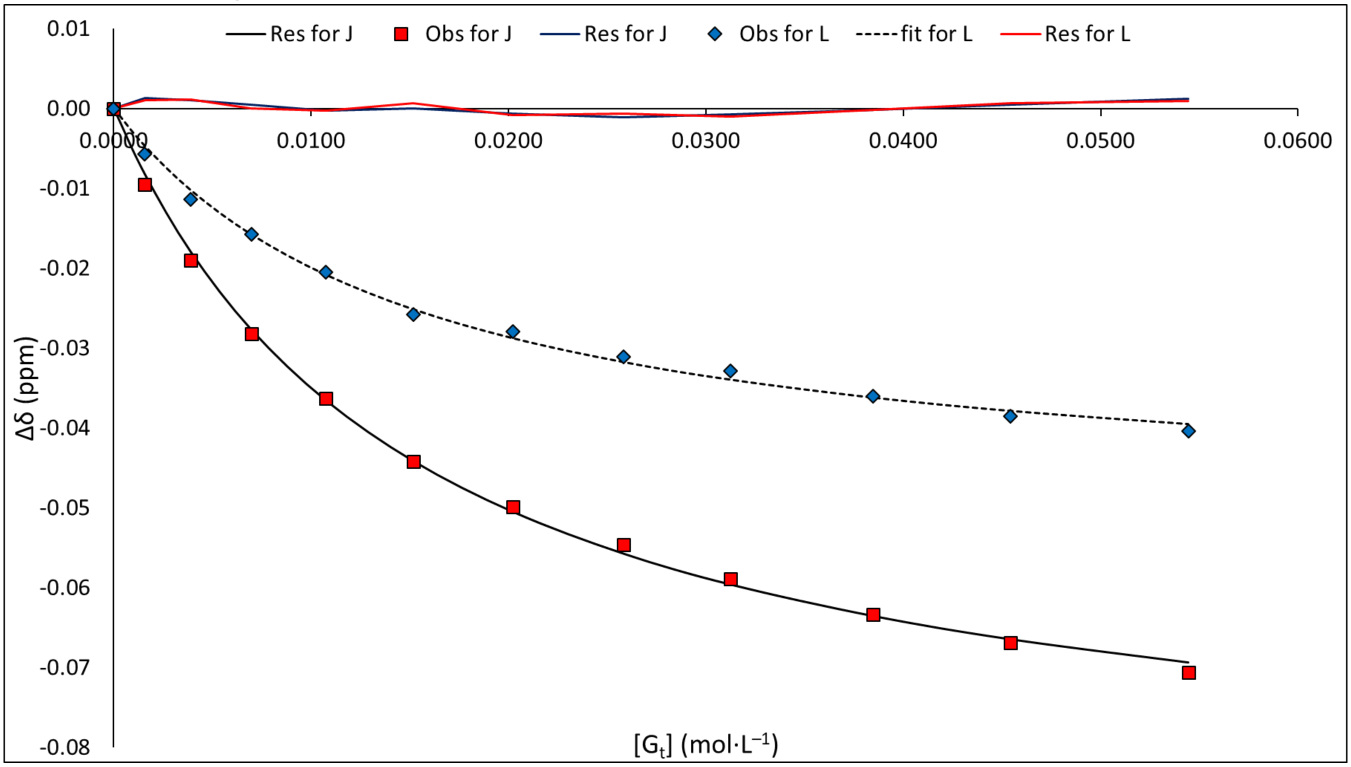

Figure S32. Fit of the data for H_j and H_l from Figure S31 to a 1:1 competitive binding model.

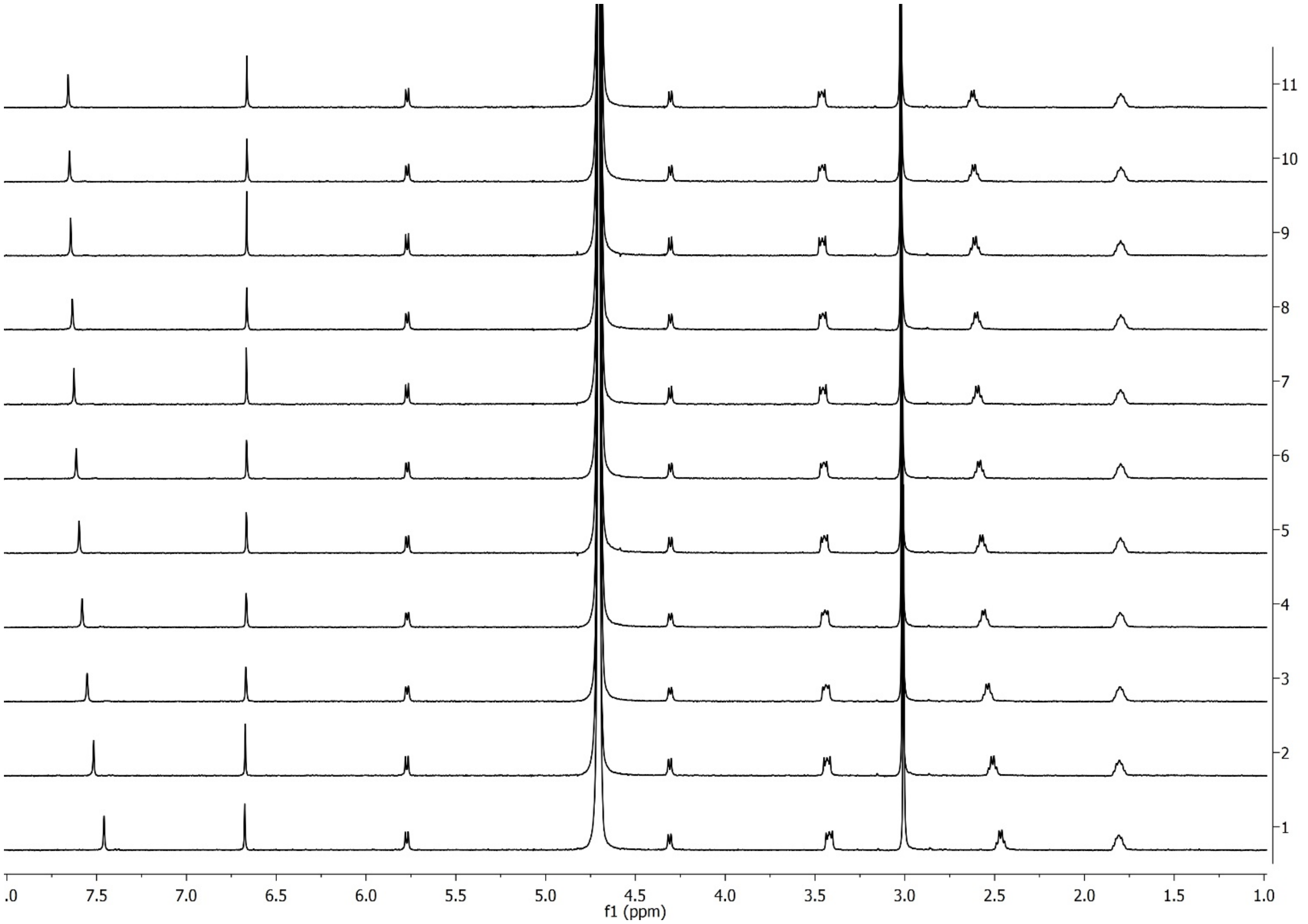

Figure S33. Representative ^{1}H NMR titration of **1** with 60 mM Br^- up to 15 equiv. NaBr.

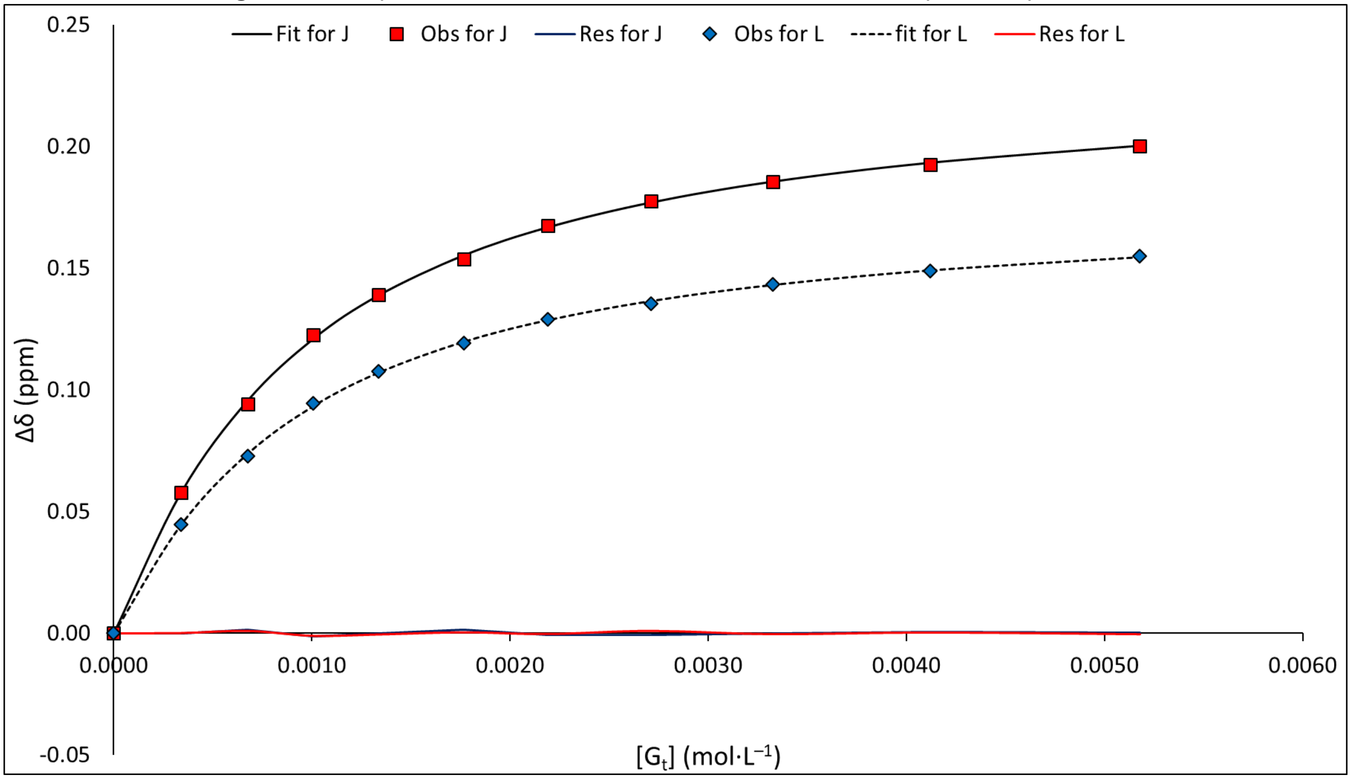

Figure S34. Fit of the data for H_j and H_l from Figure S33 to a 1:1 competitive binding model.

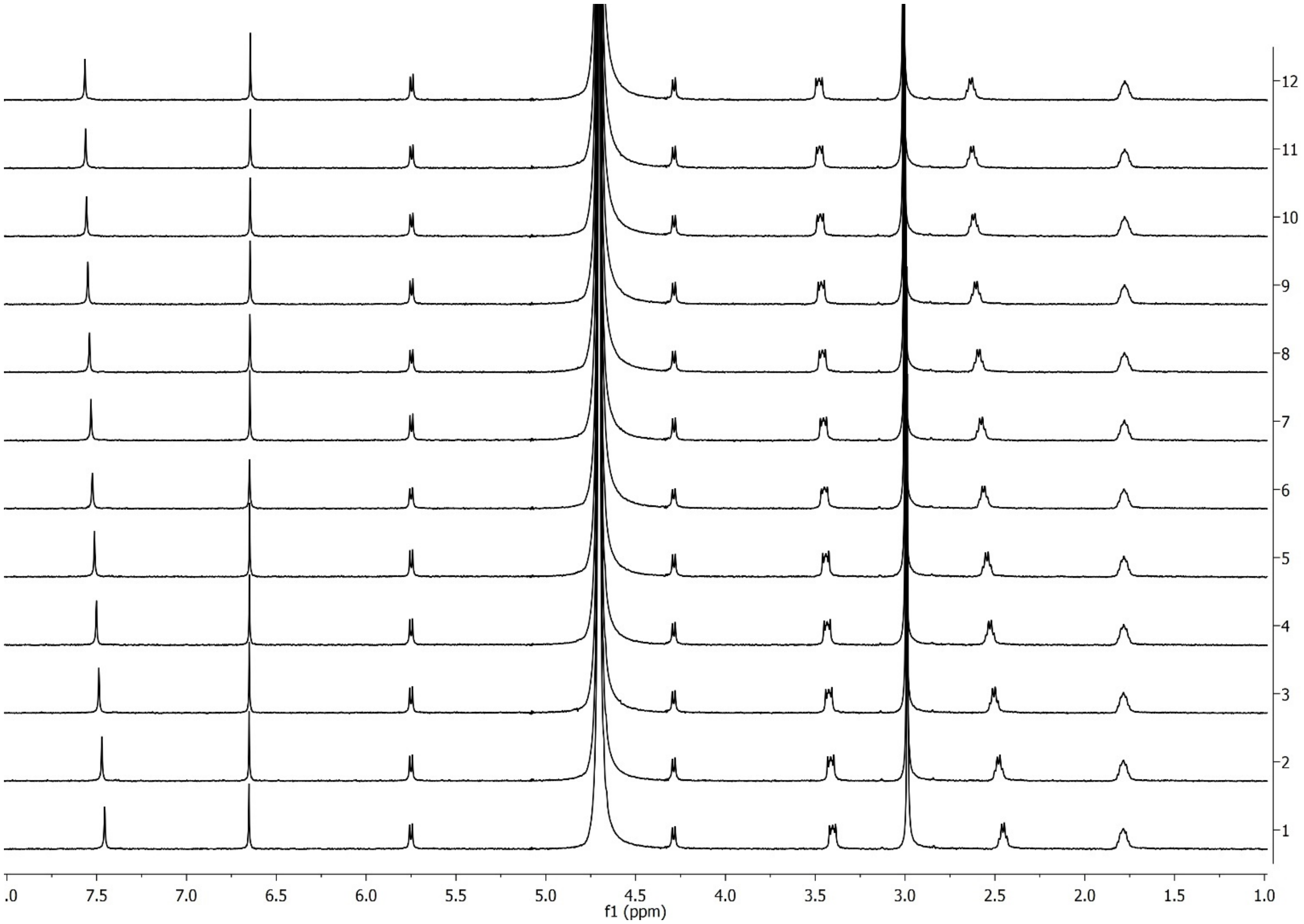

Figure S35. Representative ^{1}H NMR titration of **1** with 15 mM I^{-} up to 3.0 equiv. NaI.

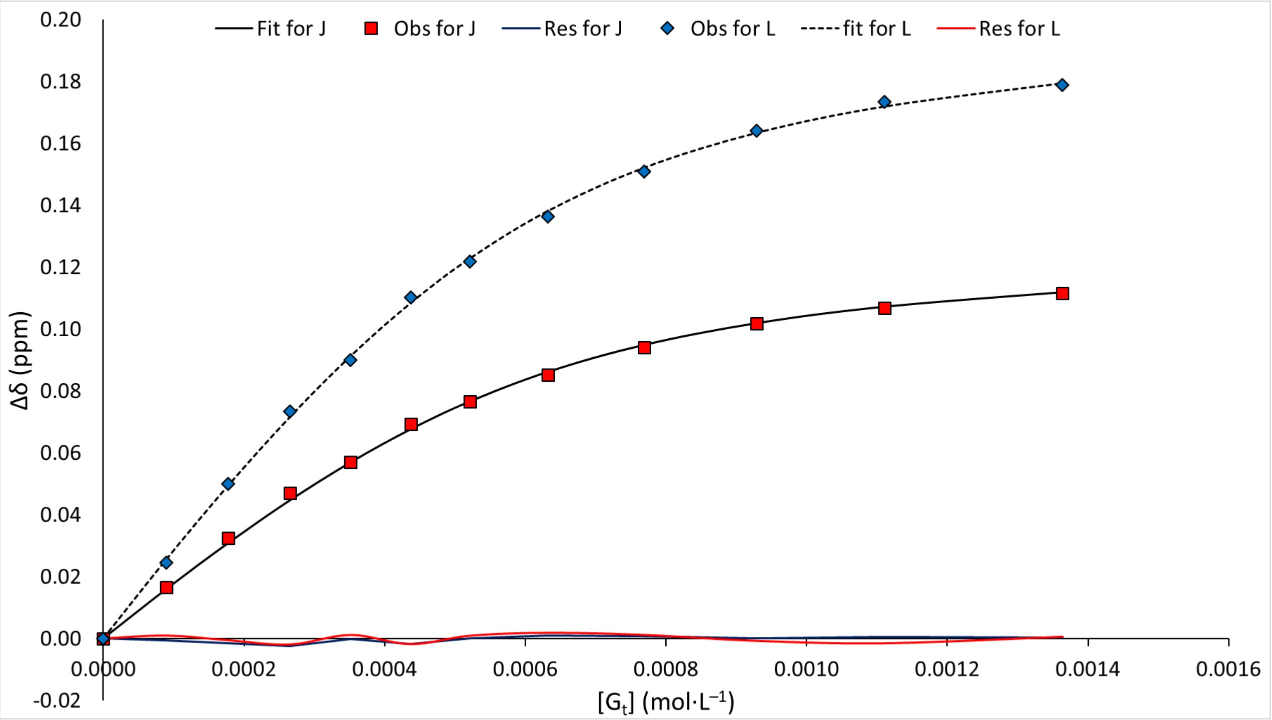

Figure S36. Fit of the data for H_j and H_l from Figure S35 to a 1:1 competitive binding model.

For a competing (titrating) guest (X^-) and the intrinsic chloride (Cl^-) the mass balance for the competitive complexation model is defined by the total host concentration ($[H_t]$) and the total concentration of each of the guests ($[X^-]_t$ and $[Cl^-]_t$), i.e., Eq. S16–S18:

$$[H]_t = [H] + [HCl^-] + [HX^-] \quad \text{(S16)}$$
$$[X^-]_t = [X^-] + [HX^-] \quad \text{(S17)}$$
$$[Cl^-]_t = [Cl^-] + [HCl^-] \quad \text{(S18)}$$

where $[HCl^-]$ and $[HX^-]$ are the concentrations of the host-chloride complex and host-(titrating)anion (guest) complex respectively, while $[Cl^-]$ and $[X^-]$ are the concentrations of the free chloride and titrating anion (guest). Substitution of the mass balance and the mole fraction definition (Eq. S15) into the definition of the equilibrium constant $K_{eq} = [HG]/[H][G]$, gives an expression that can be solved for the free host concentration $[H]$ in terms of the total concentrations $[X^-]_t$, $[H]_t$ and $[Cl^-]_t$, and the anion affinities of the guests K_{X-} and K_{Cl-}. This gives the cubic function Eq. S10.[10]

$$\alpha[H]^3 + \beta[H]^2 + \gamma[H] + \delta = 0 \quad \text{(S19)}$$

Where:
$$\alpha = K_{X-}K_{Cl-}$$
$$\beta = K_{Cl-} + K_{X-} + K_{Cl-}K_{X-}([X^-]_t + [Cl^-]_t - [H]_t)$$
$$\gamma = 1 + K_{Cl-}([Cl^-]_t - [H_t]) + K_{X-}([X]_t - [H]_t)$$
$$\delta = -[H]_t$$

The cubic function (Eq. S19) was solved trigonometrically for the smallest, real, positive number to give the free host concentration $[H]$. Thus, $[H]$ can be used to relate the concentration of free host to the total concentration of host and guest and was used in non-linear curve fitting by applying an equation defining the NMR binding isotherm.[11,12] This was used to determine K_{X-} for each of the halides based on $K_{Cl^-}^{U,0}$ = 290 M^{-1} for Cl^-. The data obtained ($K_{X^-}^{U,0}$, CV%, and ΔG) is summarized in Table S2.

Table S2: Summary of ^{1}H NMR titration data for binding constant determinations. K_a, ΔG, and error for the chloride salt of host **1** at ~0.4 mM concentration in unbuffered D_2O. The pD of the solutions was uncorrected.

Anion	$K_{X^-}^{U,0}$ (M^{-1})	CV%[c]	$\Delta G_{X^-}^{U,0}$ (kJ·mol^{-1})
F^-	104 ± 14 [b]	14	–11.49 ± 0.32
Cl^-	290 ± 20 [a]	7	–14.06 ± 0.17
Br^-	1860 ± 237 [b]	13	–18.64 ± 0.33
I^-	12,800 ± 1450 [b]	11	–23.43 ± 0.29

a) $K_{Cl^-}^{U,0}$ value obtained by accounting for four equiv. of Cl^- and floating initial point.
b) $K_{X^-}^{U,0}$ value obtained by competitive complexation model using $K_{Cl^-}^{U,0}$= 290 M^{-1}.
c) CV% obtained from at least three measurements.

To confirm the results of this method, the tetrabromide salt of host **1** was also prepared, and K_{Br-} determined by titrating with NaBr. Again, because there are four equivalents of (intrinsic) Br^- present at the start of the titration, the real initial point corresponding to the theoretical ^{1}H NMR signal from the bromide-free host (δ, ppm) is not known. Therefore, to determine the binding affinity of Br^- to the host, the initial observed point of the titration was set to correspond to four equivalents of bromide, and the true (theoretical) δ value for the initial point corresponding to zero equivalents of bromide allowed to float when solving for Δδ in Eq. S11. Titration of the tetrabromide host with NaBr (Figure S37 – Figure S38)

gave 1890 ± 254 M^{-1}, in very good agreement with the data obtained from titration of the tetra-chloride salt of **1** with Br^- (Table S2, $K^{U,0}_{Br^-}$ = 1860 ± 237 M^{-1}). This value for Br^- affinity was also used with the competitive complexation model (Eq. S19) to determine the affinity of iodide towards the tetra-bromide salt of **1** (Figure S39 – Figure S40). This gave K_{I^-} = 12,400 ± 1410 M^{-1}, again within statistical agreement from that obtained with the chloride salt (Table S2, $K^{U,0}_{I^-}$ = 12,800 ± 1450 M^{-1}). Changes in $\Delta\delta_{max}$ were too small under these conditions to accurately determine the affinity of F^- and Cl^- using the bromide salt of **1**.

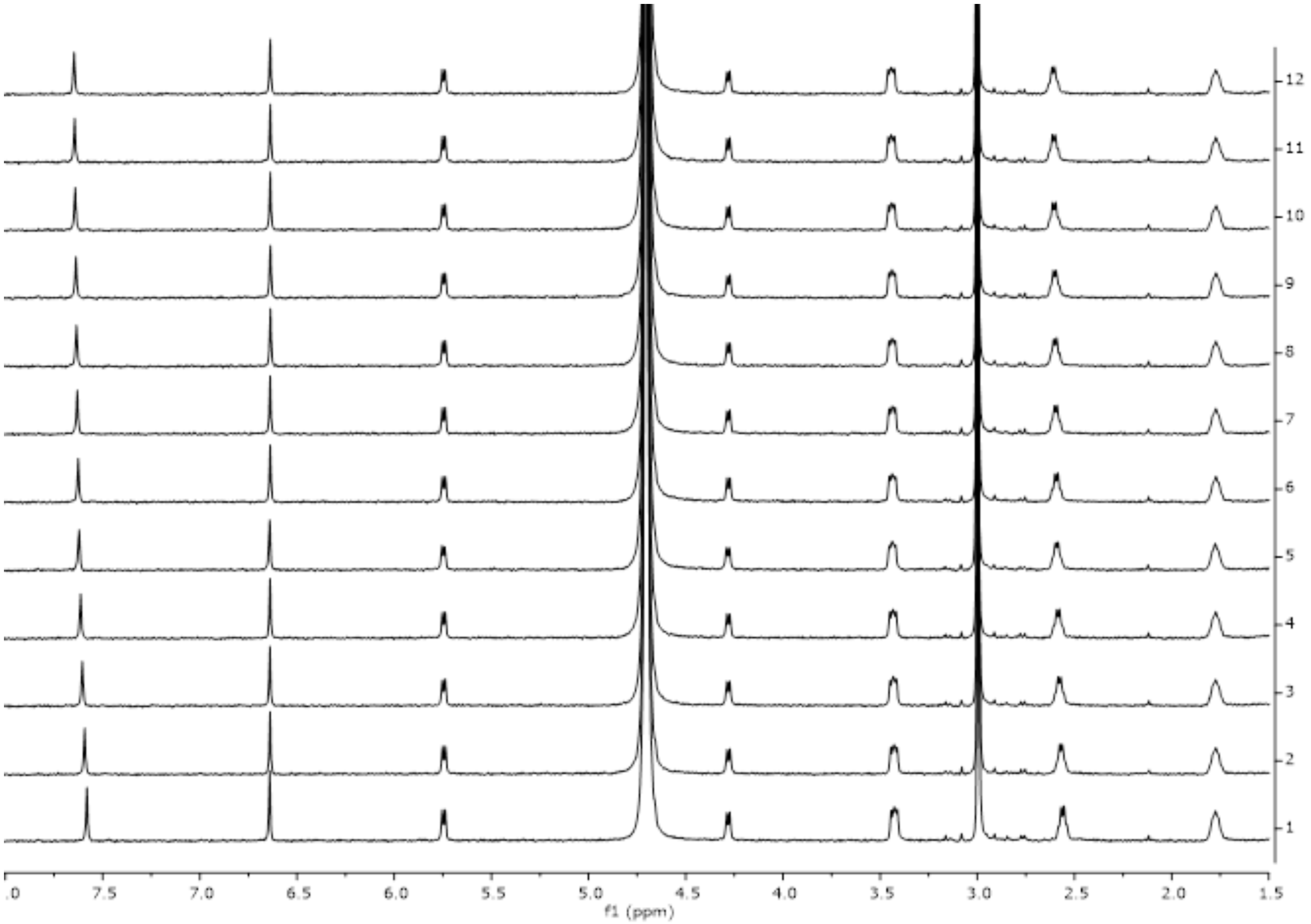

Figure S37. Representative ^{1}H NMR titration of **1** (counterion, Br^-) with 75 mM Br^- up to 22 equiv. NaBr.

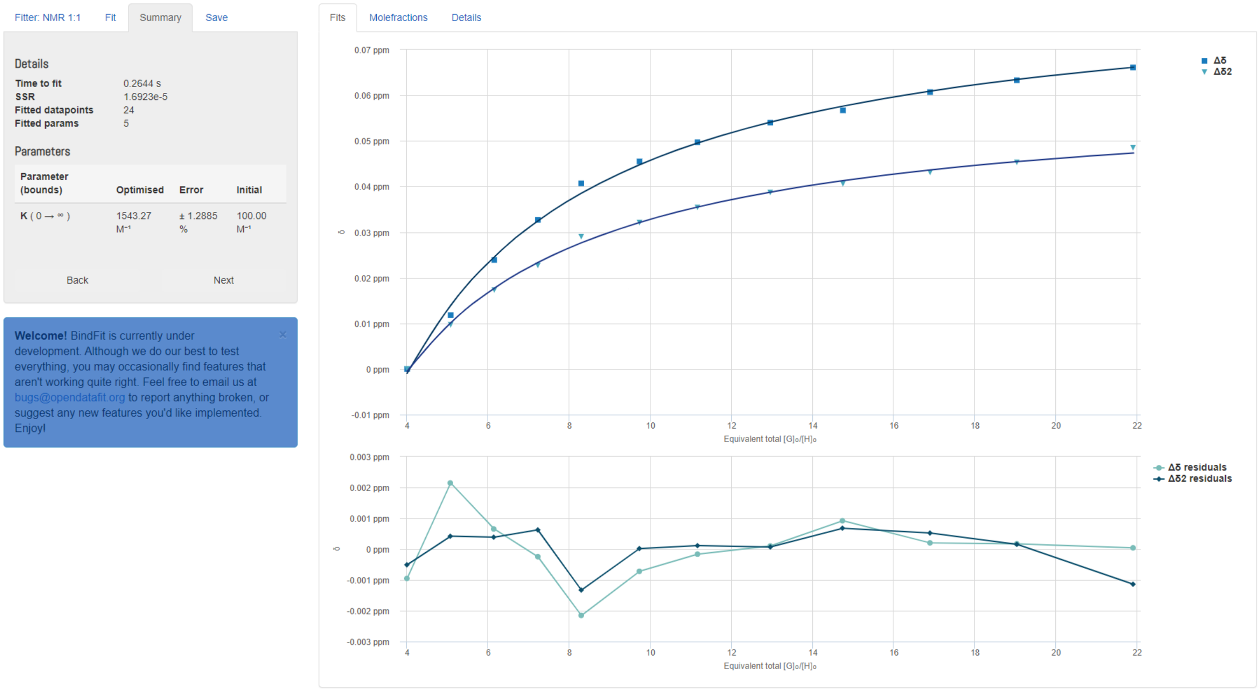

Figure S38. Fit of the data for H_j and H_l from Figure S37 to a 1:1 binding model, floating δ_H and starting with the first point at 4 equiv. Br⁻ (up to 18 added equiv.). http://app.supramolecular.org/bindfit/view/c34a4d30-87cb-4249-9d2d-a649e595bac4 [4,5,8] (Bindfit and supramolecular.org are part of OpenDataFit.org and are open source & open access).

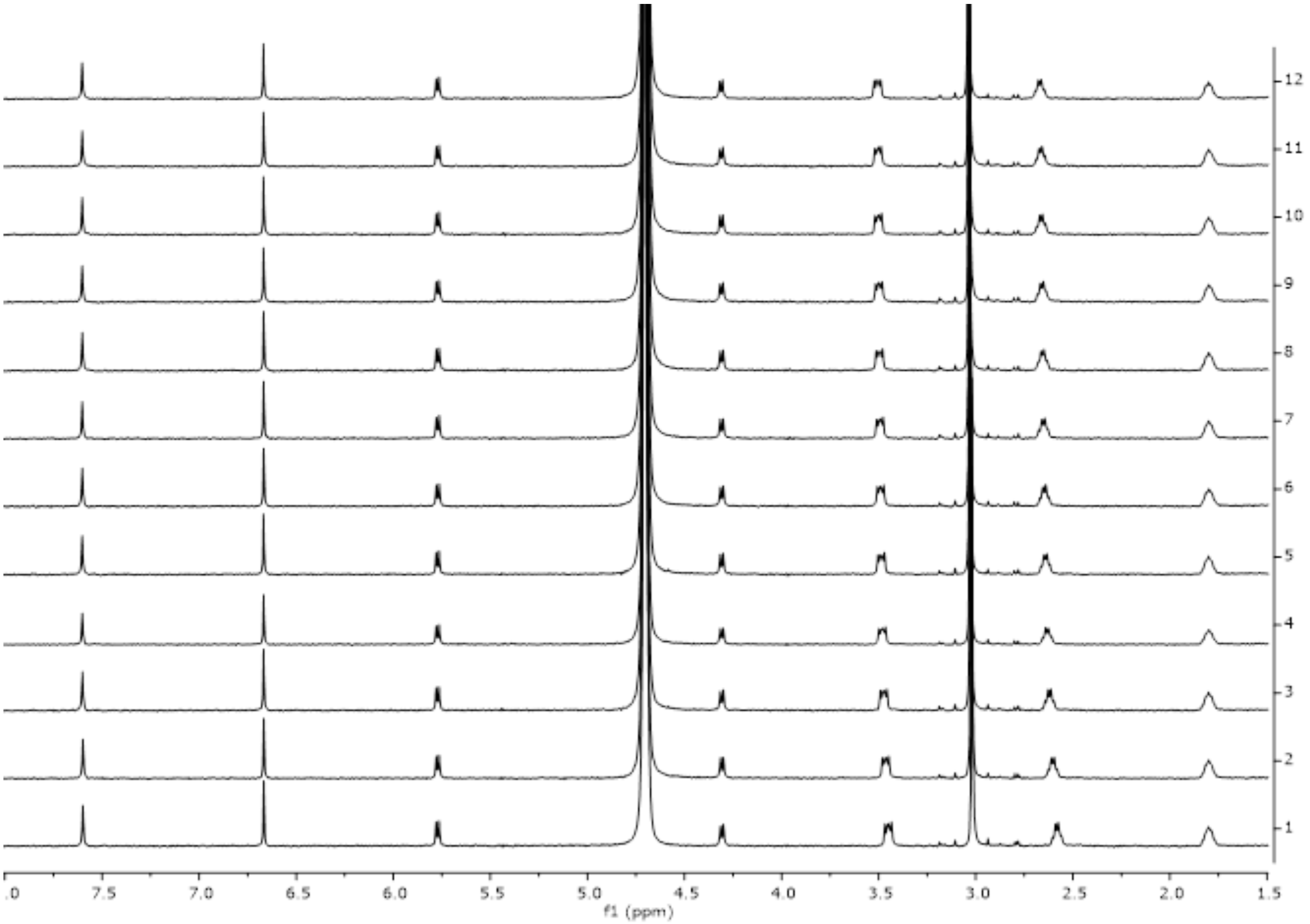

Figure S39. Representative ^{1}H NMR titration of **1** (counterion, Br⁻) with 23 mM I⁻ up to 6.0 equiv. NaI.

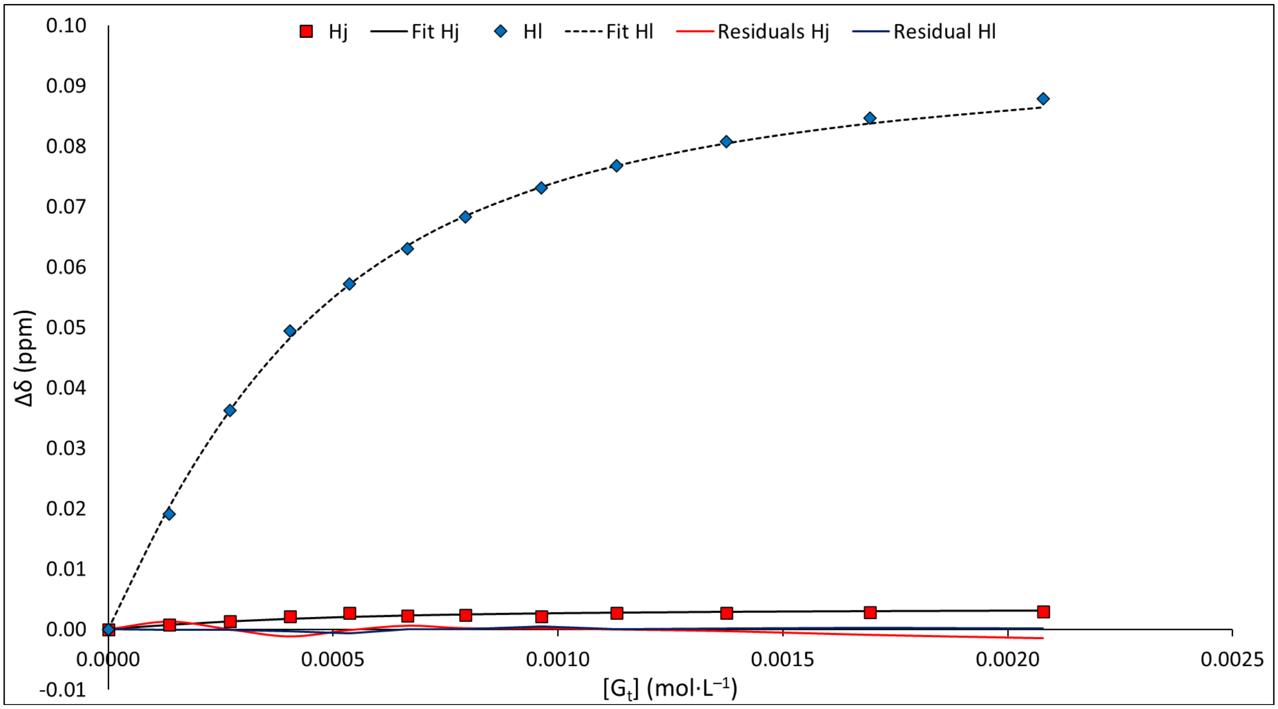

Figure S40. Fit of the data for H_j and H_l from Figure S39 to a 1:1 competitive binding model.

d. Buffer complexation determinations

Phosphate buffer is routinely used to prepare buffer solutions because it has three distinct regions in which the acid is in equilibrium with its conjugate base and therefore can provide efficient buffering capacity across several broad pH ranges. Nominally, they are pH: 0–4; 5–9; and 10–14 (Figure S41).

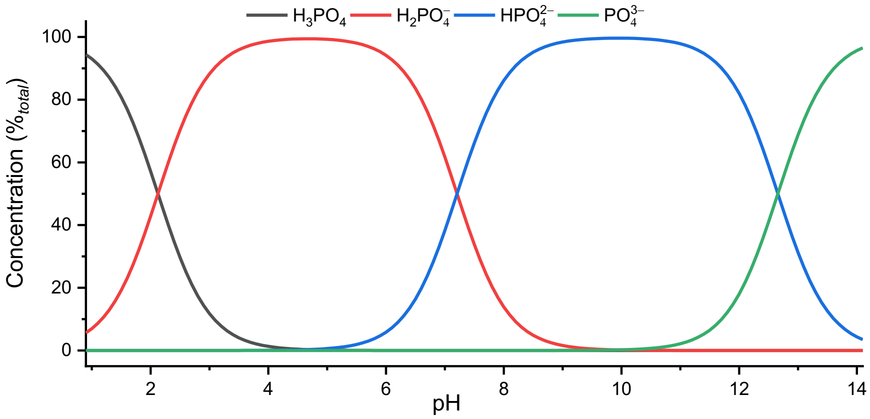

Figure S41. Phosphate speciation at different pH values.

The competitive complexation model (Eq. S19) was used to determine the affinity of hydrogen phosphate (HPO_4^{2-}) and dihydrogen phosphate ($H_2PO_4^-$) binding to the chloride salt of host **1**. Representative titrations (details given below) and isotherms for each anion are shown in Figure S42–Figure S45. Note that the HPO_4^{2-} and $H_2PO_4^-$ anions are the predominate phosphate species for the preparation of buffer solutions at acidic to slightly basic pH (e.g. biologically relevant buffers). Trivalent phosphate (PO_4^{3-}) was not investigated because the major species below pH = 12.7 is hydrogen phosphate (HPO_4^{2-}) and ^-OH; having four major species in solution precludes an accurate determination of the affinity of PO_4^{3-} using Eq. S19.

Note that solutions of HPO_4^{2-} inevitably contain varying amounts of $H_2PO_4^-$ and ^-OH (from the reaction of HPO_4^{2-} with water). During the titration with HPO_4^{2-}, the pD varied from ~8.4 (after the first aliquot of salt) to ~9.6. Thus, over this range the [^-OH] was ~0.003-0.04 mM (<0.01–0.1 mol%), and the concentration of HPO_4^{2-} varied from 94 to >99%. As a result, the data from this titration fitted the competitive complexation model (intrinsic Cl^- and HPO_4^{2-} in competition for host) well. The corresponding titration with $H_2PO_4^-$ falls within a narrower range for anion-speciation. In this titration the pH varied from ~5.5 to ~4.6 and thus, the concentration of $H_2PO_4^-$ was always >98%. Thus, within this range the hydronium ion concentration is both negligible (0.003–0.03 mM) and irrelevant to binding. Again, the data fitted the competitive complexation model well.

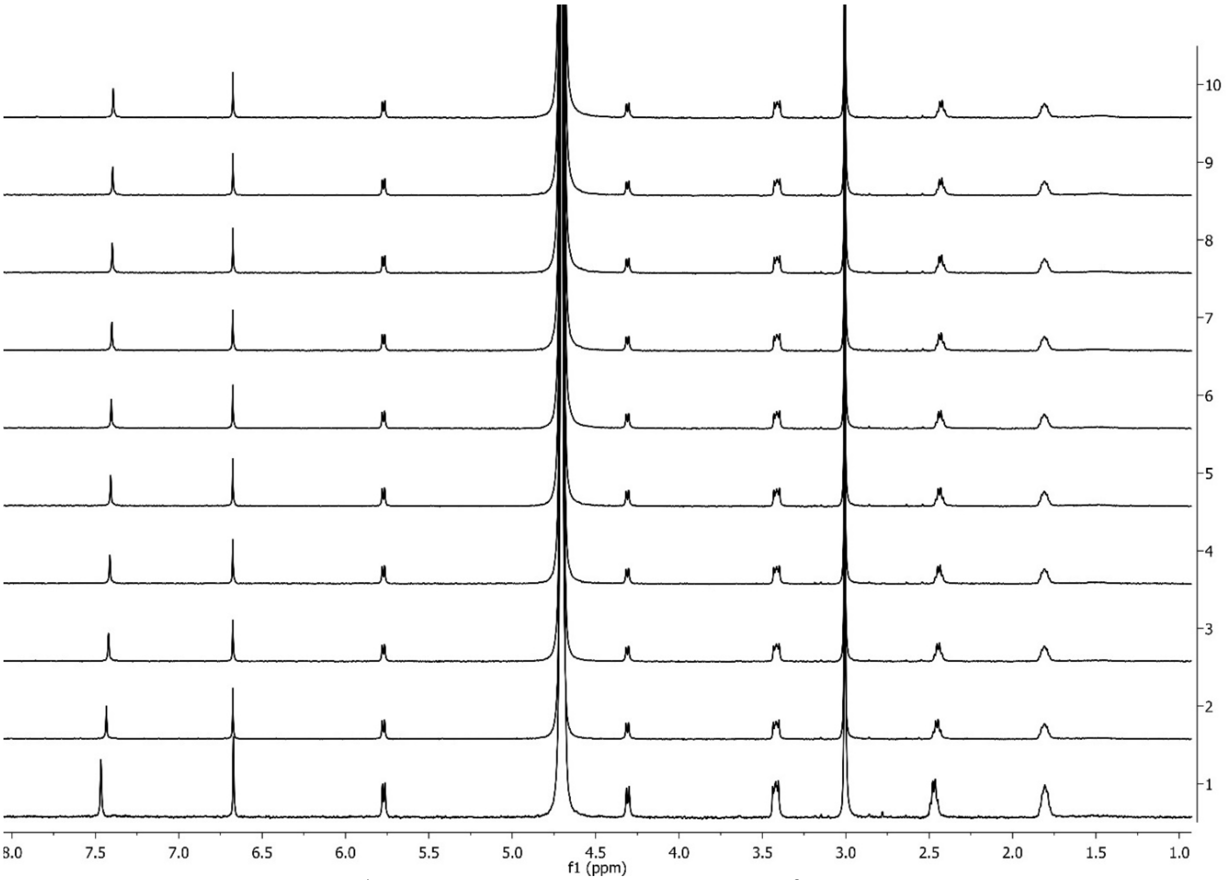

Figure S42. Representative ^1H NMR titration of **1** with 250 mM HPO_4^{2-} up to 62.5 equiv. of the sodium salt.

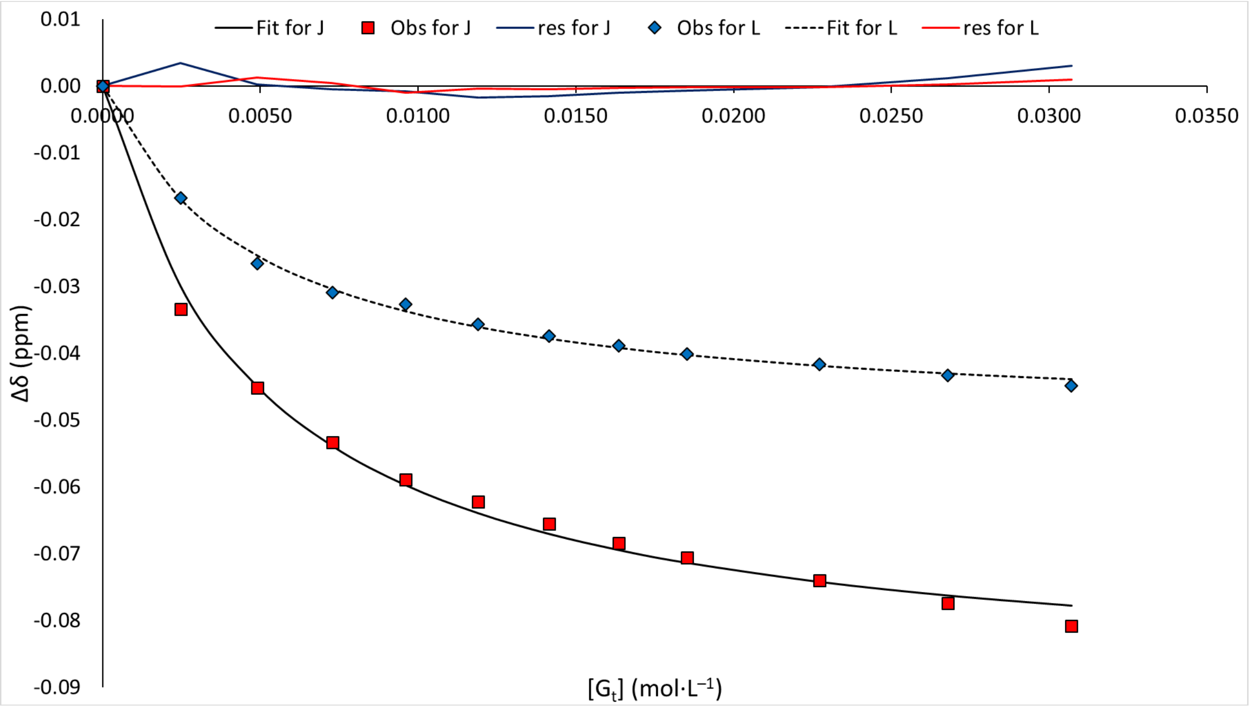

Figure S43. Fit of the data for H_j and H_l from Figure S42 to a 1:1 competitive binding model.

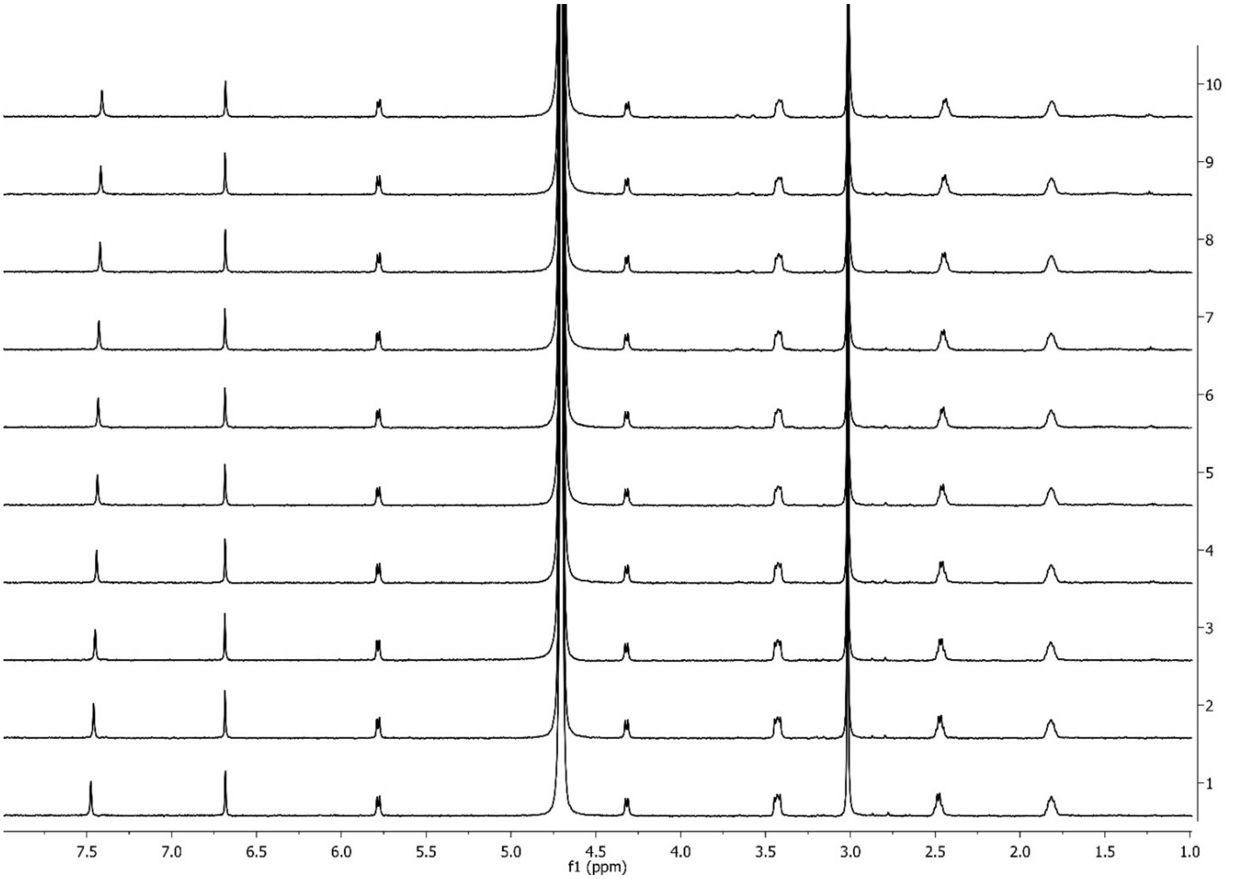

Figure S44. Representative ^1H NMR titration of **1** with 504 mM $H_2PO_4^-$ up to 126 equiv. of the sodium salt.

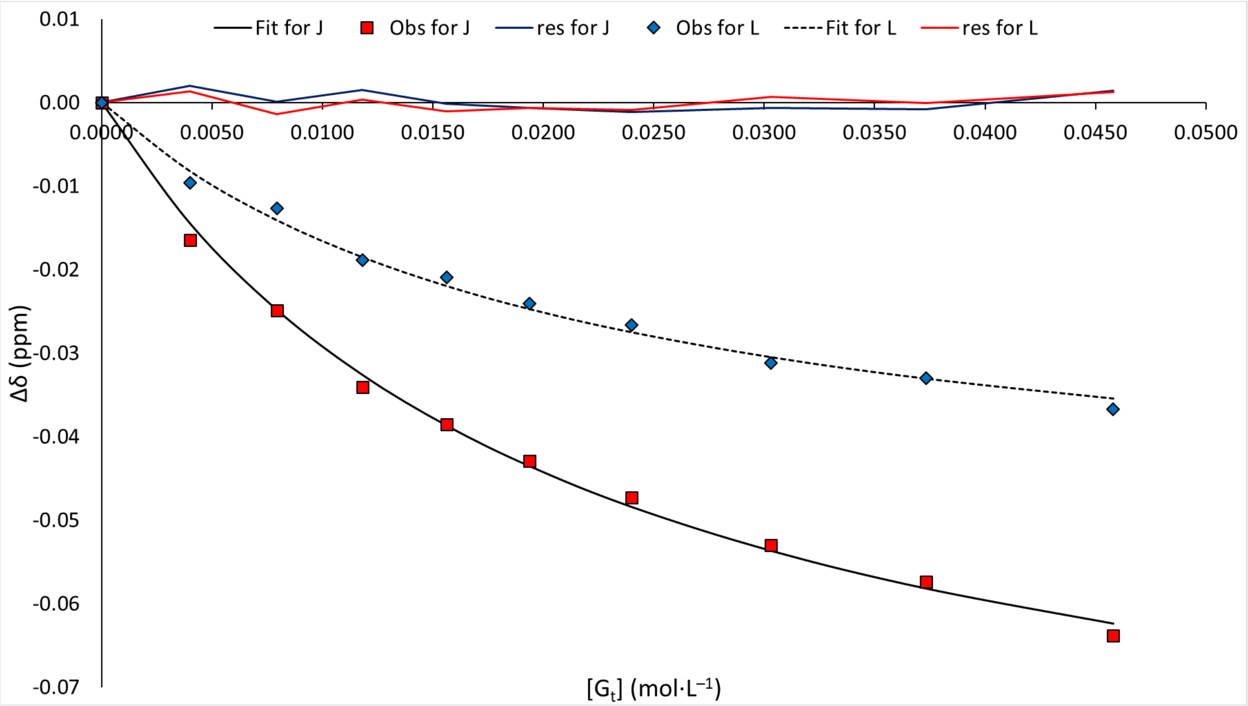

Figure S45. Fit of the data for H_j and H_l from Figure S44 to a 1:1 competitive binding model.

Fitting the data from the titration of the host with the corresponding sodium phosphate salts to the competitive model gave the association constants shown in Table S3.

Table S3: Binding of sodium phosphate salt anions to host **1** (0.4 mM, D_2O). The pD of the solutions was uncorrected.

Guest	$K_{X^-}^{U,0}$ (M^{-1})^a	CV%^{b}	*ΔG* (kJ·mol^{-1})
$H_2PO_4^-$	72 ± 7	9	–10.60 ± 0.22
HPO_4^{2-}	302 ± 31	10	–14.16 ± 0.25

a) $K_{X^-}^{U,0}$ value obtained by competitive complexation model using K_{Cl^-} = 290 M^{-1} for chloride.
b) CV% obtained from at least three measurements.

e. Determination of binding constants in buffered systems

To determine if the buffers attenuate the affinity of the halide guests, halide ion affinity determinations using **1** were performed under three different buffered solutions: 1) 10 mM phosphate pH 7.3 (45% $H_2PO_4^-$, 55% HPO_4^{2-}, *I* = 21.0 mM); 2) 23.8 mM phosphate buffer at pH 3.0 (12% H_3PO_4, 88% $H_2PO_4^-$, *I* = 21.0 mM) at same ionic strength of 1), and; 3) 10 mM phosphate buffer at pH = 3.0 (12% H_3PO_4, 88% $H_2PO_4^-$, *I* = 8.8 mM) maintaining the buffer concentration of 1), but at reduced ionic strength. In the latter two cases, neutral H_3PO_4 was assumed not to bind to the cationic host. The average initial and final *I* value for each titration is shown in Table S4.

Table S4: Initial and final ionic strength (*I*) for titrations of host **1** (0.4 mM) in three buffered conditions.[a]

Guest	10 mM pH = 7.3	23.8 mM pH = 3.0	10 mM pH = 3.0
I^-	22.6 | 25.5	22.6 | 24.3	10.4 | 12.5
Br^-	22.6 | 29.9	22.6 | 30.6	10.4 | 17.3
Cl^-	22.6 | 57.2	22.6 | 49.0	10.4 | 37.2
F^-	22.6 | 94.0 [b]	— [c]	— [c]

a) Ionic strength value reported as (*initial* | *final*) and includes contributions from the host, counterions, buffer, and guest.
b) although the titration was performed up to this value the measured binding was too weak to determine accurately.
c) Not determined.

Table S5 shows the observed binding constants ($K_{obs}^{U,0}$), obtained when the host **1**.4X⁻ was titrated with NaX in the three different solution conditions We highlight one example here with the data for Case 1 shown below for the titration of NaCl into a solution in **1**.4Cl⁻ in 10 mM phosphate buffer, pH = 7.3. The $K_{obs}^{U,0}$ for chloride was found to be 135 ± 3 M^{-1} (Figure S46 & Figure S47). In this case (titration of **1**.4Cl⁻ with Cl⁻), the affinity constant was attained by fitting the data to a 1:1 model and floating the initial point. For the remaining halides the titration data was fit with Eq. S19 using the obtained $K_{obs}^{U,0}$ for Cl⁻ under each set of conditions, respectively 135, 143 and 166 M^{-1} for 10.0 mM pH 7.3, 23.8 mM pH 3.0, and 10.0 mM pH 3.0. For validation, the tetrabromide salt of host **1** was also used to determine $K_{obs}^{U,0}$ for Br⁻ under case 1, using Eq. S11 and floating the initial point (See Section 4.A.c). This method (titration of the bromide salt of **1** (**1.**4Br⁻) with NaBr) gave $K_{obs}^{U,0} = 708$ ± 26 M^{-1} in very good agreement to the data obtained titrating the chloride salt of **1** (**1**.4Cl⁻), where $K_{obs}^{U,0} = 738$ ± 27 M^{-1}.

Table S5: Observed binding constants ($K_{obs}^{U,0}$) for the binding of halides to host **1**.4Cl– (0.4 mM) in three solution conditions.[a]

Guest	$K_{obs}^{U,0}$ (M^{-1}) to host 1.4Cl⁻		
	10 mM pH = 7.3[b]	23.8 mM pH = 3.0[c]	10 mM pH = 3.0[d]
I⁻	5430 ± 324 [e]	6000 ± 489 [e]	7410 ± 314 [e]
Br⁻	738 ± 27 [e]	862 ± 48 [e]	1020 ± 51 [e]
Cl⁻	135 ± 3 [f]	143 ± 8 [f]	166 ± 6 [f]
F⁻	—[g]	—[h]	—[h]

a) Average values base on at least three determinations.
b) 10 mM sodium phosphate buffer, pH 7.3 (*I* = 21 mM)
c) 23.8 mM sodium phosphate buffer, pH 3.0 (*I* = 21 mM)
d) 10 mM phosphate buffer, pH = 3.0 (*I* = 8.8 mM)
e) $K_{obs}^{U,0}$ value for X⁻ obtained by competitive complexation model (Eq. S19) using $K_{obs}^{U,0}$ for Cl⁻.
f) $K_{obs}^{U,0}$ value obtained by accounting for four equiv. of Cl⁻ and floating initial point.
g) The measured binding was too weak to determine accurately
h) Not determined.

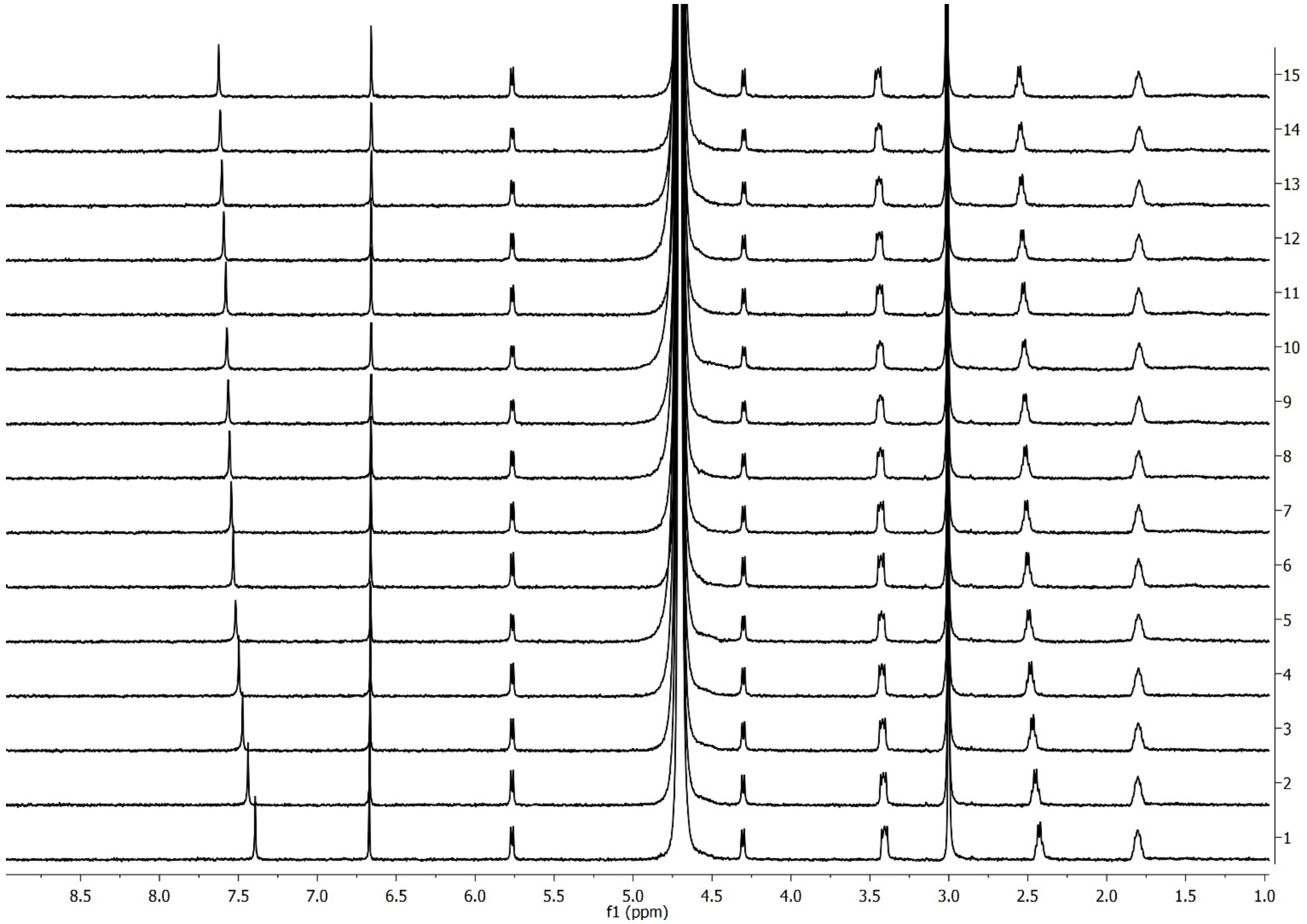

Figure S46. Representative titration of **1** (counterion, Cl^-) with 300 mM NaCl (10 mM phosphate buffer, pH = 7.3) (up to ~110 equivalents).

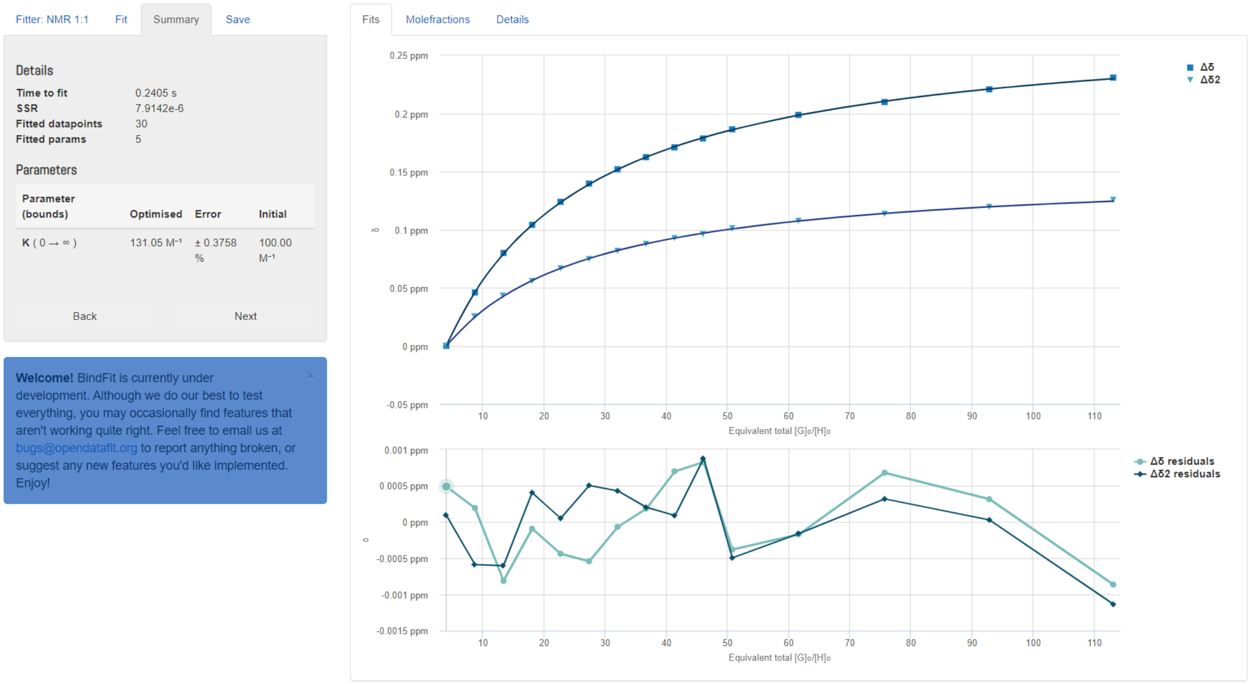

Figure S47. Fit of the data for H_j and H_l from Figure S46 to a 1:1 binding model floating δ_H and starting with the first point at 4 equiv. Cl^-. [4,5,8] http://app.supramolecular.org/bindfit/view/1869617a-c712-430b-a4a9-cff38cf1efd5 (Bindfit and supramolecular.org are part of OpenDataFit.org and are open source & open access).

f. Predicting binding constants in complex mixtures

Determining $K_{X^-}^{U,0}$ values in a straightforward competition system, for example of a halide or buffer to the host (sections c. and d.), involves a cubic equation that can be solved trigonometrically (Eq. S19). However, this is not the case when dealing with a more complex system, such as determining the binding constants for each species when the host is in a two-component buffer, or when titrating the host with a (second) halide in the presence of a one-component buffer. In these situations, the base mathematics is a quartic equation (or higher polynomial for more complex situations still) and it is not usually possible to determine each $K_{X^-}^{U,0}$ value in question *de novo*, either trigonometrically or by iterative fitting. However, as we show here, when selected association constants are known, it is possible to calculate/predict guest affinities ($K_{pred}^{U,0}$) in such complex systems. This ability to predict informs us that the mathematics used – which does not consider screening effects etc. – is sufficient for determining binding constants.

To demonstrate this, we predicted binding constants for a guest ($K_{pred}^{U,0}$) under complex buffer conditions and then compare these values to actual binding constants determined under those particular conditions of study ($K_{obs}^{U,0}$, Table S5). To determine $K_{pred}^{U,0}$ we again solve for the free host concentration $[H]$, where $[H] = [H]_t - [HG]$. From the definition of the equilibrium constant (K_{eq}) for any guest, G, $[HG] = K_G[H][G]$, thus $[H] = [H]_t - K_G[H][G]$, which in turn gives: $[H] = [H]_t/(1 + K_G[G])$. The free host concentration can then be solved in terms of the total host concentration, $[H]_t$, and the free guest concentration, $[G]$, and its affinity, K_G. Importantly, this open-form solution can be expanded to accommodate any number of guests.

Take, by way of example, the titration of any halide X^- into a solution containing the tetrachloride salt of host **1** (H^{4+}) in phosphate buffer (pH = 7.3). Let Y^- and Z^{2-} stand for the acid ($H_2PO_4^-$) and its conjugate base (HPO_4^{2-}) portions of the buffer. The respective concentrations of the free species and the concentrations of the host-acid and host-base complexes are $[Y^-]$, $[Z^{2-}]$, $[HY^{3+}]$, and $[HZ^{3+}]$. The mass balance for the free host concentration is: $[H^{4+}] = [H^{4+}]_t - [HCl^{3+}] - [HX^{3+}] - [HY^{3+}] - [HZ^{2+}]$. Since $[HG] = K_G[H][G]$, and there are equivalent expressions for $[HCl^{3+}]$, $[HX^{3+}]$, $[HY^{3+}]$, and $[HZ^{2+}]$, respectively, an expression can be derived for the free host concentration, Eq. S20 (corresponding to Eq. 15 in the main text):

$$[H^{4+}] = \frac{[H^{4+}]_t}{(1+K_{obs(Cl^-)}^{U,0}[Cl^-]+K_{X^-}^{U,0}[X^-]+K_{Y^-}^{U,0}[Y^-]+K_{Z^{2-}}^{U,0}[Z^{2-}])} \quad \text{(S20)}$$

In Eq. S20, $[H^{4+}]_t$ is known, as are the previously determined anion affinities $K_{Cl^-}^{U,0}$, $K_{X^-}^{U,0}$, $K_{Y^-}^{U,0}$, and $K_{Z^{2-}}^{U,0}$, What is unknown are the free (unbound) guest concentrations. Recall that $[G]_t = [HG] + [G]$ (Section 4.A.c), and that there are equivalent expressions for $[Cl^-]_t$, $[X^-]_t$, $[Y^-]_t$, $[Z^{2-}]_t$, respectively. Since $[G]_t = K_G[H][G] + [G]$, an expression can be derived for the free guest concentration, $[G]$, for any guest in terms of the total guest concentration ($[G]_t$), its association constant (K_G), and the free host concentration ($[H]$), Eq. S21 (Eq. 16 in the main text):

$$[G] = \frac{[G]_t}{(1+K_G[H])} \quad \text{(S21)}$$

Eq. S20 and Eq. S21 can be solved iteratively. Eq. S21 can be used to solve for the concentration of each free species ($[Cl^-]$, $[X^-]$, $[Y^-]$ and $[Z^{2-}]$) after substitution of the appropriate term for the guest, G, with Cl^-, X^-, Y^-, or Z^{2-}, respectively. These solutions can be used to solve Eq. S20, the solution of which, $[H^{4+}]$, in turn is used to solve Eq. S21 for each guest. In each case the total concentration of each guest is known. Importantly, $[Y^-]_t$ and $[Z^{2-}]_t$ remain fixed (large excess of buffer), and $[Cl^-]_t$

decreases during the (simulated) titration; specifically, $[Cl]_t = 4 \times [H^{4+}]_t$ for host **1**, which is incrementally adjusted due to dilution upon the (simulated) addition of the (buffered) guest solution. In each calculation the iterative process was carried out until the maximal change was < 0.0001.

Eq. S20–S21 were solved for $[H^{4+}]$, $[Cl^-]$, $[X^-]$, $[Y^-]$ and $[Z^{2-}]$) under three sets of simulated conditions. For reference, in each case the calculations involved buffer: 1) 10 mM phosphate pH 7.3 (45% $H_2PO_4^-$, 55% HPO_4^{2-}, *I* = 21.0 mM); 2) 23.8 mM phosphate buffer at pH 3.0 (12% H_3PO_4, 88% $H_2PO_4^-$, *I* = 21.0 mM) at same ionic strength of 1), and; 3) 10 mM phosphate buffer at pH = 3.0 (12% H_3PO_4, 88% $H_2PO_4^-$, *I* = 8.8 mM) maintaining the buffer concentration of 2), but at reduced ionic strength. In the latter two cases, neutral H_3PO_4 was assumed not to bind to the cationic host.

From the calculated free guest concentrations using Eq. S21, the concentration of each host-guest complex was determined (e.g. $[G]_t - [G] = [HG]$). The mole fraction definition (Eq. S15) was then used to generate population distributions of the host-guest complexes, which was used in concert with the observed δ_{max} from individual titrations of each guest (Section 4.A.c–e) to generate calculated Δδ values ($\Delta\delta_{calc}$) of the guest, X^-, in the presence of Cl^-, Y^- and Z^-. Excel's Solver function was then used to solve Eq. S20 by nonlinear regression and generate the least-squares best fit isotherm for $K^{U,0}_{pred}$ based on $\Delta\delta_{calc}$. Error estimates were determined using Eq. S22, where $CV\%^{X^-}$ is the individually determined error of the respective anion, and *CU%* is the combined (propagated) estimated (relative) error from each included anion.

$$CU\% = \sqrt{\sum_{i=1}^{n} \left[\left(CV\%^{X^-}\right)^2\right]_n} \quad \text{(S22)}$$

The predicted values ($K^{U,0}_{pred}$) and estimate of error (*CU%*) are summarized in Table S6. Here *CU%* is utilized to distinguish the estimated calculated error from the experimentally determined error (CV%) or the standard deviation (s). The inclusion of more terms when determining $K^{U,0}_{pred}$ results in a substantial increase in *CU* since for each determination of $K^{U,0}_{X^-}$ the individual errors (CV%) are propagated for *CU.* For example, the single calculation involving **1**.4Cl^- in 10 mM phosphate buffer, pH 7.3, with Br^- has associated errors (CV%) of Cl^- (7%), HPO_4^{2-} (9%), $H_2PO_4^-$ (10%), and Br^- (13%). The propagated error from Eq. S22 (*CU*%) for $K^{U,0}_{pred}$ is then ≈ 20%:

$$CU\% = \sqrt{\left(CV\%^{Cl^-}\right)^2 + \left(CV\%^{Br}\ \right)^2 + \left(CV\%^{HPO_4^{2-}}\right)^2 + \left(CV\%^{HPO_4^{2-}}\right)^2}$$

$$CU\% = \sqrt{(7\%)^2 + (9\%)^2 + (10\%)^2 + (13\%)^2}$$

$$CU\% = \sqrt{49\% + 81\% + 100\% + 169\%} = \sqrt{399\%} = 19.97\% \approx 20\%$$

Table S6: values ($K^{U,0}_{pred}$) for the binding of guests to **1**.4Cl⁻ (0.4 mM) under four sets of conditions. ($K^{U,0}_{pred}$ ± $\bar{CU}$) [a]

Guest	$K^{U,0}_{pred}$ (M^{-1}) to host 1.4Cl⁻		
	10 mM pH = 7.3[b]	23.8 mM pH = 3.0[c]	10 mM pH = 3.0[d]
I^-	4325 ± 810	5110 ± 810	7850 ± 1240
Br^-	630 ± 130	740 ± 150	1140 ± 200
Cl^-	120 ± 20	130 ± 20	180 ± 20
F^-	35 ± 7	40 ± 8	62 ± 12

a) Each calculated $K^{U,0}_{pred}$ value is based on solving Eq. S20 and Eq. S21 by an iterative process using the respective values of $K^{U,0}_{X^-}$ for each anion previously determined (Table S2 and Table S3). The error estimate ($\bar{CU}$%) was determined using Eq. S22 from the CV% of the respective anions. The absolute error shown is $\bar{CU}$; $\bar{CU}$ = $K^{U,0}_{pred}$ × $\bar{CU}$%.
b) 10 mM sodium phosphate buffer, pH 7.3 (*I* = 21 mM)
c) 23.8 mM sodium phosphate buffer, pH 3.0 (*I* = 21 mM)
d) 10 mM phosphate buffer, pH = 3.0 (*I* = 8.8 mM)

The values in Table S6 were determined by substitution into Eq S20 and Eq S21 under the three sets of conditions identified previously: 1) 4.5 mM $H_2PO_4^-$, 5.5 mM HPO_4^{2-}; 2) 2.8 mM H_3PO_4, 21 mM $H_2PO_4^-$; and; 3) 1.2 mM H_3PO_4, 8.8 mM $H_2PO_4^-$. Again, neutral H_3PO_4 was assumed not to bind to the cationic host. In the case of these complex systems (1, 2 and 3) the values for $K^{U,0}_{pred}$ were determined by substitution of the intrinsic anion affinities (Table S2) into Eq. S20 and Eq. S21 and the $K^{U,0}_{obs}$ value for the counterion into the simulated titration using the Solver function in Excel.[13] The given concentrations of the buffering anions were selected as constant values along with their intrinsic anion affinities (Table S3). Thus, the most complex prediction is case (1) involving four competitive anions: Cl^-, the added halide, $H_2PO_4^-$, and HPO_4^-.

The predicted values ($K^{U,0}_{pred}$, Table S6) are in very good agreement with the observed values ($K^{U,0}_{obs}$, Table S5). Thus, within error, the values for $K^{U,0}_{obs}$ agree with $K^{U,0}_{pred}$ in all instances, and the apparent attenuation in binding in multi-component systems can be attributed directly to competition by intrinsic chloride and phosphate. In other words, at these buffer concentrations ionic strength and screening effects do not influence binding.

We also determined the predicted and observed effect of increasing the host or host and buffer concentrations as shown in Table S7, also shown is the $K^{U,0}_{pred}$ when changing the counterion to Br^-. In the case of the simple system (D_2O, no buffer) the values for $K^{U,0}_{pred}$ were determined by substitution of the intrinsic anion affinities (Table S2) into Eq. S20 and Eq. S21 and the value for the counterion into the simulated titration using the Solver function in Excel. The value for $K^{U,0}_{obs}$ was determined by applying a simple 1:1 model (Eq. S11) neglecting the competing counterions. An increase in the initial host concentration of the unbuffered solution of host **1** from 0.4 to 1.0 mM (and correspondingly the Cl^- concentration to 4.0 mM) resulted in a similar attenuation of anion affinity. In this instance, the predicted affinity for Br^- was $K^{U,0}_{pred}$= 905 ± 134 M^{-1} and agreed with that obtained from titration experiments ($K^{U,0}_{obs}$ = 790 ± 58 M^{-1}). Furthermore, at 1.0 mM host **1** and 20 mM phosphate buffer (pH 7.3) $K^{U,0}_{obs}$ was further reduced (340 ± 44 M^{-1}) in good agreement with the calculated value ($K^{U,0}_{pred}$ = 346 ± 69 M^{-1}). Finally, the case of the titration of Br^- to host **1** (counterion, Br^-) in 10 mM phosphate buffer (pH 7.3). Using the known affinity for Br^-, $H_2PO_4^-$ and HPO_4^{2-} gave $K^{U,0}_{pred}$ ≈ 630 ± 130 M^{-1}.

Table S7: Comparisons of predicted binding constant ($K_{pred}^{U,0}$ and observed binding constant ($K_{obs}^{U,0}$) upon increased host concentration, or host and buffer concentration.

Conditions	Guest (X)	$K_{pred}^{U,0}$ (M^{-1})	$K_{obs}^{U,0}$(M^{-1})[a]
0.4 mM host **1** (counterion Cl^-) in D_2O	Br^-	1,320 ± 190	1290 ± 182 [b]
1.0 mM host **1** (counterion Cl^-) in D_2O	Br^-	905 + 134	790 ± 58 [b]
0.4 mM host **1** (counterion Cl^-) 10 mM phosphate buffer (pH 7.3)	Br^-	630 ± 130	738 ± 27 [c]
1.0 mM host **1** (counterion Cl^-) in 20 mM phosphate buffer (pH 7.3)	Br^-	346 + 69	340 ± 44 [b]
0.4 mM host **1** (counterion Br^-) 10 mM phosphate buffer (pH 7.3)	Br^-	650 ± 130	708 ± 26 [d]

a) Average values based on at least three determinations.
b) Obtained by fitting to simple 1:1 binding model (Eq. S11).
c) Obtained by competitive complexation with $K_{obs}^{U,0}Cl^- = 135\ M^{-1}$
d) Obtained by floating initial point and using Eq. S11.

5. References

1 Jordan, J. H., Wishard, A., Mague, J. T. & Gibb, B. C. Binding properties and supramolecular polymerization of a water-soluble resorcin[4]arene. *Organic Chemistry Frontiers* **6**, 1236-1243, doi:10.1039/c9qo00182d (2019).

2 Gibb, B. C., Chapman, R. G. & Sherman, J. C. Synthesis of Hydroxyl-Footed Cavitands. *The Journal of Organic Chemistry* **61**, 1505-1509, doi:10.1021/jo951633c (1996).

3 Tancini, F., Gottschalk, T., Schweizer, W. B., Diederich, F. & Dalcanale, E. Ion-pair complexation with a cavitand receptor. *Chem. - Eur. J.* **16**, 7813-7819, S7813/7811-S7813/7815, doi:10.1002/chem.201000573 (2010).

4 *BINDFIT*, http://supramolecular.org/.

5 Thordarson, P. Determining association constants from titration experiments in supramolecular chemistry. *Chemical Society reviews* **40**, 1305-1323, doi:10.1039/C0CS00062K (2011).

6 Gibb, B. C. & gibb, C. L. in *Supramolecular Chemistry* (eds Philip Gale & J.W. Steed) (2012).

7 Rahman, S. *et al.* Supramolecular host-guest complexation of Lash's calix[4]azulene with tetraalkylammonium halides and tetrafluoroborate salts: binding and DFT computational studies. *RSC Adv.* **5**, 54848-54852, doi:10.1039/C5RA07802D (2015).

8 Brynn Hibbert, D. & Thordarson, P. The death of the Job plot, transparency, open science and online tools, uncertainty estimation methods and other developments in supramolecular chemistry data analysis. *Chemical Communications* **52**, 12792-12805, doi:10.1039/C6CC03888C (2016).

9 Marcus, Y. *Ion properties*. (Marcel Dekker, 1997).

10 Pessêgo, M., Basílio, N., Muñiz, M. C. & García-Río, L. Competitive counterion complexation allows the true host : guest binding constants from a single titration by ionic receptors. *Organic & Biomolecular Chemistry* **14**, 6442-6448, doi:10.1039/C6OB00870D (2016).

11 Thordarson, P. *Binding Constants and their Measurements*. Vol. 2 239-274 (2012).

12 Gibb, C. L. D. & Gibb, B. C. in *Supramolecular Materials: From Molecules to Nanomaterials* (John Wiley and Sons, 2011).

13 In the case where the guest and counterion are identical, for determination of K_pred^(U,0), the counterion was treated strictly as a guest (X). Otherwise the unrealistic situation occurs where the counterions affinity is included twice: both as the counterion and as the guest, which gives an artifically suppressed value of K_pred^(U,0).

6. Appendix A

Solving for free host concentration [H] to find K_x. Equation S19.

For the competitive complexation model, a solution must be derived that solves for the free host concentration [H] in terms of the counterion and competing guest. Let [H] be the free host concentration, $[H]_t$ the initial or total host concentration, [Cl] the free concentration of the counter-ion (Cl^-), $[Cl]_t$ the total chloride counter-ion concentration, [X] the free guest (titrating)anion concentration $[X]_t$ the total guest (titrating)anion concentration, HCl the concentration of the host-chloride complex and HX the concentration of the host-guest complex. We arrive at the following expressions:

A1. $[H]_t = [H] + [HCl] + [HX]$
A2. $[Cl]_t = [Cl] + [HCl]$
A3. $[X]_t = [X] + [HX]$
A4. $K_{Cl} = \frac{[HCl]}{[H][Cl]}$
A5. $K_X = \frac{[HX]}{[H][X]}$

If we rearrange A2 and substitute into A4, we can obtain an expression for the free chloride concentration and an equivalent expression for the free guest concentration by substitution of A3 into A5:

A6. $[Cl] = \frac{[Cl]_t}{(K_{Cl}[H]+1)}$
A7. $[X] = \frac{[X]_t}{(K_X[H]+1)}$

And from substitution of A6 back into A2 and solving for [HCl] we obtain A8:

A8. $[HCl] = [Cl]_t - \frac{[Cl]_t}{(K_{Cl}[H]+1)}$

Substitution of A8 into A1 and solving for [HX] gives A9:

A9. $[HX] = [H]_t - [H] - [Cl]_t + \frac{[Cl]_t}{(K_{Cl}[H]+1)}$

We can substitute A7 and A9 into A5 to derive A10.

A10. $\frac{K_X[H][X]_t}{(K_X[H]+1)} = [H]_t - [H] - [Cl]_t + \frac{[Cl]_t}{(K_{Cl}[H]+1)}$

A10 can be solved for the free host concentration [H] in terms of a polynomial after distributing the terms and solving for zero which gives:

$$[H]^3(K_XK_{Cl}) + [H]^2\big(K_{Cl} + K_X + K_XK_{Cl}([X]_t + [Cl]_t - [H]_t)\big) + [H]\big(1 + K_X([X]_t - [H]_t) + K_{Cl}([Cl]_t - [H]_t)\big) - [H]_t = 0$$

Which has an expression of the form, $\alpha x^3 + \beta x^2 + \gamma x + \delta = 0$. Thus, we arrive at the cubic function A11:

A11. $\alpha[H]^3 + \beta[H]^2 + \gamma[H] + \delta = 0$

Where,

$\alpha = K_XK_{Cl}$
$\beta = K_{Cl} + K_X + K_XK_{Cl}([X]_t + [Cl]_t - [H]_t)$
$\gamma = 1 + K_X([X]_t - [H]_t) + K_{Cl}([Cl]_t - [H]_t)$
$\delta = -[H]_t$

7. Appendix B

Equations S20 & S21: Solving for free host concentration [H] and free guest [G] in multiple equilibrium.

We solve for the free host concentration $[H]$, where $[H]_t = [H] + [HG]$ to give:

B1. $[H] = [H]_t - [HG]$

Where $[H]_t$ is the total host concentration, and $[HG]$ is the concentration of the host-guest complex. From the definition of the equilibrium constant (K_{eq}) for any guest, G, we have $K_G = \frac{[HG]}{[H][G]}$, which gives the concentration of the host-guest complex $[HG]$:

B2. $[HG] = K_G[H][G]$

Substitution of B2 into B1 gives $[H] = [H]_t - K_G[H][G]$ and thus B3 by rearrangement to isolate [H]:

B3. $[H] = [H]_t/(1 + K_G[G])$.

Then $[H]$ can then be solved in terms of the known $[H]_t$, and unknown $[G]$, and its affinity, K_G. This open-form solution can be expanded to accommodate any number of guests. For multiple equilibria with three additional guests, X, Y, and Z, the equation for $[H]_t$ takes the form:

B4. $[H]_t = [H] + [HG] + [HX] + [HY] + [HZ]$.

Where, $[HX]$, $[HY]$, and $[HZ]$ are the concentrations of the host-guest complexes; each have the solution:

B5. $[HX] = K_X[H][X]$
B6. $[HY] = K_Y[H][Y]$
B7. $[HZ] = K_Z[H][Z]$

Substitution of these identity expressions into B4, gives B8, and isolating the term for $[H]$ gives B9:

B8. $[H]_t = [H] + K_{Cl}[H][Cl] + K_X[H][X] + K_Y[H][Y] + K_Z[H][Z]$.

B9. $[H] = \frac{[H]_t}{(1+K_G[G]+K_X[X]+K_Y[Y]+K_Z[Z])}$

Further, we have equivalent expressions for $[X]_t$, $[Y]_t$, $[Z]_t$:

B10. $[G]_t = [HG] + [G]$
B11. $[X]_t = [HX] + [X]$
B12. $[Y]_t = [HY] + [Y]$
B13. $[Z]_t = [HZ] + [Z]$

Substitution of B2 into B10 and isolating $[G]$ gives an expression for $[G]$ in terms of $[G]_t$, K_G, and $[H]$, again, with equivalent expressions for $[X]$, $[Y]$, $[Z]$ by substitution of B5, B6, and B7 into, respectively, B11, B12, and B13 to isolate the free guest term:

B14. $[G] = [G]_t/(1 + K_G[H])$
B15. $[X] = [X]_t/(1 + K_X[H])$
B16. $[Y] = [Y]_t/(1 + K_Y[H])$
B17. $[Z] = [Z]_t/(1 + K_Z[H])$

8) Appendix C

Screened binding worksheet description

Sheet *Binding*: On this sheet the user may specify the K0 binding constants for the ions F-, Cl-, Br-, and I- at the top of the page. The remaining sheets (F-, Cl-, Br-, I-, and NMR-fit) evaluate the distribution of bound and free ions in the systems considered and fit the concentrations of the bound ions to the experimental NMR delta-delta data. This sheet also reports the fitted delta-delta shift constants and fits to the NMR data. The overall error in the NMR fits is reported next to the binding constants. The user may also specify the Born radii for host, sodium, and the anions on this sheet.

Sheet *F-*: This sheet iteratively solves the distribution of free and bound ions using the Debye-Huckel screening model for the F-/Cl- competitive binding system.

Sheet *Cl-*: This sheet iteratively solves the distribution of free and bound ions using the Debye-Huckel screening model for the Cl- binding system.

Sheet *Br-*: This sheet iteratively solves the distribution of free and bound ions using the Debye-Huckel screening model for the Br-/Cl- competitive binding system.

Sheet *I-*: This sheet iteratively solves the distribution of free and bound ions using the Debye-Huckel screening model for the I-/Cl- competitive binding system.

Sheet *NMR-fit*: This sheet performs a least squares fit of the NMR shift constants for the L and J peaks of the bound ion data determined from the sheets F-, Cl-, Br-, and I- to the experimental results.
